# Proteins targeting ischaemic-reperfusion injury and repair after myocardial infarction: a systematic literature review

**DOI:** 10.1093/cvr/cvag100

**Published:** 2026-05-07

**Authors:** Asparuh Gardev, Derek J Hausenloy, Anton Pekcec

**Affiliations:** Boehringer Ingelheim International GmbH, Binger Straße 173, 55216 Ingelheim am Rhein, Germany; Cardiovascular & Metabolic Disorders Program, Duke-National University of Singapore Medical School, Singapore, Singapore; National Heart Research Institute Singapore, National Heart Centre, Singapore, Singapore; Yong Loo Lin School of Medicine, National University Singapore, Singapore, Singapore; The Hatter Cardiovascular Institute, University College London, London, UK; Boehringer Ingelheim Pharma GmbH & Co. KG, Cardio-Renal-Metabolic Disease Discovery Research, Birkendorfer Str. 65, 88397 Biberach, Germany

**Keywords:** Ischaemia-reperfusion injury, Myocardial infarction, STEMI

## Abstract

Following myocardial infarction (MI), reperfusion strategies ensure that blood flow is promptly restored to salvage the ischaemic myocardium. However, the sudden restoration of blood flow can inflict further damage to the ischaemic tissue, known as myocardial ischaemia/reperfusion injury (MIRI). Therapeutic strategies aimed at reducing MIRI and promoting cardiac repair remain an important clinical need. Protein-based therapies might exert benefits that limit damage and promote cardiac repair post-MI. To explore this further, we performed a systematic review of data from preclinical studies and clinical trial registries evaluating the cardioprotective effects of proteins post-MI. Medline and EMBASE were searched for preclinical studies that examined the potential cardioprotective effects of protein-based therapies to limit damage and/or mediate cardiac repair when administered after MI and/or reperfusion. Studies registered with ClinicalTrials.gov and EudraCT were also reviewed. In total, 84 studies were included in the final analysis, which included 46 different proteins. Overall, our findings support the concept that transiently applying recombinant or modified proteins after acute MI has the potential to promote lasting improvements in heart function via multiple pleiotropic mechanisms. Many of these proteins converge on a limited set of well-established signalling pathways involved in cardiac repair and remodelling after MI, with a few proteins demonstrating consistent and robust effects across multiple outcomes and models (small and large animals; reperfused and non-reperfused models). Despite this, the high potential cardioprotective benefit reported in preclinical studies has not translated into approved therapies for use in patients. Although the previous failure of protein therapies in clinical development does not invalidate the potential relevance of their downstream pathways, our research underlines the challenges in translating preclinical findings of protein-based therapies into the clinical setting. Future preclinical and clinical research should examine the optimal treatment effect and fully characterize the therapeutic potential of proteins involved in post-MI remodelling.

## Graphical Abstract

**Graphical Abstract:**
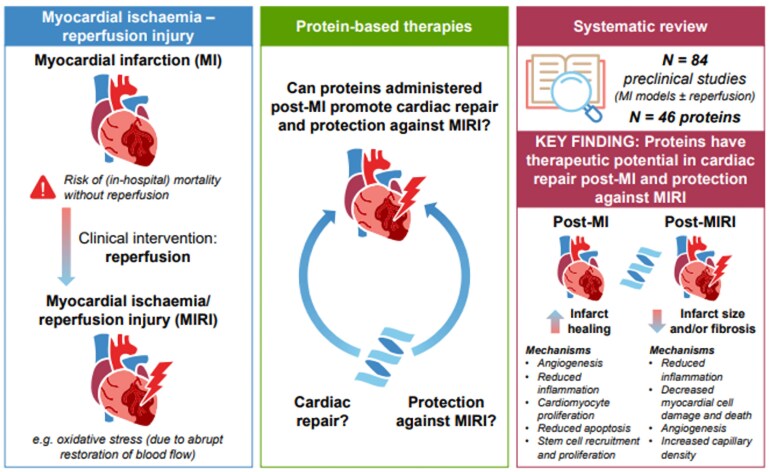

## 1. Introduction

With 197.2 million prevalent cases reported in 2019, ischaemic heart disease, including myocardial infarction (MI), remains a global burden and a leading cause of mortality.^1–3^ In patients with acute MI, prompt reperfusion and subsequent revascularization of the epicardial coronary artery is crucial for preventing microvascular dysfunction, an increase in infarct size, adverse cardiac remodelling, and, ultimately, heart failure (HF).^4^

Although reperfusion is critical for salvaging ischaemic myocardium, the abrupt restoration of blood flow to the myocardium during reperfusion can drive further damage to the ischaemic myocardium due to a series of events that characterize myocardial ischaemia/reperfusion injury (MIRI), including reactive oxygen species-related damage, intracellular calcium overload, endothelial dysfunction, mitochondrial dysfunction, cell death, and impaired microvascular flow.^5–7^ MIRI has not been addressed therapeutically, and strategies to reduce ischaemia/reperfusion injury remain an unmet clinical need.^7,8^

Following MI, a process of cardiac repair is initiated, leading to histological changes in the heart (myocardial remodelling), which can affect cardiac structure and function, and have a significant impact on patient outcomes. The cardiac wound healing response involves a complex interplay of cells, including cardiomyocytes, neutrophils, monocytes, macrophages, endothelial cells, and cardiac fibroblasts, which secrete a myriad of proteins essential for repair.^9^ Indeed, MIRI is associated with the secretion of proteins that act as intercellular communication players between the different cardiac cells to coordinate post-MI repair processes and promote myocardial healing.^10–13^ Proteins with known involvement in signalling pathways that promote cardiac repair or protect from MIRI include endogenous ligands, enzymes, small peptides, protein fragments, growth factors, receptors, cytokines, and extracellular matrix proteins.^10–13^ Therapeutic applications of proteins to supraphysiological levels, administered shortly after MI and/or reperfusion and more promptly than the endogenous protein, have demonstrated improvements in preclinical studies of MIRI and/or cardiac repair models. For example, growth factors such as cysteine-rich with endothelial growth factor (EGF)-like domains 2, triggering receptor expressed on myeloid cells 2, and myeloid-derived growth factor (MYDGF) have shown effects in mice on ischaemic tissue repair via different potential mechanisms of action, e.g. reduced cardiomyocyte death, reduced infarct scars, increased capillarization of the infarct border zone, enhanced endothelial cell proliferation, and increased sarcoplasmic or endoplasmic reticulum Ca^2+^-ATPase 2a expression in cardiomyocytes.^14–17^ These examples demonstrate the potential of protein-based therapies for ischaemic tissue repair via different mechanisms of action, meriting further investigation into how these findings may translate into larger mammals, including humans.

The aim of this systematic literature review is to highlight the recent research showing that (1) secreted proteins play vital roles in controlling MIRI and infarct healing, and (2) new therapeutic approaches may help reduce MIRI and/or improve infarct repair. To assess the therapeutic potential of protein-based therapies in ST-segment elevation MI (STEMI), we conducted a systematic review of the literature and examined the available evidence on the use of modified proteins and protein-based therapies to limit damage and/or promote cardiac repair in preclinical studies or in clinical trials.

## 2. Methods

### 2.1 The review protocol

We conducted a systematic review of published literature based on a prespecified protocol that has been registered in the International Prospective Register of Systematic Reviews under the registration ID CRD42024428589. An amendment to the review title and list of authors was submitted on 22 July 2025 (version 2.0). This review protocol conforms with the Preferred Reporting Items for Systematic Reviews and Meta-Analyses (PRISMA) guidelines 2020.^18^

### 2.2 Search strategy

An overview of the search strategy is presented in Supplementary material online, *Table S1*. First, a search of the Cochrane Library (https://www.cochranelibrary.com/) was carried out to confirm that no Cochrane reviews with overlapping scope have been published previously (see Supplementary material online, *Table S2*).

Search strings were created using combinations of terms designed to identify articles within the scope of this review. Specifically, articles describing clinical trials or preclinical (*in vivo* animal) studies that have investigated protein-based therapies for an ability to limit damage and/or mediate cardiac repair when administered after MI were selected (see Supplementary material online, *Tables S3* and *S4*). Relevant published or preprint articles released between 2 June 2018 and 2 June 2023 were identified in MEDLINE and Embase, restricted to publications available in English. The search was limited to a 5-year window to obtain a recent picture of the field and the most advanced protein-based technologies. Additional publications were retrieved through searching the citation lists of articles that qualified for inclusion through the title and abstract screen, described below.

Separate searches of ClinicalTrials.gov (https://clinicaltrials.gov/) and EudraCT (https://www.clinicaltrialsregister.eu/ctr-search/search) were conducted to identify ongoing clinical trials falling within the review scope (see Supplementary material online, *Tables S5* and *S6*).

### 2.3 Review process

A team of four reviewers independently screened the MEDLINE and Embase records, first by title and abstract. Records then underwent a full text review based on predefined eligibility criteria. Each publication was reviewed twice by two independent reviewers who were not part of the initial title and abstract screening team. Publications marked for inclusion at this stage were screened a final time by one of four independent reviewers (Kai C. Wollert, Mortimer Korf-Klingebiel, Marc Rene Reboll, or Lillian Hyde) to confirm the final inclusion list. Data extraction of articles marked for inclusion was then carried out, with each article screened twice. At all steps, disagreements were resolved by discussion. Ongoing clinical trials identified from the search of ClinicalTrials.gov and the EudraCT database were screened by one reviewer and checked by a second reviewer.

### 2.4 Study eligibility criteria

All clinical trials and preclinical studies that investigated protein-based therapies and showed evidence of an ability to limit damage and/or mediate cardiac repair when administered soon after MI were eligible for inclusion. Records were reviewed and excluded based on predefined exclusion criteria (see Supplementary material online, *Table S7*). If a study met more than one of the above exclusion criteria, one eligible criterion was selected at random for exclusion categorization.

### 2.5 Data extraction and synthesis

To capture data relevant to the scope of the review, we developed a standardized data extraction form that collected data relating to the specific protein-based therapy under investigation (specific protein, protein class, and molecular targets), the type of MI or MI model utilized, the investigated populations, the study design (clinical trial vs. preclinical model), the experimental protocol, the assessed clinical or physiological measures relevant to cardioprotection, and the study outcomes related to cardiac function, cardiac remodelling/structure, and other. For studies that described multiple experiments, only data from experiments that showed evidence of therapeutic benefit from protein-based therapy were extracted. A qualitative synthesis of data was performed, with the aggregation and tabulation of data according to study type (clinical trials vs. preclinical models) and protein class.

### 2.6 Convergence of signalling pathways among proteins

To evaluate shared mechanistic pathways and functional relationships, we captured information related to the mechanism(s) of action and signalling pathway(s) targeted by the protein-based therapies under investigation. Protein-based therapies were grouped according to the signalling pathways they targeted (if reported in the individual published studies), and the functions they regulated, such as angiogenesis, fibrosis, apoptosis, cell proliferation or differentiation, inflammation, vascular remodelling, cardiac function, and others. The aim of this analysis was to identify cardioprotective strategies/modalities that may be most relevant to the STEMI setting.

### 2.7 Protein framework analysis

We next developed a ranking framework to evaluate the effects of protein-based therapies across the different preclinical studies. This allowed us to compare protein-based therapies according to their cardioprotective potential and propose candidates or target pathways that represent greater promise for clinical translation. We first assigned a score to each protein according to their cardioprotective effects reported in the individual studies, using functional cardiac improvement [e.g. left ventricular (LV) ejection fraction (LVEF), fractional shortening (FS)] as the main parameter, followed by infarct size reduction and/or fibrosis reduction as secondary parameters, before considering molecular or cellular improvements (e.g. angiogenesis, apoptosis reduction, inflammation reduction, cell proliferation), and survival as a final parameter. We then applied a translational weighting to the protein-based therapies that demonstrated consistent and robust effects across multiple models, species, or studies. Scores from the outcome ranking and weighting factors were aggregated for each protein, and proteins with the highest scores were considered to represent therapies with the highest potential efficacy for myocardial repair and protection. The methodology used for the protein ranking framework can be found in the Supplementary material.

## 3. Results

### 3.1 Literature search

**Figure 1:**
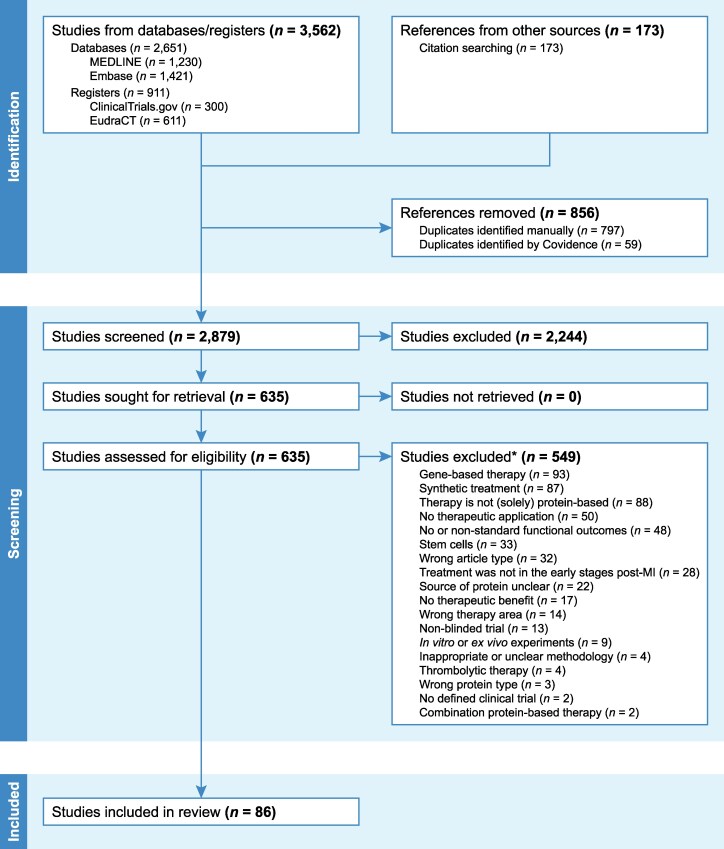
PRISMA flow diagram. Covidence is a screening and data extraction software used for conducting systematic reviews. *See Supplementary material online, *Table S7* in the supplementary material for details on the exclusion criteria.

Of the 2879 records that underwent title and abstract screening, 635 progressed to the full text review (*Figure 1*). We determined that 86 of these records met the eligibility criteria and marked these for inclusion. The full list of included records can be found in Supplementary material online, *Table S8*. The 86 included records comprised two ongoing clinical trials and 84 original research articles. Based on our prespecified search strategy, additional publications were retrieved through manual searching of the citation lists of the 84 original research articles, resulting in the inclusion of 42 additional papers published prior to 2018. This allowed us to identify potential proteins of interest, such as granulocyte colony-stimulating factor (G-CSF), from preclinical studies all published prior to 2018. These preclinical studies were deemed relevant since G-CSF was subsequently investigated in late-phase clinical trials in the acute MI setting.

### 3.2 Overview of protein-based therapies investigated post-MI

We identified 84 *in vivo* preclinical studies that used an ischaemic model of MI (with or without reperfusion) for investigating the ability of protein-based therapies to limit damage from MIRI and/or mediate cardiac repair when administered after MI (see Supplementary material online, *Tables S9–S11*). Of these, 29 studies used reperfused MI models that aimed to target MIRI through the administration of proteins, whereas 55 studies were conducted in non-reperfused ischaemic models focusing on infarct healing post-MI following administration of modified proteins, defined as endogenous proteins with modifications made to facilitate production, improve pharmacokinetics, and/or enhance safety. Two studies out of 84 targeted both pathologies, using both models of non-reperfused permanent ischaemia by ligation of the left coronary artery and reperfusion following ischaemia by occlusion of the left coronary artery.^19,20^

Overall, 46 different proteins were investigated across studies. All proteins are molecules that can be found in circulation and/or in various tissues, with known involvement in signalling pathways that promote cardiac repair or protect from MIRI, including endogenous ligands, protease inhibitors, enzymes, protein fragments, neuropeptides, growth factors, hormones, lipoproteins, and cytokines. Protein therapies were administered as intravenous infusions or subcutaneous injections, or via intracoronary/intramyocardial administration, and were either recombinant proteins (*n* = 59), synthesized and/or purified proteins or peptides (*n* = 21), or engineered proteins with modifications (fusion constructs, glycosylated proteins, other posttranslational modifications; *n* = 4). The most studied proteins were erythropoietin (EPO; *n* = 12), followed by apelin-13/apela (*n* = 6), G-CSF (*n* = 4), alpha-1 antitrypsin (AAT; *n* = 3), ghrelin (*n* = 3), and tissue inhibitor of matrix metalloproteinase-3 (TIMP-3; *n* = 3).

The main outcomes reported across all preclinical studies included the impact of treatment on cardiac function and remodelling, infarct size, and cardiac fibrosis. Cardiac function included assessment of LV end-diastolic or end-systolic diameter (LVEDD/LVESD), LVEF, FS, LV end-diastolic or end-systolic volume (LVEDV or LVVd/LVESV or LVVs), LV end-diastolic or end-systolic area (LVEDA/LVESA), and LV internal diameter at end-diastole or at end-systole (LVIDd/LVIDs), using imaging techniques (mainly echocardiography, cardiac magnetic resonance imaging, or angiography). Haemodynamic monitoring, largely using a pressure transducer with catheterization or pressure-volume loop, assessed changes in the LV end-diastolic pressure associated with treatment post-MI.

### 3.3 Proteins in small animal models of MI without reperfusion

The majority of studies—52 out of 84 preclinical studies—was conducted in non-reperfused MI models in small animals, focusing on infarct healing post-MI following administration of modified proteins (see Supplementary material online, *Table S9*). Myocardial ischaemia was induced through permanent ligation of the left coronary artery or left anterior descending coronary artery (*n* = 48), the proximal left coronary artery or left anterior descending coronary artery (*n* = 3), or the mid-left anterior descending coronary artery (*n* = 1), creating ischaemia from the lack of blood flow in the ligated area with little effect on the surrounding myocardial tissue.^21^ Administration of protein-based treatments occurred either during, immediately after, or several days post-MI, and were administered as single or multiple injections/infusions for several days/weeks post-MI. Some studies investigated various doses of a protein allocated to different treatment groups.

Most of the studies conducted in non-reperfused models of MI (44 out of 52) reported improvements in LV function, as preserved LVEF and/or FS observed in the groups treated with a protein-based therapy compared with control groups. Interestingly, the majority of these studies (22/44) also reported reductions in both end-systolic and end-diastolic dimensions of the LV, as smaller diameters, areas, or volumes, with five studies reporting smaller end-systolic (but not end-diastolic) dimensions of the LV, whereas three studies reported no change in LV function and/or end-systolic dimensions of the LV. Overall, these results indicate that most proteins under investigation had a significant and profound impact on both LV function and structure post-MI. This, however, did not translate into improved survival; of the 52 non-reperfused MI studies, 20 reported survival outcomes, with a reduction in mortality in the treatment groups compared with control groups reported in 11 studies, whereas 9 found no difference between groups. Nonetheless, the potential impact of protein-based therapies on reversing LV remodelling after STEMI is important, as increased LVEDV has been associated with a higher risk of adverse cardiovascular events in patients with STEMI.^22,23^

Improvements in LV structure and function were accompanied by a reduction in infarct size and/or fibrosis in the infarcted heart tissue after protein-based therapy compared with controls in 40 out of the 52 studies conducted in the non-reperfused small animal models, whereas a limited number of studies (3/52) reported an increase in scar thickness. Infarct expansion with reduced LVEF is a key feature of early adverse cardiac remodelling in the STEMI non-reperfused setting.^24^ Protein-based therapies administered post-MI promoted infarct healing through increased angiogenesis/revascularization, including increased capillary or microvessel density (*n* = 21), reduced myocardial cell death and/or apoptosis (*n* = 18), reduced inflammation (*n* = 9), increased cardiomyocyte or intracardial cell proliferation (*n* = 5), and stem cell recruitment/proliferation (*n* = 3), which are all hallmarks of cardiac repair.^25^

Angiogenesis is crucial in the restoration of microvasculature in the infarcted myocardium following STEMI. Of the 21 studies reporting an impact of protein-based therapies on angiogenesis, 16 different proteins were found to exert angiogenic effects, albeit via different mechanisms of action (several proteins were investigated in more than one study; see Supplementary material online, *Table S9*). For example, the administration of annexin A1 in mice resulted in an angiogenic response via production of vascular endothelial growth factor (VEGF)-A from cardiac macrophages leading to neovascularization and cardiac repair post-MI,^26^ whereas the administration of angiogenic VEGF-C promoted the upregulation of genes involved in lymphangiogenesis and led to an improvement in cardiac function.^27^ Mechanisms of action involved in cardiac repair can be complex and pleiotropic in nature. Daily injection of the fusion protein Fc-ELA-21 in MI rats for 4 weeks exerted both angiogenic and anti-apoptotic effects with cardiomyocyte proliferation and reduced cardiac fibrosis in MI hearts,^28^ whereas recombinant irisin treatment administered in mice for 2 weeks post-MI reduced cardiomyocyte apoptosis and fibrosis with pro-angiogenic effects that were associated with the extracellular signal-regulated kinase (ERK)-dependent pathway.^29^ Other actions of protein-based therapies may be related to their ability to promote cell proliferation and differentiation, thus indirectly contributing to the angiogenic response observed in some of the non-reperfused MI model studies. Recombinant EPO has been shown to promote cardiac repair post-MI through increased proliferation, migration, and clone formation of stem cell antigen 1 positive stem cells via the EPO receptor and downstream signal transducer and activator of transcription 5 (STAT-5)/p38 mitogen-activated protein kinase (MAPK) signalling pathways,^30^ or through targeted migration of stem/progenitor cells via the enhanced expression of chemoattractant stromal cell-derived factor 1.^31^ Meteorin-like protein plays a role in the crosstalk between myeloid cells and endothelial cells by activating receptor tyrosine kinase at the surface of cell populations in the infarct border zone, promoting an angiogenic response linked to infarct repair.^32^ Additionally, other proteins, including gastrin,^33^ G-CSF,^34^ oncostatin M,^35^ interleukin (IL)-10,^36^ IL-19,^37^ and IL-37,^38^ have been found to promote cardiac repair through angiogenesis, reduced myocardial cell death/apoptosis, reduced fibrosis, and/or reduced inflammatory response, emphasizing the importance of targeting pathways with pleiotropic effects in the STEMI setting.

Although inflammation is necessary for initiating cardiac repair after ischaemia, extensive inflammation is thought to drive fibrosis.^39^ Post-MI administration of recombinant interleukins in mice can suppress the inflammatory response, attenuate adverse ventricular remodelling, and improve cardiac function through the polarization of macrophages with anti-inflammatory activity,^37,40^ and/or activation of the downstream signal transducer and activator of transcription pathway.^36,37,40,41^ Other proteins exhibited anti-inflammatory properties with attenuated MI-induced cardiac dysfunction. AAT, a naturally occurring serine protease inhibitor, administered in mice post-MI prevented adverse cardiac remodelling through reduced activity of the proinflammatory capsase-1 in the ischaemic myocardium.^20^ Hormones such as apelin-13 (an adipokine, endogenous ligand of angiotensin II receptor-like 1), and cholecystokinin and ghrelin (both produced by enterocytes of the gastrointestinal tract) have demonstrated post-MI attenuation of cardiac remodelling and/or fibrosis through inhibition of nuclear factor kappa-light-chain-enhancer of activated B cells (NF-κB) signalling,^42^ transforming growth factor (TGF)-β1 and connective tissue growth factor,^43^ and IL-1β and tumour necrosis factor (TNF)-α.^44^ Klotho, a protein linked to the ageing process in humans, administered intraperitoneally to mice 1 week post-MI every other day for 28 days, demonstrated a wide range of cardioprotective effects, including improved cardiac function, reduced cardiac fibrosis, and attenuated myocardial inflammatory factors, ferroptosis, and oxidative stress through regulation of the adenosine monophosphate-activated protein kinase (AMPK)/mechanistic target of rapamycin (mTOR) signalling pathway.^45^

While most studies started administration of protein-based therapies immediately post-MI, a few studies investigated the effect of dose timing and dose frequency on the improvement of LV function. Administration of glial growth factor 2 (GGF2) intravenously from days 8–28 post-MI at dosing frequencies of once every 24, 48, or 96 h all resulted in improved LVEF and FS and reduced LVESV at day 28 and day 38 (10 days after treatment end) post-MI compared with control (treatment with vehicle); improvements in LV were dose-dependent.^46^ In another study, LV function was improved after administration of two different doses of GGF2 (0.625 mg/kg or 3.25 mg/kg) to rats at day 7 post-MI and continuing for 4 weeks, compared with the vehicle-treated MI group; when administration of both doses was delayed until 8 weeks after MI, only the highest GGF2 dose was associated with improved function.^47^ Both of these studies highlight the need for further research on the most appropriate dose, timing, and frequency of protein-based therapy.

### 3.4 Proteins in small animal models of MI with reperfusion

Of the 84 preclinical studies included in our analysis, 23 used models of ischaemia/reperfusion in small animals and aimed to target MIRI through the administration of protein-based therapies (see Supplementary material online, *Table S10*). The use of protein-based therapies in the MIRI setting is clinically relevant given that most infarct patients present with acute coronary artery occlusion and undergo reperfusion by either fibrinolytic therapy or primary percutaneous coronary intervention (PCI). Ischaemia was induced by left coronary artery/left anterior descending coronary artery ligation or occlusion, generally lasting between 25 and 60 min. Reperfusion ensued following release of the occlusion/ligation and lasted from 1 h up to 3 months. Administration of protein-based treatments occurred either prior or immediately after reperfusion, whereas some were administered with a delay of up to 24 h and others were administered for several days to weeks post-MI.

Cardiac function was assessed through changes in LVEF and FS, with 13 out of 23 studies reporting improvements in LVEF and/or FS in protein-based treatment groups compared with control groups. This was accompanied by reductions in diameter, volume, and/or pressure of the left ventricle (*n* = 9), suggesting a positive impact of protein-based therapies on LV remodelling after MIRI. Only six studies reported survival outcomes following MIRI, of which three reported reduced mortality in the protein-based treatment groups compared with controls, whereas three studies showed no difference between groups.

Of the 23 MIRI studies, 22 reported a reduction in infarct/scar size and/or fibrosis associated with protein therapies compared with controls, whereas two studies reported no difference between groups. The ability to reduce infarct size is a clear therapeutic advantage, considering that in animal models, MIRI accounts for up to 50% of the final myocardial infarct size.^48^ The pathophysiology of MIRI is complex and involves multiple mechanisms related to cardiomyocyte death, such as necrosis, apoptosis, necroptosis, and pyroptosis, as well as coronary microvascular injury, requiring additive cardioprotective interventions aimed at reducing infarct size.^49^ The reported reductions in infarct size and/or fibrosis observed in the MIRI models were accompanied by reduced inflammation (*n* = 8), reduced myocardial cell death and apoptosis (*n* = 8), increased angiogenesis (*n* = 6), reduced myocardial damage (*n* = 3), and improved capillary density (*n* = 5).

The pleiotropism that most proteins exhibit is an important factor influencing the cardioprotective properties of a protein-based therapy against MIRI. Apelin-13 has demonstrated not only an improvement in myocardial function through neovascularization,^50^ but also inhibition of endoplasmic reticulum stress-induced apoptosis via pharmacological activation of phosphatidylinositol-3-kinase (PI3K)/protein kinase B (Akt), AMPK, and ERK pathways.^51^ When administered at reperfusion, plasma-derived AAT has shown a >50% reduction in infarct size following 30 min of ischaemia in mice.^52^ In another study, AAT-treated mice had significantly smaller infarct sizes at day 1 (−30%) and day 7 (−55%) compared with mice treated with control (albumin) while exhibiting anti-inflammatory properties with a >90% reduction in caspase-1 activity measured in cardiac tissue homogenates 24 h after ischaemia-reperfusion (I/R) injury.^20^

Several secreted proteins, such as MYDGF and endoplasmic reticulum membrane protein complex subunit 10 (EMC-10), are paracrine-acting proteins produced by bone marrow-derived cells that can modulate LV remodelling and systolic dysfunction, reduce infarct size, and promote angiogenesis, as shown in preclinical studies.^15,53^ MYDGF treatment at reperfusion and for 7 days demonstrated cardioprotective effects mediated by PI3K and Akt signalling pathways, promoting endothelial cell proliferation and capillary density in the infarcted region.^15^ Treatment with recombinant EMC-10 for 7 days post-MI exhibited angiogenic properties with enhanced infarct border-zone capillarization through activation of the p38 MAPK–MAPK-activated protein kinase 2 pathway to promote actin polymerization and endothelial cell migration.^53^ Other cardioprotective effects during reperfusion include inhibition of apoptosis and inflammation. Short-term treatment with insulin-like growth factor 1 (IGF-1) for 3 days at the start of reperfusion post-MI improved cardiac function after 1 and 4 weeks, which was attributed to an M2-like anti-inflammatory phenotype induced in bone marrow-derived macrophages that enhanced the number of anti-inflammatory macrophages in heart tissue on day 3 post-MI.^54^ Neurotrophin-3 is a growth factor of the neurotrophin family with cardiovascular function and cardioprotective effects against MIRI through the ERK-Bim signalling pathway and the promotion of angiogenesis in I/R hearts that together result in improved cardiomyocyte survival.^55^ The fibrin-derived Bβ_15–42_ peptide administered at reperfusion has demonstrated anti-inflammatory properties by significantly blunting inflammation during MIRI.^56^

In order to simulate real-world conditions with treatment not being available at the time of reperfusion, several studies investigated the impact of delaying treatment with protein-based therapies after the start of reperfusion. Delaying treatment with MYDGF therapy until 6 h after initial reperfusion still reduced scar size and improved systolic function in mice 28 days after reperfusion.^15^ Compared with vehicle treatment, administration of prolastin C, plasma-derived AAT, at the highest dose tested (180 mg/kg) given 30 min after reperfusion, showed a significant reduction in infarct size at 24 h, comparable to the reduction in infarct size seen when given without a delay.^52^

### 3.5 Proteins in large animal models of MI

A small number of studies—9 out of 84—were carried out in large animals (pigs, *n* = 8; dogs, *n* = 1), of which 6 were based on MIRI models and 3 were non-reperfused MI studies (see Supplementary material online, *Table S11*).

In the non-reperfused setting, two studies investigated the effects of EPO after MI,^57,58^ and one study assessed whether recombinant TIMP-3 altered post-MI remodelling.^59^ In female domestic pigs, subcutaneous injections of recombinant human epoetin (EPO) did not significantly change LVEF and LVEDV at 28 days post-MI compared with the injection of saline in the control group. However, there was a documented upregulation of angiogenic factors, such as increased serum levels of hepatocyte growth factor and fibroblast growth factor, as well as local mRNA expressions of VEGF and IGF in the border and infarcted area of the EPO-treated groups.^58^ In dogs, the intravenous injection of recombinant human EPO administered immediately post-MI significantly increased LVEF and reduced LVEDD at 1 and 4 weeks post-MI, and reduced infarct size at 4 weeks post-MI when compared with the injection of saline in the control groups.^57^ The same study also investigated the effects of delayed EPO administration, with a 6-h delay producing positive changes in LV function at 4 weeks post-MI compared with control, but not when administered with a 1-week delay. Neither time delay had an impact on infarct size. EPO administered immediately post-MI or with a 6-h delay increased capillary-to-myocyte ratio corrected for LV hypertrophy, capillary density, and circulating CD34-positive mononuclear cell numbers, suggesting a role for EPO in neovascularization in the ischaemic region via endothelial progenitor cells mobilization that remains beneficial even when administered with a delay of 6 h after reperfusion intervention.^57^

Myocardial injections of TIMP-3 in pigs immediately post-MI resulted in higher LVEF at 14 days post-MI compared with injection of saline in the control groups, whereas TNF and IL-10 mRNA expressions decreased. The study also demonstrated that although two different formulations of TIMP-3 (namely, full recombinant molecule or truncated form encompassing the N-terminal) favourably impacted LV remodelling, only the full recombinant TIMP-3 reduced the expression of fibrillar collagen and indices of inflammation post-MI, suggesting a role for the C-terminal of TIMP-3 in exerting other biological processes that may be beneficial in MI.^59^ In the MIRI setting, the intracoronary infusion of recombinant TIMP-3 during the final 4 min of an ischaemic period, prior to reperfusion, resulted in increased LVEF and FS, and reduced LVEDV at 28 days post-MI in the TIMP-3-treated vs. control (saline injection) groups. This also reduced infarct size and fibrosis.^60^ Results also showed a reduction in plasma NH2-terminal pro-brain natriuretic peptide levels in the TIMP-3 group compared with control, suggesting a role for matrix metalloproteinase inhibition in reversing adverse LV remodelling and slowing HF progression.^60^

Other protein-based therapies being investigated in the MIRI setting included agrin, a large extracellular matrix (ECM) fragment,^19^ G-CSF,^61^ IGF-1,^62^ and platelet-derived growth factors.^63,64^ Infusion of recombinant human agrin immediately after reperfusion in pigs demonstrated improvements in LV function and prevention of adverse remodelling through reduction of interstitial fibrosis but not infarct size compared with control (saline infusion).^19^ Additionally, agrin infusion resulted in an increase in endothelial cell number in both border zone and infarct region at 28 days after MI, a reduced number of activated macrophages (CD68^+^ macrophage/monocyte marker and CD14^+^ macrophage marker) in the border zone sections, as well as a significant increase in cardiomyocyte cell cycle re-entry measured by Ki67 staining in both border zone and infarct regions of the pig hearts treated with recombinant human agrin. Together, these results suggest a protective role of agrin through pleiotropic effects that include promoting cardiomyocyte protection, inducing angiogenesis, attenuating the inflammatory response, and moderating cardiomyocyte proliferation.^19^

Intracoronary administration of recombinant human IGF-1 post-reperfusion following an MI in pigs resulted in improvements in LV function and remodelling, as seen with reductions in LV volumes and significant improvement in LV systolic and diastolic function. This was achieved through a reduction in infarct size and attenuation of wall thinning compared with saline treatment (control) via cardioprotective effects mediated by the phosphorylation of survival-associated protein kinases (Akt and ERK) and GSK-3β in infarct tissue, leading to the attenuation of cardiomyocyte cell death.^62^

Therapy with recombinant human platelet-derived growth factor-AB compared with control (untreated or vehicle) in pigs post-MI and ischaemia–reperfusion demonstrated pleiotropic effects beyond that of fibroblast proliferation and fibrosis towards an effective reparative process of post-MI scar maturation. These effects included reduced myofibroblast differentiation, alteration of ECM synthesis favouring a protein profile that is expressed during the proliferative phase of MI repair, increased migration without increased proliferation, and increased myocardial perfusion via neovascularization.^63,64^

G-CSF administration post-MI in a porcine ischaemia–reperfusion model exerted beneficial effects on LV remodelling by decreasing LV diastolic dilatation compared with the untreated control group.^61^ In contrast, the beneficial effect of G-CSF on LV function and remodelling was attenuated when the initiation of therapy was delayed by 5 days post-MI, as evidenced by decreased LVEF, increased normalized infarct mass, and increased infarct expansion compared with the control group. There was no difference in collagen types or inflammatory activity in the mid-scar region between the two treatment groups, with a scar predominantly composed of fibroblasts and few cardiomyocytes. Additionally, delayed treatment may also impair myocardial vasculature by reducing arteriolar density, as observed in the mid-scar zone in the delayed treatment group, thereby directly impacting scar size and LV remodelling.^61^

### 3.6 Signalling pathways involved in promoting cardioprotection and cardiac repair following cardiac ischaemia

**Figure 2:**
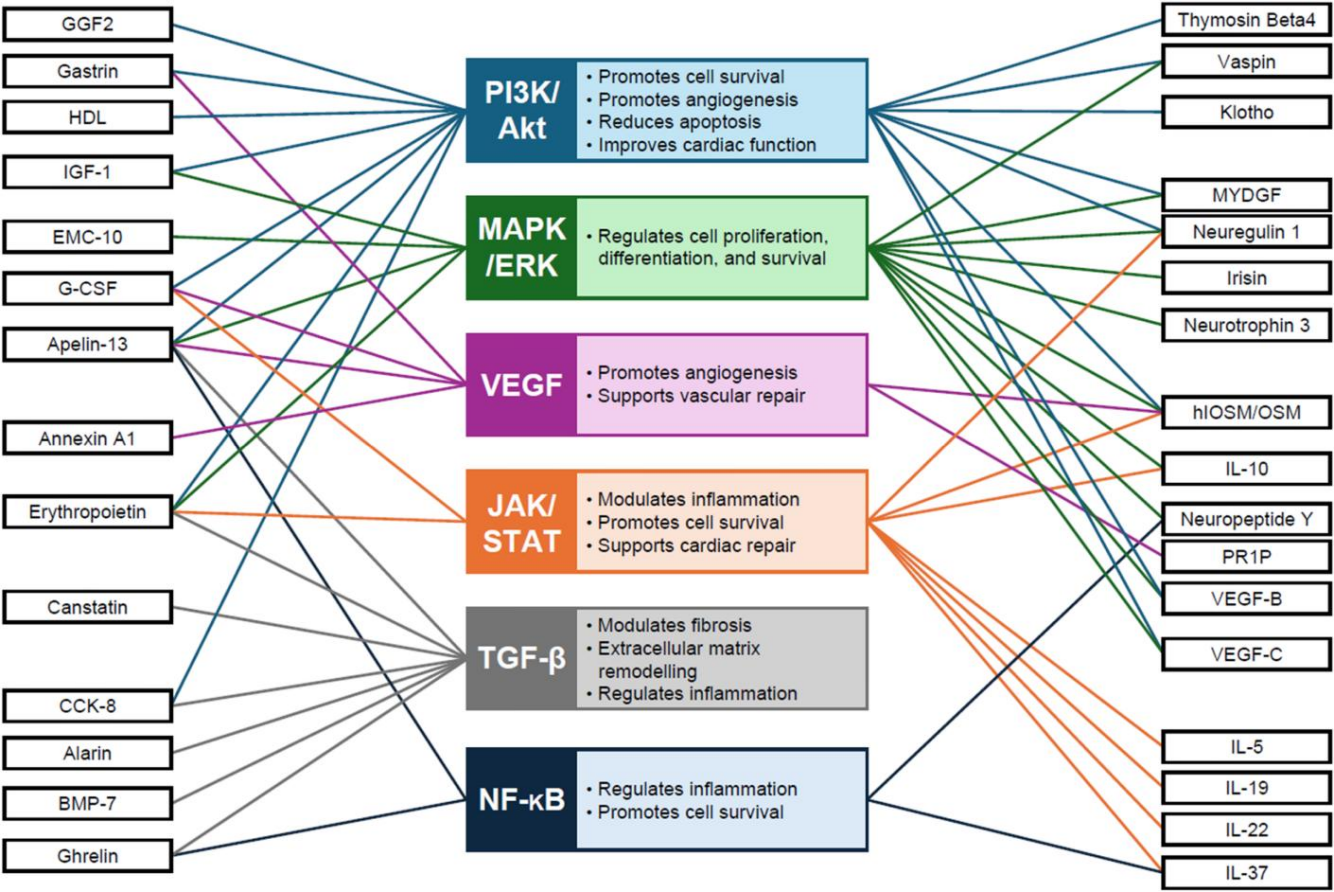
Convergence of signalling pathways and functional relationships of protein-based therapies involved in cardiac repair post-MI and cardioprotection against MIRI, based on the MI model studies included in this review. BMP-7, bone morphogenetic protein-7; CCK-8, cholecystokinin 8; EMC-10, endoplasmic reticulum membrane protein complex subunit 10; ERK, extracellular signal-regulated kinase; G-CSF, granulocyte colony-stimulating factor; GGF2, glial growth factor 2; HDL, high-density lipoprotein; hlOSM, human-like oncostatin M; IGF, insulin-like growth factor; IL, interleukin; MAPK, mitogen-activated protein kinase; MI, myocardial infarction; MIRI, myocardial ischaemia/reperfusion injury; MYDGF, myeloid-derived growth factor; OSM, oncostatin M; PR1P, prominin-1-derived peptide; TGF-β, transforming growth factor beta; VEGF, vascular endothelial growth factor.

Multiple signalling pathways have been associated with pathological characteristics in MI, and it is the convergence of these diverse biological pathways, rather than a common pathway of mechanisms, that promotes myocardial damage and establishes a vicious cycle of progressive loss of cardiac function.^65,66^ Many of the 46 proteins included in our analysis converged on a limited set of well-established signalling pathways involved in cardiac repair and remodelling after MI. These pathways, primarily PI3K/Akt, Janus kinase (JAK)/STAT, MAPK/ERK, TGF-β, VEGF, and NF-κB, regulate known key processes in cardiac repair post-MI, including angiogenesis, anti-apoptosis, anti-fibrosis, immunomodulation, and progenitor cell recruitment.^65–67^ Sixteen of the proteins studied targeted more than one pathway (*Figure 2*).

Based on this pathway analysis, it is clear that the PI3K/Akt and MAPK/ERK pathways appear most relevant as potential therapeutic targets to promote cardioprotection and cardiac repair following MI. Proteins such as apelin-13/apela, cholecystokinin 8, gastrin, klotho, vaspin, MYDGF, or VEGF-B (among others) all converge on the Akt pathway and were reported to promote cell survival and angiogenesis, reduce apoptosis, and improve cardiac function in the preclinical experiments included in our analysis. A similar role has been reported in a model of renal ischaemia–reperfusion with pharmacological activation of Akt by recombinant human MYDGF, leading to reduced oxidative stress, inflammation, and apoptosis,^68^ suggesting a promising role for protein-based therapies that target ischaemia/reperfusion via the Akt pathway. In our analysis, proteins that were reported to promote cell proliferation, differentiation, and survival via the MAPK pathway included EPO, IL-10, oncostatin M, and neuregulin 1 (among others). The ERK signal pathway is one of the MAPK signalling pathways that triggers a tertiary enzymatic cascade of MAPKs, promoting angiogenesis, cell growth, and proliferation through a phosphorylation response.^69^ Another convergent pathway shared by several proteins in our analysis was the JAK/STAT signalling pathway, which plays a key role in MI by regulating inflammation, apoptosis, fibrosis, and angiogenesis,^70^ which are crucial for limiting MIRI. Bone morphogenetic protein-7 (BMP-7), canstatin, EPO, apelin-13/apela, ghrelin, alarin, and cholecystokinin 8 (CCK-8) all showed an impact on fibrosis, extracellular matrix remodelling, and inflammation in our analysis. These proteins act via the TGF-β pathway (*Figure 2*), which is known for its role in driving fibrosis and controlling cardiac remodelling.^71^ Through VEGF-related signalling, several proteins, namely apelin-13/apela, oncostatin M (OSM), G-CSF, and prominin-1-derived peptide, were reported to promote angiogenesis and vascular repair, likely through improved perfusion to the injured myocardium.^72^ Lastly, apelin-13/apela, IL-37, ghrelin, and neuropeptide Y (NPY) were reported to regulate inflammation and cell survival. This occurs through suppression of the NF-κB pathway, which is involved in excessive inflammation and oxidative stress,^73^ thus preventing further myocardial damage at reperfusion. In summary, this convergence of pathways suggests that despite the diversity of proteins, many orchestrate repair through shared downstream effectors that often target common molecular mechanisms for myocardial repair and protection. Consequently, multitarget cardioprotective strategies/modalities focused on these key molecular mechanisms may be most appropriate for further clinical development in STEMI.

### 3.7 Framework analysis: role of proteins as potential therapies of ischaemic repair and cardioprotection against MIRI

An analysis of protein-based therapies was conducted to identify candidates with the most promising potential efficacy reported in the preclinical studies included in our review (see Supplementary material online, *Table S12*). EPO, apelin-13/apela, G-CSF, MYDGF, and IL-37 demonstrated consistent and robust effects across multiple outcomes and models. EPO was the protein most studied preclinically and was associated with consistent improvements in LVEF, FS, and reduction in infarct size or fibrosis across multiple studies and species, as well as demonstrating a survival benefit (in 1 out of 12 studies) and improved cardiac remodelling in both small and large animal models. The multiple signalling pathways involved included JAK2/STAT5, PI3K/Akt, MAPK, endothelial nitric oxide synthase (eNOS), and TGF-β/WNT, resulting in angiogenesis, stem cell mobilization, and anti-apoptotic effects. Apelin-13/apela showed consistent efficacy across multiple studies and models, with strong improvements in LVEF, FS, and reduction in fibrosis and infarct size. Through modulation of the apelin receptor (APJ), and PI3K/Akt, MAPK, and NF-κB pathways, apelin-13/apela is known to promote angiogenesis, reduce apoptosis, and activate cardiac stem/progenitor cells. G-CSF acts via the JAK/STAT and Akt/VEGF pathways to promote angiogenesis and stem cell mobilization and reduce apoptosis. In the studies included in our analysis, G-CSF showed marked improvements in LVEF, FS, infarct size reduction, as well as survival benefits and improved cardiac function. However, the cardioprotective effects of G-CSF were only observed in small animals; administration of G-CSF in pigs at reperfusion showed no impact on LV function or remodelling and also resulted in decreased capillary density in the peri-infarct border zone.^61^ MYDGF, acting via the c-Myc/FoxM1, PI3K-Akt, and MAPK-STAT3 pathways, demonstrated reduction in infarct size and fibrosis, LVEF improvement, cardiomyocyte proliferation, and improved survival in two studies of small animal MI/non-reperfusion and MIRI.^15,74^ IL-37 has previously demonstrated strong anti-inflammatory and anti-apoptotic effects, and has therefore been considered a promising therapeutic target and biomarker in inflammatory diseases, autoimmune diseases, and cancer.^75^ In two studies of small animal MI/non-reperfusion and MIRI, IL-37 reduced infarct size, fibrosis, and inflammation, and improved LVEF and survival.^38,76^ Other IL candidates, namely IL-19, -22, -5, and -10, also ranked highly in our protein ranking framework analysis (see Supplementary material online, *Table S12*), emphasizing a strong inflammatory component in the cardioprotective mechanism of protein-based therapies.

Together, these results from several large animal models of MI and MIRI suggest that some protein-based therapies may have pleiotropic effects promoting cardiac healing post-MI and/or exerting cardioprotection against MIRI, via multiple mechanisms. Some of the proteins investigated in mechanistic and preclinical studies and discussed in the sections above have been investigated in clinical trials. We summarize results from recent trials and ongoing clinical trials of some of these protein-based therapies below.

### 3.8 Proteins investigated in MI clinical trials

Published results from trials investigating the efficacy and safety of protein-based therapies in patients with STEMI are summarized in Supplementary material online, *Table S13*, including EPO, fibrin-derived peptide Bβ_15–42_, G-CSF, and plasma-derived AAT. In this review, EPO was one of the main proteins studied preclinically, but the administration of EPO-α within 4 h of reperfusion in patients with STEMI did not impact infarct size or LVEF compared with placebo (see Supplementary material online, *Table S13*).^77^ Similarly, when EPO-β was administered in patients with acute STEMI treated immediately, and 24 and 48 h after primary PCI, there were no significant changes in 6-month LVEF or infarct size compared with placebo (see Supplementary material online, *Table S13*).^78^ This was confirmed in a long-term follow-up analysis, in which the combined incidence of major adverse cardiovascular events (MACE) 5 years after randomization occurred in 25% of the patients assigned to EPO-β and 17% of the patients assigned to placebo (rate ratio 1.5; 95% confidence interval 0.8, 3.5; *P* = 0.26).^79^ A meta-analysis including 10 randomized clinical trials investigating the effects of erythropoiesis-stimulating agents (ESA) in STEMI patients undergoing primary PCI has not shown a significant difference in the composite primary endpoint of all-cause mortality, MI, and stent thrombosis after PCI or all-cause mortality in the ESA group compared with the control group.^80^ Similar results were described in another systematic review and meta-analysis, in which the outcomes from 15 randomized controlled trials of EPO administration following PCI failed to demonstrate protective effects on LV function, infarct size, stroke, re-MI, HF, mortality, thrombosis, or MACE.^81^

In a Phase 2 trial including patients presenting with acute STEMI, the administration of an intravenous bolus of FX06, a naturally occurring peptide derived from human fibrin, at reperfusion did not significantly reduce infarct size or LVEF at 5 days compared with placebo, but resulted in a reduced necrotic core size.^82^

In the Phase 3 STEM-AMI OUTCOME trial, patients with STEMI who received G-CSF on top of standard of care (SoC) showed significant improvement in LVEF changes from baseline to 6 months compared with SoC alone, as well as a greater reduction in indexed LVESV in the G-CSF and SoC group compared with SoC alone.^83^ An earlier trial of patients diagnosed with STEMI with successful reperfusion by PCI did not demonstrate any impact of a daily dose of G-CSF (10 μg/kg) administered for 5 days on infarct size, LV function, or coronary restenosis compared with placebo.^84^ In contrast, a twice-daily administration of G-CSF (5 μg/kg) started within 12 h of PCI in patients with anterior STEMI resulted in attenuated LV remodelling compared with placebo by preventing increases in LVEDV and LVESD at 6 months from baseline.^85^

The impact of prolastin C (plasma-derived AAT) administration on the inflammatory response was assessed in patients with STEMI by comparison with historical controls, and significantly lowered the area under the curve of C-reactive protein levels 14 days after admission, suggesting a role of prolastin-C in blunting the acute inflammatory response following STEMI.^86^

### 3.9 Key recommendations and pitfalls in reporting basic research findings

Despite the positive results from preclinical models, and although the study of protein-based therapies in the STEMI/MIRI setting has been active over the last two decades and demonstrated potential clinical effects, there are currently no approved drugs in this setting.^87^ Position papers have discussed reasons for the failure to effectively translate potential cardioprotective therapies and made recommendations for improving its translation into the clinical setting.^88–90^ Working groups of the National Heart, Lung, and Blood Institute^88^ and the European Society of Cardiology^89^ highlighted that inefficiencies in basic research, and a lack of standardized, multicentre preclinical testing have hindered progress, advocating for rigour comparable to clinical trials and recommending the establishment of a national multicentre preclinical consortium to improve reliability and reproducibility. The IMproving Preclinical Assessment of Cardioprotective Therapies (IMPACT) criteria provide key recommendations and criteria to address the lack of reproducibility in basic research findings.^91^ In IMPACT, the animal multisite acute MI network used centralized randomization and blinded core infarct size analysis, as well as validation steps using ischaemic preconditioning as a common treatment. This yielded an improvement in the reproducibility of cardioprotective interventions in preclinical studies across study sites and different animal species.^92,93^ In our analysis of the literature, although some protein-based therapies were investigated in multiple individual studies and across MI models, these were often carried out in single-centre laboratories without a concerted or inter-connected approach that would likely strengthen the evidence.

The National Heart, Lung, and Blood Institute Working Group stated that ‘almost all studies of cardioprotection and cardiac arrest in experimental models have yielded inconsistent and/or unreproducible results’.^88^ To improve the preclinical evaluation of novel cardioprotective interventions, Lecour *et al*. proposed key recommendations for the design of rigorous preclinical experiments in the ischaemia/reperfusion injury setting.^91^ For example, the guidelines recommend that sample size is determined prior to conducting an experiment to appropriately assess the effect size, animals are randomized to treatment groups to avoid bias, treatment allocation and analysis are assessed in a blinded manner, and that study endpoints are harmonized across studies, using infarct size as the gold standard (or microvascular obstruction). These criteria are also detailed in the ARRIVE guidelines to ensure transparency and consistency in the reporting of animal research.^94^ Many of the studies included in our analyses did not report information relating to randomization, allocation concealment, random housing blinding, or inclusion/exclusion criteria, making the assessment of potential risk of bias related to study design difficult. Additionally, studies reported different methodologies and experimental designs to evaluate novel cardioprotective interventions. Collectively, our findings highlight the need for better use of comprehensive practical guidelines for the conduct and analysis of experimental models of myocardial ischaemia and infarction.

To facilitate translation of preclinical findings into clinical applications, it is important to use models that adequately reflect clinical circumstances. For example, Lecour *et al*. recommend that the MI model should comprise both acute myocardial ischaemia and reperfusion rather than permanent occlusion.^91^ Among the 84 preclinical studies included in our review, 55 (accounting for 65% of studies) were conducted in the non-reperfused MI setting, therefore potentially limiting the clinical applicability of the findings. The majority of preclinical studies also focused on understanding molecular and cellular mechanisms of injury and protection, and our analysis shows that many of the proteins studied converge onto common signalling pathways and functional relationships. Although the findings from preclinical studies provide important mechanistic information, establishing that the efficacy of the interventions tested reflects the complex multifactorial interactions that modulate myocardial ischaemia *in vivo* is critical to identify the proteins with the most potential for clinical effectiveness.^88^ Failure to disseminate negative results, validate findings across multiple centres and animal models, or demonstrate the relevance of a therapeutic target in humans are other barriers to successfully translate experimental interventions into clinical therapies.^88,89,91^

Ultimately, the premature assessment of cardioprotective therapy in the clinical setting may be partly responsible for the failure of protein-based therapies in patients with MI.^88^ The IMPACT guidelines recommend that validation of preclinical findings is first achieved by demonstrating reduction in infarct size (and possibly also coronary microvascular obstruction) and reproducibility of findings in at least three centres in small animal *in vivo* ischaemia–reperfusion injury models in the first instance, followed by validation and reproducibility in large animal models.^91^ The majority of studies included in our review were conducted in the non-reperfused setting, and very few studies were in large animals. Additionally, most studies used either male or female animals, without validating findings in both sexes. Although a desirable rather than a required IMPACT criteria, assessment of infarct size and LV remodelling was performed at least 28 days post-infarction in most but not all small animal studies in our analysis, and only up to 8 weeks in large animal studies, instead of the recommended 3 months.^91^ Information related to assessment of outcomes in animals with a comorbidity that mimic the real-world conditions of patients with acute MI was sparse. For potential protein-based therapies to successfully translate from preclinical into clinical studies, experimental models of ischaemia–reperfusion injury should reflect the clinical population, include models with relevant comorbidities, and use both sexes and a range of ages.

### 3.10 Translation of cardioprotective interventions into clinical practice

Of the protein-based therapies with the highest potential cardioprotective benefits identified in our ranking framework analysis, EPO^77–79^ and G-CSF^83–85,95^ have been extensively investigated in late clinical development in the STEMI setting but have failed to progress into approved therapies for use in patients. Acute MI is a complex disease presenting many challenges that can hinder the successful development of cardioprotective therapies. Key challenges identified in the translation of experimental findings into the clinical setting are summarized in *Table 1*.

**Table 1:** Challenges in translating preclinical findings of protein-based therapies into the clinical STEMI setting and recommendations for future development

Challenges	Recommendations
Experimental data limited to small animal models of acute MI (such as mice, rats, and rabbits); limited testing in clinically relevant large animal MI models of MIRI	Investigate only therapies that have shown robust and consistent cardioprotection in experimental studies^89^ Follow practical guidelines for assessing cardioprotective efficacy of protein-based therapies in a rigorous and reproducible manner^90,91^
Experimental studies testing protein-based therapies use mostly healthy animals, mimicking myocardial ischaemia by mechanical injury of the coronary artery, which is not representative of the aetiology or complexity of the disease (potential confounding factors)	Integrate *in vitro* and *in vivo* approaches (incorporate advanced *in vitro* models, validate findings across both *in vitro* and *in vivo* models)^90,91^ Select preclinical models that reflect the clinical population, including models with relevant comorbidities, use of both sexes and a range of ages^91^
Doses used in clinical trials are often substantially lower than those used in animal studies (e.g. EPO^79^)	Conduct dose-dependent experiments as part of a full characterization of the compound of interest,^96^ to establish the therapeutic dose range and pharmacokinetic/pharmacodynamic relationship of the candidate protein, as well as the range of exposure that is considered pharmacodynamically relevant^97^ Consider Phase 2 studies to optimize the most effective dose before testing for clinical efficacy^89^
Complications related to acute ischaemia/reperfusion injury are relatively rare, leading to low recruitment, event rates, discontinuation of clinical trials, and associated lack of statistical power to assess potential benefits of treatment^88^	Identify patient subgroups most likely to benefit from therapies, enabling the recruitment of relatively small patient numbers in adequately powered studies^95^ In a *post hoc* analysis of the STEM-AMI OUTCOME study, subgroups of patients with low bone marrow cell mobilization or with severe LV systolic dysfunction benefited most from G-CSF treatment^95^
Clinical trials (e.g. REVIVAL 3) are often not powered to evaluate long-term clinical outcomes, such as survival.^79^ Reduction in infarct size has not translated into improved clinical outcomes	Use relevant endpoints for cardioprotection; align preclinical endpoints with clinical outcomes, assess functional, structural, and molecular outcomes, and validate and use clinically translatable biomarkers

EPO, erythropoietin; G-CSF, granulocyte colony-stimulating factor; LV, left ventricular; MI, myocardial infarction; MIRI, myocardial ischaemia/reperfusion injury; STEMI, ST-segment elevation MI.

Of those proteins that were clinically tested in more than one trial, EPO and G-CSF are both known to modulate the JAK/STAT and PI3K/Akt pathways (albeit to varying degrees and in different cell types); it is important to highlight that the failure of those protein therapies in clinical development does not invalidate the potential relevance of their downstream pathways. The development of current and future protein-based therapies should instead focus on evidence-based harmonized protocols, robust experimental rigour, and alignment of preclinical models with real-world clinical scenarios to overcome the persistent translational barrier in AMI research.

## 4. Discussion

**Figure 3:**
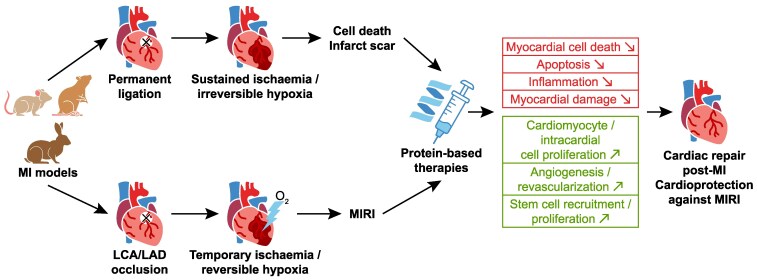
Potential mechanisms of action for the effects of protein-based therapies on cardiac repair post-MI and cardioprotection against MIRI, based on the MI model studies included in this review. LAD, left anterior descending artery; LCA, left coronary artery; MI, myocardial infarction; MIRI, myocardial ischaemia/reperfusion injury.

The physiological characteristics of post-MI cardiac repair and MIRI are complex and involve multiple, interacting signalling pathways with a myriad of key mediators participating in cellular responses of survival, repair, and regeneration.^25^ Our current assessment of the literature reflects the large number of protein-based therapies investigated in preclinical models of ischaemia and/or MIRI with multiple pleiotropic mechanisms, supporting the concept that some proteins applied early after acute MI may promote lasting improvements in heart function (*Figure 3*). Interestingly, most proteins studied in the various preclinical MI models with or without reperfusion exerted beneficial effects through multiple mechanisms; these included promoting infarct healing through increased angiogenesis/revascularization, reduced myocardial cell death, and reduced inflammation when administered post-MI, and reducing infarct size and/or fibrosis through reduced inflammation, increased angiogenesis, and reduced myocardial cell death and apoptosis when administered following reperfusion. Overall, the potential for protein-based therapies to be a viable therapeutic option post-MI is due to their ability to target multiple signalling pathways that are involved in the crosstalk among cardiomyocytes, fibroblasts, immune cells, and vascular and lymphatic endothelial cells.^66,98^

Despite the many studies in this review that demonstrated promising efficacy with protein-based therapies in small animal models, there has been, thus far, limited translation of the potential cardioprotective effects of protein-based therapies from preclinical models to humans. In order for potential therapeutics to move from basic research to reaching patients, important considerations are to be made at each stage of the clinical and translational research process.^99,100^ To demonstrate the cardioprotective effect of an intervention that also potentially correlates with other clinical outcomes in humans, studies must therefore be designed carefully, and the correct choice of clinical endpoint is critical.^101^ In our analysis, most preclinical studies had incomplete assessment of endpoints, with only select endpoints reported; notably, safety and pharmacokinetics/pharmacodynamics information was generally absent. It is indeed a limitation if only selected efficacy outcomes are reported, potentially leading to a reporting bias.^102^ Myocardial infarct size is often a key endpoint of clinical trials,^103,104^ and potential viable surrogate endpoints include assessment of myocardial infarct size and myocardial salvage index for assessing cardioprotection.^105,106^ However, many trials targeting MIRI have failed to show a clear clinical benefit of therapeutic interventions, demonstrated by primary endpoints that were not met, including reduction in infarct size.^101,107^ Clinical endpoints should explore death, morbidity, imaging, or electrocardiographic evidence of MI, and composite endpoints with standardized definitions of each to allow for comparability across different clinical trials.^103,106,108^ Imaging endpoints are of particular relevance as similar methodologies used in large animals can be applied to patients in clinical investigations and improve the transferability and translational impact of experimental results.^103^

The majority of studies we reviewed started administration of protein-based therapies immediately post-MI; however, only some studies investigated the effect of timing of dose on the improvement of LV function, which is an important consideration in the translational assessment of therapeutics.^109^ In these few studies, a delay in administration of protein therapy of up to 48 h seemed to have little impact on improved LV function. In a dog model, a 6-h or 1-week delay of EPO administration did not reduce infarct size.^57^ In a small animal model of MI without reperfusion, an 8-week delay was only associated with improved systolic function at the highest dose level of GGF2 administered.^47^ In humans, there was no impact on LV function or infarct size vs. placebo with EPO-α administration within 4 h of successful primary or rescue PCI.^77^ EPO-β administered immediately, 24 and 48 h after successful PCI (with primary PCI undergone within 24 h from symptom onset) did not improve LVEF or reduce infarct size.^78^ It should be noted that protein-based therapies were typically administered immediately after reperfusion in the preclinical studies included in this review, much earlier than what is achieved in clinical trials or a real-world setting. These studies suggest the existence of a therapeutic time window following reperfusion where protein-based therapies are most effective; beyond this window, the protein-induced protective effects may be reduced or even abolished. Additionally, only very few studies included in this review have thoroughly assessed dose-dependent effects by employing several doses of the therapeutic agent, with reported observed effects that were dose-dependent.^46,110–113^ Assessing the optimal timing, dose, and frequency of administration of each individual protein therapy and linking the effect to the exposure of the drug is crucial to determine the optimal effect on LV function and cardiac repair and, ultimately, enable the comparison of different therapeutic strategies across different studies.

In patients with acute MI, time remains an important predictor of outcome, with time from symptom onset to reperfusion being one of the main determining factors in clinical outcomes, and especially functional independence measured as 90-day disability outcomes, which are largely independent of final infarct size.^114^ In practice, time from symptom onset to reperfusion has been reported to average up to 6 h,^115^ beyond which myocardial salvage and functional recovery start to decline.^116^ Therapies that could exert cardioprotective effects and impact on patient outcomes, even with delayed administration, are, therefore, desirable.

**Figure 4:**
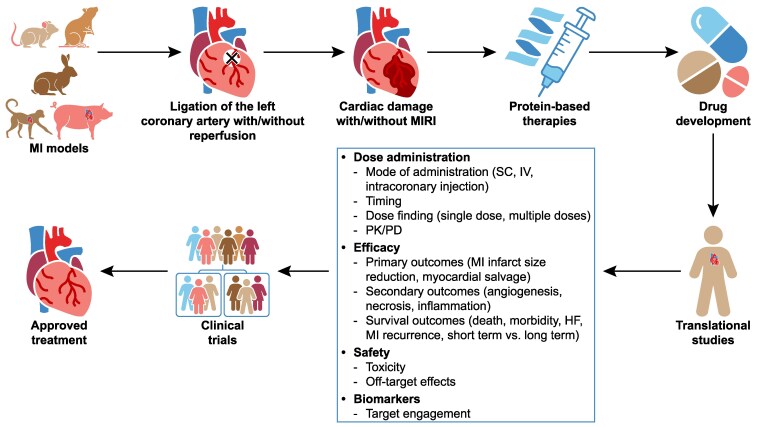
Overview of preclinical MI models in research and translational studies for the development of protein-based therapies in the STEMI setting. HF, heart failure; IV, intravenous; MI, myocardial infarction; MIRI, myocardial ischaemia/reperfusion injury; PD, pharmacodynamics; PK, pharmacokinetics; SC, subcutaneous.

Safety assessments should also be reported to ensure that any unwanted adverse events do not arise from treatment, and, given that protein therapies often have pleiotropic effects, it is important to understand the mechanisms of action and therapeutic targets of these proteins. However, none of the preclinical studies used target engagement biomarkers, leaving uncertainty about whether the tested protein truly engaged its target and modulated the intended pathway. This information is critical for guiding administration route, determining the dose needed for full target engagement, and linking the drug to physiological effects, all key steps in predicting clinical efficacy. A small number of protein-based therapies, including recombinant proteins, hormones, growth factors, and peptides, have been investigated in clinical trials, with most of these targeting multiple signalling pathways involved in cardiac repair and cardiac function improvement.^66^ Many targets for cardiac repair and potential cardioprotection against MIRI have been identified and validated in preclinical models, with promising preliminary data reported. Further research is warranted to evaluate the potential therapeutic application of protein-based therapy on cardiac structure and function improvements in patients with STEMI (*Figure 4*).

Findings from this literature review should be interpreted in the context of its limitations. Many of the studies included in our analysis did not report information relating to randomization, allocation concealment, random housing (measures used, if any, to house the animals in a random manner), blinding, or random outcome assessment, constituting a major limitation in reviewing the literature of experimental animal studies.^117^ Studies included in our analysis largely present positive results, and there is a lack of reporting of neutral and negative results; this may reflect a significant publication bias towards positive outcomes. Additionally, Skyschally *et al*.^118^ have shown that many published preclinical cardioprotection studies lacked statistical power, contributing to a lack of robustness, which may also be a major limitation of the literature included in our analysis. No head-to-head comparisons between different protein-based therapies had been conducted; hence, protein therapies with potential therapeutic advantages over other protein therapies could not be identified. Similarly, several studies lacked a positive control. Furthermore, only 9 of the 84 studies presented large animal data, whereas the remaining data came from studies conducted in small animals, including mice, rats, and rabbits. Small animals used in the studies included in our analysis generally had homogeneous characteristics and lacked information regarding age or comorbidities that would be representative of a typical patient. The majority of studies involved permanent ligation as models of MI, which is not representative of reperfusion treatment conducted in clinical practice; additionally, studies of ischaemia/reperfusion in small animal models of MI cannot distinguish whether the response is to the initial injury (ischaemia) or injury following reperfusion, thereby limiting the relevance for translation into humans.^119^ Given the pathophysiological difference of MIRI between small animals and humans, preliminary results in small animal models should be confirmed in large animal models, such as pigs or dogs, prior to translation into studies of humans.^120^

This systematic literature review comprehensively describes modified proteins and protein-based therapies with a potential therapeutic use to limit damage and/or mediate cardiac repair in the STEMI setting. Further research is required for many of these therapies, as part of the translational process, to establish their efficacy and safety profile in humans, and to lower the risk of HF after MI by reducing I/R injury and/or improving infarct repair. Additionally, logistics of administration of each therapy should be assessed in future studies to ensure that the optimal treatment effect is achieved through harnessing the therapeutic potential of proteins involved in the post-MI processes.

## Supplementary Material

cvag100_Supplementary_Data

## Data Availability

The data underlying this article are available in the article and in its online supplementary material.
