# Supplementary material for: Proteins targeting ischaemic-reperfusion injury and repair after myocardial infarction: a systematic literature review
Source: Cardiovasc Res. 2026 May 7;122(9):1143–58. doi: 10.1093/cvr/cvag100 (PMC13307559; doi:10.1093/cvr/cvag100)
Supplement: cvag100_Supplementary_Data [file cvag100_supplementary_data.docx]

**Proteins targeting ischaemic-reperfusion injury and repair after myocardial infarction: a systematic literature review**

Asparuh Gardev^a^, Derek J Hausenloy^b–e^, Anton Pekcec^a^

^a^ Boehringer Ingelheim Pharma GmbH & Co. KG, Ingelheim am Rhein, Germany (AG: [asparuh.gardev@boehringer-ingelheim.com](mailto:asparuh.gardev@boehringer-ingelheim.com); AP: [anton.pekcec@boehringer-ingelheim.com](mailto:anton.pekcec@boehringer-ingelheim.com))

^b^ Cardiovascular & Metabolic Disorders Program, Duke-National University of Singapore Medical School, Singapore

^c^ National Heart Research Institute Singapore, National Heart Centre, Singapore

^d^ Yong Loo Lin School of Medicine, National University Singapore, Singapore

^e^ The Hatter Cardiovascular Institute, University College London, London, UK

**Methods for the protein ranking framework**

A protein ranking framework was developed to assess the efficacy of protein-based therapies across preclinical studies and to compare protein candidates according to their cardioprotective potential. A ranking approach was used by first assigning scores to each of the proteins tested in the individual preclinical studies, based on the reported outcomes. Improvement in cardiac function (improvement in left ventricular ejection fraction and/or fractional shortening) was considered the most clinically relevant and consistently reported outcome and defined as the primary outcome. Infarct size reduction and/or fibrosis reduction were considered as secondary outcomes and predictors of long-term cardiac function. Tertiary outcomes included any cellular and/or molecular improvement reported such as angiogenesis, apoptosis reduction, inflammation reduction, cardiomyocyte proliferation, cell recruitment and proliferation, and capillary density. Finally, survival was used as a quaternary outcome. A score of 4 was given if the primary outcome was reported (i.e., if a significant benefit of the protein-based therapy over control was reported); followed by a score of 3, 2, and 1 if the secondary, tertiary, and quaternary outcomes were reported. A score of 0 was given if no change in the considered outcomes was reported, and a minus 1 score was assigned to a protein with a worse outcome compared with its relevant control. Aggregate scores were averaged across protein-based therapies (in cases where proteins were investigated in more than one study). A translational weighting was then given to protein-based therapies, considering efficacy that was demonstrated across multiple studies, models (MI with or without reperfusion), or species (small or large animals). Proteins studied across two studies, 3–4 studies, 5–6 studies, and 7 studies or more were assigned a score of 1, 2, 3, and 4, respectively. Proteins studied across two different models of MI were given a score of 1, and a score of 2 if studied across three models. These weighting scores were added to the averaged main outcome score. An aggregate score was used to rank the protein-based therapies with highest potential efficacy according to the most meaningful ranking parameters and weighting factors previously defined (**Table S12**).

**Table S1.** Search strategy overview

| Database/source | Search window | #Results |
| --- | --- | --- |
| Embase | 02/06/2018 – 02/06/2023 | 1,421 |
| MEDLINE | 02/06/2018 – 02/06/2023 | 1,230 |
| ClinicalTrials.gov | Primary completion on or after 02/06/2023 | 300 |
| EudraCT | Trial status: Ongoing, Restarted, Temporarily Halted, Trial now transitioned | 611 |
| Citation searching | N/A | 173 |
| Total number of records | – | 3,735 |
| Total number of records without duplicates | – | 2,879 |

**Table S2.** Original Cochrane search

|  | Search |  |
| --- | --- | --- |
| Filters | #1 | MeSH descriptor: [Myocardial Infarction] explode all trees |
|  | #2 | (“myocardial infarction”):ti,ab,kw |
|  | #3 | #1 or #2 |

**Table S3.** Original MEDLINE search ran on 2 June 2023

|  | Search component | Search string |
| --- | --- | --- |
| Disease | #1 | (("Myocardial Infarction"[Majr]) OR ("myocardial infarction"[Title]) OR ("heart infarction"[Title]) OR ("cardiac infarction"[Title])) |
| Key focus | #2 | **AND** (("inflammatory"[Title/Abstract ]) OR ("inflammation"[Title/Abstract]) OR ("inflammatories"[Title/Abstract]) OR ("antiinflammatory"[Title/Abstract]) OR ("Inflammation"[Mesh:NoExp]) OR ("immune"[Title/Abstract]) OR ("immunity"[Title/Abstract]) OR ("antigen-presenting cells"[Mesh:NoExp]) OR ("proliferative"[Title/Abstract]) OR ("proliferation"[Title/Abstract]) OR ("cell proliferation"[Mesh:NoExp]) OR ("reparative"[Title/Abstract]) OR ("repair"[Title/Abstract]) OR ("apoptosis"[Title/Abstract]) OR ("apoptotic"[Title/Abstract]) OR ("cell death"[Title/Abstract]) OR ("apoptosis"[Mesh:NoExp]) OR ("angiogenesis"[Title/Abstract]) OR ("angiogenic"[Title/Abstract]) OR ("antiangiogenic"[Title/Abstract]) OR ("angiogenesis modulating agents"[Mesh:NoExp]) OR ("fibrosis"[Title/Abstract]) OR ("fibrosis"[Mesh:NoExp]) OR ("fibrotic"[Title/Abstract]) OR ("antifibrotic"[Title/Abstract]) OR ("fibrogenic"[Title/Abstract]) OR ("reperfusion"[Title/Abstract]) OR ("reperfusion injury"[Mesh:NoExp]) OR ("myocardial reperfusion injury"[Mesh:NoExp]) OR ("remodel"[Title/Abstract]) OR ("remodeling"[Title/Abstract]) OR ("remodelling"[Title/Abstract]) OR ("ventricular remodeling"[Mesh:NoExp]) OR ("healing"[Title/Abstract]) OR ("heal"[Title/Abstract]) OR ("endothelial cell"[Title/Abstract]) OR ("Endothelial Cells"[Mesh:NoExp]) OR ("fibroblast"[Title/Abstract]) OR ("Fibroblasts"[Mesh:NoExp]) OR ("cardiomyocyte"[Title/Abstract]) OR ("myocyte"[Title/Abstract]) OR ("Myocytes, Cardiac"[Mesh:NoExp])) |
| Proteins | #3 | **AND** (("Blood Proteins"[Mesh]) OR ("blood protein"[Title/Abstract]) OR ("plasma protein"[Title/Abstract]) OR ("Carrier Proteins"[Mesh]) OR ("carrier protein"[Title/Abstract]) OR ("binding protein"[Title/Abstract]) OR ("Cystatins"[Mesh]) OR ("cystatin"[Title/Abstract]) OR ("Intercellular Signaling Peptides and Proteins"[Mesh]) OR ("intercellular signaling peptide"[Title/Abstract]) OR ("intercellular signalling peptide"[Title/Abstract]) OR ("intercellular signaling protein"[Title/Abstract]) OR ("intercellular signalling protein"[Title/Abstract]) OR ("cytokine"[Title/Abstract]) OR ("growth factor"[Title/Abstract]) OR ("endothelin derivative"[Title/Abstract]) OR ("Glycoproteins"[Mesh]) OR ("glycoprotein"[Title/Abstract]) OR ("Golgi Matrix Proteins"[Mesh]) OR ("golgi matrix"[Title/Abstract]) OR ("Nerve Tissue Proteins"[Mesh]) OR ("nerve tissue protein"[Title/Abstract]) OR ("Thymosin"[Mesh]) OR ("thymosin") OR ("Matrikine") OR ("Recombinant Proteins"[Mesh]) OR ("recombinant protein"[Title/Abstract]) OR ("angiogenic proteins"[Mesh:NoExp]) OR ("angiogenic protein"[Title/Abstract]) OR ("angiogenesis modulator"[Title/Abstract])) |
| Studies/ experiments | #4 | **AND** (("animal experimentation"[Mesh:NoExp]) OR ("Models, Animal"[Mesh:NoExp]) OR ("model"[Title/Abstract]) OR ("animal"[Title/Abstract]) OR ("Mice"[Mesh:NoExp]) OR ("mice"[Title/Abstract]) OR ("mouse"[Title/Abstract]) OR ("nonhuman"[Title/Abstract]) OR ("Primary Cell Culture"[Mesh:NoExp]) OR ("culture"[Title/Abstract]) OR ("Humans"[Mesh:NoExp]) OR ("human"[Title/Abstract]) OR ("Rats"[Mesh:NoExp]) OR ("rat"[Title/Abstract]) OR ("Rodentia"[Mesh:NoExp]) OR ("rodent"[Title/Abstract]) OR ("Organ Culture Techniques"[Mesh:NoExp]) OR ("in vivo"[Title/Abstract]) OR ("In Vitro Techniques"[Mesh:NoExp]) OR ("in vitro"[Title/Abstract]) OR ("recombinant"[Title/Abstract]) OR ("controlled study"[Title/Abstract])) |
| Therapy | #5 | **AND** (("therapy") OR ("therapeutic") OR ("treatment") OR ("treat") OR ("therapeutically") OR ("therapies") OR ("Therapeutics"[Mesh:NoExp]) OR ("Drug Therapy"[Mesh:NoExp])) |
| Exclusions | #6 | **NOT** (("Address"[Publication type]) OR ("address"[Title/Abstract]) OR ("Autobiography"[Publication type]) OR ("autobiography"[Title/Abstract]) OR ("Bibliography"[Publication type]) OR ("bibliography"[Title/Abstract]) OR ("Biography"[Publication type]) OR ("biography"[Title/Abstract]) OR ("Case Reports"[Publication type]) OR ("case report"[Title/Abstract]) OR ("report a case"[Title/Abstract]) OR ("Clinical Conference"[Publication type]) OR ("clinical conference"[Title/Abstract]) OR ("conference"[Title/Abstract]) OR ("Clinical Trial Protocol"[Publication type]) OR ("clinical trial protocol"[Title]) OR ("clinical protocol"[Title]) OR ("Clinical research protocol"[Title]) OR ("Comment"[Publication type]) OR ("Comment"[Title]) OR ("Congress"[Publication type]) OR ("congress"[Title/Abstract]) OR ("Consensus Development Conference"[Publication type]) OR ("consensus development conference"[Title/Abstract]) OR ("Consensus Development Conference, NIH"[Publication type]) OR ("Dataset"[Publication type]) OR ("dataset"[Title]) OR ("Dictionary"[Publication type]) OR ("dictionary"[Title/Abstract]) OR ("Directory"[Publication type]) OR ("Directory"[Title/Abstract]) OR ("Duplicate Publication"[Publication type]) OR ("duplicate publication"[Title/Abstract]) OR ("Editorial"[Publication type]) OR ("editorial"[Title/Abstract]) OR ("English Abstract"[Publication Type]) OR ("English abstract"[Title/Abstract]) OR ("Evaluation study"[Publication type]) OR ("Evaluation study"[Title/Abstract]) OR ("Fetschrift"[Publication type]) OR ("Fetschrift"[Title/Abstract]) OR ("Government Publication"[Publication type]) OR ("government publication"[Title/Abstract]) OR ("Guideline"[Publication type]) OR ("guideline"[Title/Abstract]) OR ("guidelines"[Title/Abstract]) OR ("Historical Article"[Publication type]) OR ("historical article"[Title/Abstract]) OR ("Interactive Tutorial"[Publication type]) OR ("interactive tutorial"[Title/Abstract]) OR ("interview"[Publication type]) OR ("Interview"[Title/Abstract]) OR ("survey"[Title/Abstract]) OR ("questionnaire"[Title/Abstract] OR ("Introductory Journal Article"[Publication type]) OR ("introductory journal article"[Title/Abstract]) OR ("Lecture"[Publication type]) OR ("lecture"[Title/Abstract]) OR ("Legal Case"[Publication type]) OR ("legal case"[Title/abstract]) OR ("Legislation"[Publication type]) OR ("legislation"[Title/Abstract]) OR ("Letter"[Publication type]) OR ("letter"[Title/Abstract]) OR ("News"[Publication type]) OR ("news"[Title/Abstract]) OR ("Newspaper Article"[Publication type]) OR ("newspaper article"[Title/Abstract]) OR ("Observational Study"[Publication type]) OR ("observational study"[Title/Abstract]) OR ("Observational Study, Veterinary"[Publication type]) OR ("observation study"[Title/Abstract]) OR ("Overall"[Publication type]) OR ("overall"[Title]) OR ("Patient Education Handout"[Publication type]) OR ("patient education handout"[Title/Abstract]) OR ("Periodical Index"[Publication type]) OR ("periodical index"[Title/Abstract]) OR ("Personal Narrative"[Publication type]) OR ("personal narrative"[Title/Abstract]) OR ("Portrait"[Publication type]) OR ("portrait"[Title/Abstract]) OR ("Practice Guideline"[Publication type]) OR ("practice guideline"[Title/Abstract]) OR ("pragmatic clinical trial"[Publication type]) OR ("pragmatic clinical trial"[Title/Abstract]) OR ("Retracted publication"[Publication type]) OR ("retracted publication"[Title/Abstract]) OR ("Retraction of Publication"[Publication type]) OR ("retraction of publication"[Title/Abstract]) OR ("Scientific Integrity Review"[Publication type]) OR ("Scientific Integrity Review"[Title/Abstract]) OR ("Technical Report"[Publication type]) OR ("technical report"[Title/Abstract]) OR ("Twin study"[Publication type]) OR ("twin study"[Title/Abstract]) OR ("Validation study"[Publication type]) OR ("Validation study"[Title/Abstract]) OR ("Video-Audio Media"[Publication type]) OR ("Video-Audio media"[Title/Abstract]) OR ("Webcast"[Publication type]) OR ("webcast"[Title/Abstract]) OR ("meta analysis"[Title/Abstract]) OR ("metaanalysis"[Title/Abstract]) OR ("meta-analysis"[Title/Abstract]) OR ("meta analyses"[Title/Abstract]) OR ("metaanalyses"[Title/Abstract]) OR ("meta-analyses"[Title/Abstract]) OR ("prospective study"[Title/Abstract]) OR ("cohort analysis"[Title/Abstract]) OR ("drug dose comparison"[Title/Abstract]) OR ("retrospective study"[Title/Abstract]) OR ("intermethod comparison"[Title/Abstract]) OR ("diagnostic test accuracy study"[Title/Abstract]) OR ("case control study"[Title/Abstract]) OR ("feasibility study"[Title/Abstract]) OR ("pilot study"[Title/Abstract]) OR ("outcomes research"[Title/Abstract]) OR ("cross sectional study"[Title/Abstract]) OR ("correlational study"[Title/Abstract]) OR ("computer model"[Title/Abstract]) OR ("evidence based medicine"[Title/Abstract]) OR ("evidence based practice"[Title/Abstract]) OR ("proportional hazards model"[Title/Abstract]) OR ("quality control"[Title/Abstract]) OR ("trend study"[Title/Abstract]) OR ("simulation"[Title/Abstract]) OR ("cardiosphere"[Title/Abstract]) OR ("drug dosage form comparison"[Title/Abstract]) OR ("longitudinal study"[Title/Abstract]) OR ("medical record review"[Title/Abstract]) OR ("methodology"[Title]) OR ("experimental design"[Title]) OR ("factorial design"[Title/Abstract]) OR ("mathematical model"[Title/Abstract]) OR ("multivariate logistic regression analysis"[Title/Abstract]) OR ("statistical model"[Title/Abstract]) OR ("study design"[Title]) OR ("cancer model"[Title/Abstract]) OR ("clinical audit"[Title/Abstract]) OR ("decapitation"[Title/Abstract]) OR ("dosage schedule comparison"[Title/Abstract]) OR ("feeder cell"[Title/Abstract]) OR ("genetic model"[Title/Abstract]) OR ("least absolute shrinkage and selection operator"[Title/Abstract]) OR ("least absolute shrinkage and selection operator"[Title/Abstract]) OR ("logistic regression analysis"[Title/Abstract]) OR ("partial least squares regression"[Title/Abstract]) OR ("population based case control study"[Title/Abstract]) OR ("total quality management"[Title/Abstract]))) |
| Filters | #7 | **Filters:** in the last 5 years, English |

**Table S4**. Original Embase search ran on 2 June 2023

|  | Search component | Search string |
| --- | --- | --- |
| Disease | #1 | ('heart infarction'/mj OR 'acute heart infarction'/mj OR 'heart infarction':ti OR 'cardiac infarction':ti) |
| Key focus | #2 | **AND** ('inflammatory':ab,ti OR 'inflammation':ab,ti OR 'inflammatories':ab,ti OR 'antiinflammatory':ab,ti OR 'inflammation'/de OR 'immune':ab,ti OR 'immunity':ab,ti OR 'immunocompetent cell'/de OR 'proliferative':ab,ti OR 'proliferation':ab,ti OR 'cell proliferation'/de OR 'reparative':ab,ti OR 'repair':ab,ti OR 'apoptosis':ab,ti OR 'apoptotic':ab,ti OR 'cell death':ab,ti OR 'apoptosis'/de OR 'angiogenesis':ab,ti OR 'angiogenic':ab,ti OR 'antiangiogenic':ab,ti OR 'angiogenesis'/de OR 'fibrosis':ab,ti OR 'fibrotic':ab,ti OR 'antifibrotic':ab,ti OR 'fibrogenic':ab,ti OR 'fibrosis'/de OR 'reperfusion':ab,ti OR 'reperfusion injury'/de OR ‘myocardial ischemia reperfusion injury’/de OR 'remodel':ab,ti OR 'remodelling':ab,ti OR 'remodeling':ab,ti OR 'heart ventricle remodeling'/de OR 'healing':ab,ti OR 'heal':ab,ti OR 'tissue repair'/de OR 'endothelial cell':ab,ti OR ‘endothelium cell’/de OR 'fibroblast':ab,ti OR ‘fibroblast’/de OR 'cardiomyocyte':ab,ti OR ‘cardiac muscle cell’/de OR 'myocyte':ab,ti) |
| Proteins | #3 | **AND** ('plasma protein'/exp OR ‘plasma protein’:ab,ti OR ‘blood protein’:ab,ti OR 'carrier proteins and binding proteins'/exp OR ‘carrier protein’:ab,ti OR ‘binding protein’:ab,ti OR 'cystatin'/exp OR ‘cystatin’:ab,ti OR 'cystatin b'/exp OR 'cystatin c'/exp OR 'cystatin m'/exp OR 'cystatin s'/exp OR 'cytokine'/exp OR ‘cytokine’:ab,ti OR 'growth factor'/exp OR ‘growth factor’:ab,ti OR 'endothelin derivative'/exp OR ‘endothelin derivative’:ab,ti OR ‘intercellular signaling peptide’:ab,ti OR ‘intercellular signalling peptide’:ab,ti OR ‘intercellular signaling protein’:ab,ti OR ‘intercellular signalling protein’:ab,ti OR 'glycoprotein'/exp OR ‘glycoprotein’:ab,ti OR 'golgi matrix'/exp OR ‘golgi matrix’:ab,ti OR ‘nerve tissue protein’:ab,ti OR 'thymosin'/de OR ‘thymosin’:ab,ti OR 'matrikine' OR 'recombinant protein'/exp OR ‘recombinant protein’:ab,ti OR 'angiogenesis modulator'/de OR ‘angiogenesis modulator’:ab,ti OR ‘angiogenic protein’/de OR ‘angiogenic protein’:ab,ti) |
| Studies/ experiments | #4 | **AND** ('animal experiment'/de OR 'animal model'/de OR 'model':ab,ti OR 'animal':ab,ti OR 'mouse'/de OR 'mouse':ab,ti OR ‘mice’:ab,ti OR 'animal cell'/de OR 'nonhuman'/de OR 'nonhuman':ab,ti OR 'controlled study'/de OR 'animal tissue'/de OR 'cell culture'/de OR 'culture':ab,ti OR 'human cell'/de OR 'human tissue'/de OR 'human'/de OR 'human':ab,ti OR 'rat'/de OR 'rat':ab,ti OR 'rodent'/de OR 'rodent':ab,ti OR 'in vivo study’/de OR 'in vivo':ab,ti OR 'in vitro study'/de OR 'in vitro':ab,ti OR 'recombinant':ab,ti) |
| Therapy | #5 | **AND** ('therapy' OR 'therapeutic' OR 'treatment' OR 'treat' OR 'therapeutically' OR 'therapies' OR 'therapy'/de OR 'drug therapy'/de) |
| Exclusions | #6 | **NOT** ('prospective study':ab,ti OR 'prospective study'/de OR 'cohort analysis':ab,ti OR 'cohort analysis'/de OR 'drug dose comparison':ab,ti OR 'drug dose comparison'/de OR 'retrospective study':ab,ti OR 'retrospective study'/de OR 'case report':ab,ti OR 'report a case':ab,ti OR 'case report'/de OR 'intermethod comparison':ab,ti OR 'intermethod comparison'/de OR 'observational study':ab,ti OR 'observation study':ab,ti OR 'observational study'/de OR 'diagnostic test accuracy study':ab,ti OR 'diagnostic test accuracy study'/de OR 'case control study':ab,ti OR 'case control study'/de OR 'feasibility study':ab,ti OR 'feasibility study'/de OR 'pilot study':ab,ti OR 'pilot study'/de OR 'outcomes research':ab,ti OR 'outcomes research'/de OR 'clinical protocol':ti OR ‘clinical trial protocol’:ti OR 'clinical research protocol':ti OR 'clinical protocol'/de OR 'stem cell culture'/de OR 'cross sectional study':ab,ti OR 'cross sectional study'/de OR 'correlational study':ab,ti OR 'correlational study'/de OR 'computer model':ab,ti OR 'computer model'/de OR 'guideline':ab,ti OR 'guidelines':ab,ti OR 'practice guidelines'/de OR ‘practice guideline’:ab,ti OR 'evidence based medicine':ab,ti OR 'evidence based medicine'/de OR 'proportional hazards model':ab,ti OR 'proportional hazards model'/de OR 'quality control':ab,ti OR 'quality control'/de OR 'trend study':ab,ti OR 'trend study'/de OR 'meta analysis':ab,ti OR 'metaanalysis':ab,ti OR 'meta-analysis':ab,ti OR 'meta analyses':ab,ti OR 'metaanalyses':ab,ti OR 'meta-analyses':ab,ti OR 'meta analysis'/de OR 'meta analysis topic'/de OR 'questionnaire':ab,ti OR 'survey':ab,ti OR 'questionnaire'/de OR 'simulation':ab,ti OR 'simulation'/de OR 'bone marrow culture'/de OR 'cardiosphere':ab,ti OR 'cardiosphere'/de OR 'drug dosage form comparison':ab,ti OR 'drug dosage form comparison'/de OR 'longitudinal study':ab,ti OR 'longitudinal study'/de OR 'medical record review':ab,ti OR 'medical record review'/de OR 'methodology':ti OR 'methodology'/de OR 'experimental design':ti OR 'experimental design'/de OR 'factorial design':ab,ti OR 'factorial design'/de OR 'mathematical model':ab,ti OR 'mathematical model'/de OR 'multivariate logistic regression analysis':ab,ti OR 'multivariate logistic regression analysis'/de OR 'statistical model':ab,ti OR 'statistical model'/de OR 'study design':ti OR 'study design'/de OR 'cancer model':ab,ti OR 'cancer model'/de OR 'clinical audit':ab,ti OR 'clinical audit'/de OR 'decapitation':ab,ti OR 'decapitation'/de OR 'dosage schedule comparison':ab,ti OR 'dosage schedule comparison'/de OR 'evidence based practice':ab,ti OR 'evidence based practice'/de OR 'feeder cell':ab,ti OR 'feeder cell'/de OR 'genetic model':ab,ti OR 'genetic model'/de OR 'interview':ab,ti OR 'interview'/de OR 'least absolute shrinkage and selection operator':ab,ti OR 'least absolute shrinkage and selection operator'/de OR 'logistic regression analysis':ab,ti OR 'logistic regression analysis'/de OR 'partial least squares regression':ab,ti OR 'partial least squares regression'/de OR 'population based case control study':ab,ti OR 'population based case control study'/de OR 'structured interview'/de OR 'telephone interview'/de OR 'total quality management':ab,ti OR 'total quality management'/de OR 'comment':ti OR 'letter':ab,ti OR 'technical report':ab,ti OR 'editorial':ab,ti OR 'legislation':ab,ti OR ‘address’:ab,ti OR ‘autobiography’:ab,ti OR ‘bibliography’:ab,ti OR ‘biography’:ab,ti OR ‘conference’:ab,ti or ‘congress’:ab,ti OR ‘consensus development conference’:ab,ti OR ‘dataset’:ti OR ‘dictionary’:ab,ti OR ‘directory':ab,ti OR ‘duplicate publication’:ab,ti OR ‘English abstract’:ab,ti OR ‘evaluation study’:ab,ti OR ‘Fetschrift’:ab,ti OR ‘government publication’:ab,ti OR ‘historical article’:ab,ti OR ‘interactive tutorial’:ab,ti or ‘introductory journal article’:ab,ti OR ‘lecture’:ab,ti OR ‘legal case’:ab,ti OR ‘news’:ab,ti OR ‘newspaper article’:ab,ti OR ‘overall’:ti OR ‘patient education handout’:ab,ti OR ‘periodical index’:ab,ti OR ‘personal narrative’:ab,ti OR ‘portrait’:ab,ti OR ‘pragmatic clinical trial’:ab,ti OR ‘retracted publication’:ab,ti OR ‘retraction of publication’:ab,ti OR ‘scientific integrity review’:ab,ti OR ‘twin study’:ab,ti OR ‘validation study’:ab,ti OR ‘Video-Audio media’:ab,ti OR ‘webcast’:ab,ti) |
| Filters | #7 | **AND** ([article]/lim OR [article in press]/lim) AND [english]/lim AND [2018-2023]/py |

**Table S5.** Original ClinicalTrials.gov search

|  | Search |  |
| --- | --- | --- |
| Filters | #1 | Recruiting, Not yet recruiting, Active, not recruiting,  Enrolling by invitation, Unknown status Studies \|  Interventional Studies \| **Myocardial Infarction** \| Drug \|  Primary completion on or after 06/02/2023 |
|  | #2 | Recruiting, Not yet recruiting, Active, not recruiting,  Enrolling by invitation, Unknown status Studies \|  Interventional Studies \| **Myocardial Reperfusion Injury** \| Drug \| Primary completion on or after 06/02/2023 |
|  | #3 | Recruiting, Not yet recruiting, Active, not recruiting,  Enrolling by invitation, Unknown status Studies \|  Interventional Studies \| **Myocardial Injury** \| Drug \|  Primary completion on or after 06/02/2023 |
|  | #4 | Recruiting, Not yet recruiting, Active, not recruiting, Enrolling by invitation, Unknown status Studies \| Interventional Studies \| **Acute Coronary Syndrome** \| Drug \| Primary completion on or before 06/02/2023 |

**Table S6.** Original EudraCT search

|  | Search |  |
| --- | --- | --- |
| Filters | #1 | **Myocardial infarction**. Trial status: Ongoing, Restarted, Temporarily Halted, Trial now transitioned |
|  | #2 | **Myocardial reperfusion injury**. Trial status: Ongoing, Restarted, Temporarily Halted, Trial now transitioned |
|  | #3 | **Myocardial injury**. Trial status: Ongoing, Restarted, Temporarily Halted, Trial now transitioned |
|  | #4 | **Acute coronary syndrome**. Trial status: Ongoing, Restarted, Temporarily Halted, Trial now transitioned |

**Table S7.** Exclusion criteria at screening used for the selection of studies included in the review

| Exclusion criteria | Reason |
| --- | --- |
| “wrong article type” | being a non-primary paper (e.g. reviews, letters/commentaries and guidelines) |
| “non-English language” | being written in a language other than English |
| “wrong therapy area” | treatment not specific to MI or use of a disease model that does not adequately simulate MI e.g. ameroid constrictor models, or isoproterenol/doxorubicin-induced cardiac damage) |
| “non-blinded trial” | being an unblinded clinical trial |
| “no defined clinical trial” | detailing clinical experiments that are not part of a defined clinical trial |
| “source of protein unclear” | the source of the protein not being described (e.g. purchase or derivation) |
| “insufficient or unclear methodology or reporting of results” | employing methodology that is insufficient to address the research question, or unclear methodology or reporting of results |
| “in vitro or ex vivo experiments” | involving *in vitro* or *ex vivo* experiments |
| “no therapeutic application” | there being no therapeutic application |
| “no therapeutic benefit” | treatment demonstrating no or unconvincing therapeutic benefit |
| “no or non-standard functional outcomes” | there being no reported outcomes related to cardiac function, or assessing non-standard outcomes related to cardiac function |
| “treatment was not in the early stages post-MI” | therapy first being applied either pre-MI or after myocardial infarct resolution (e.g. >3 weeks post-MI in rodents)^1^ |
| “wrong protein type” | protein-based therapy involving non-endogenous human proteins |
| “synthetic treatment” | investigating synthetic therapies such as artificial proteins (e.g. monoclonal antibodies) other than modified proteins made to facilitate production, improve pharmacokinetics parameters, and/or enhance safety |
| “combination protein-based therapy” | therapy involving more than one protein |
| “therapy is not (solely) protein-based” | therapy not involving proteins or therapy involving proteins in combination with other non-protein-based therapies or products |
| “stem cells” | therapy involving stem cells |
| “gene-based therapy” | therapy involving gene therapy, genetic knock-down or overexpression |
| “thrombolytic therapy” | therapy involving thrombolytics |

MI, myocardial infarction.

**Table S8.** Records meeting the eligibility criteria for inclusion in the review.

| # | Identifier | Title |
| --- | --- | --- |
| 1 | **Achilli F, 2019** | G-CSF for extensive STEMI. Results from the STEMI-AMI OUTCOME CMR substudy |
| 2 | **Adini A, 2021** | The prominin-1-derived peptide improves cardiac function following ischemia |
| 3 | **An W, 2019** | Exogenous IL-19 attenuates acute ischaemic injury and improves survival in male mice with myocardial infarction |
| 4 | **Atar D, 2009** | Effect of intravenous FX06 as an adjunct to primary percutaneous coronary intervention for acute ST-segment elevation myocardial infarction – Results of the F.I.R.E. (efficacy of FX06 in the prevention of myocardial reperfusion injury) trial |
| 5 | **Azizi Y, 2015** | Post-infarct treatment with [Pyr^1^]apelin-13 improves myocardial function by increasing neovascularization and overexpression of angiogenic growth factors in rats |
| 6 | **Baehr A, 2020** | Agrin promotes coordinated therapeutic processes leading to improved cardiac repair in pigs |
| 7 | **Barlow SC, 2017** | Intracoronary delivery of recombinant TIMP-3 after myocardial infarction: effects on myocardial remodeling and function |
| 8 | **Benjanuwattra J, 2022** | The temporal impact of erythropoietin administration on mitochondrial function and dynamics in cardiac ischemia/reperfusion injury |
| 9 | **Beohar N, 2007** | Granulocyte-colony stimulating factor administration after myocardial infarction in a porcine ischemia-reperfusion model: functional and pathological effects of dose timing |
| 10 | **Bersell K, 2009** | Neuregulin1/ErbB4 signaling induces cardiomyocyte proliferation and repair of heart injury |
| 11 | **Bi W, 2021** | Neurotrophin-3 contributes to benefits of human embryonic stem cell-derived cardiovascular progenitor cells against reperfused myocardial infarction |
| 12 | **Birnbaum Y, 2022** | Recombinant apyrase (AZD3366) against myocardial reperfusion injury |
| 13 | **Bock-Marquette I, 2004** | Thymosin β4 activates integrin-linked kinase and promotes cardiac cell migration, survival and cardiac repair |
| 14 | **Chen H, 2019** | Ghrelin attenuates myocardial fibrosis after acute myocardial infarction via inhibiting endothelial-to mesenchymal transition in rat model |
| 15 | **Chintalgattu V, 2018** | Utility of glycosylated TIMP3 molecules: Inhibition of MMPs and TACE to improve cardiac function in rat myocardial infarct model |
| 16 | **Cho DI, 2019** | Antiinflammatory activity of ANGPTL4 facilitates macrophage polarization to induce cardiac repair |
| 17 | **Fang J, 2020** | Recombinant extracellular domain (p75ECD) of the neurotrophin receptor p75 attenuates myocardial ischemia-reperfusion injury by inhibiting the p-JNK/caspase-3 signaling pathway in rat microvascular pericytes |
| 18 | **Ferraro B, 2019** | Pro-angiogenic macrophage phenotype to promote myocardial repair |
| 19 | **Fu J, 2021** | Gastrin exerts a protective effect against myocardial infarction via promoting angiogenesis |
| 20 | **Gäbel R, 2009** | Single high-dose intramyocardial administration of erythropoietin promotes early intracardiac proliferation, proves safety and restores cardiac performance after myocardial infarction in rats |
| 21 | **Gao X-M, 2019** | Relaxin mitigates microvascular damage and inflammation following cardiac ischemia-reperfusion |
| 22 | **Grimm D, 1998** | Differential effects of growth hormone on cardiomyocyte and extracellular matrix protein remodeling following experimental myocardial infarction |
| 23 | **Harada M, 2005** | G-CSF prevents cardiac remodeling after myocardial infarction by activating the Jak-Stat pathway in cardiomyocytes |
| 24 | **Heinen A, 2019** | IGF1 treatment improves cardiac remodeling after infarction by targeting myeloid cells |
| 25 | **Heywood SE, 2017** | High-density lipoprotein delivered after myocardial infarction increases cardiac glucose uptake and function in mice |
| 26 | **Hill MF, 2013** | Intravenous glial growth factor 2 (GGF2) isoform of neuregulin-1β improves left ventricular function, gene and protein expression in rats after myocardial infarction |
| 27 | **Hirata A, 2006** | Erythropoietin enhances neovascularization of ischemic myocardium and improves left ventricular dysfunction after myocardial infarction in dogs |
| 28 | **Huang C-X, 2009** | Ghrelin inhibits post-infarct myocardial remodeling and improves cardiac function through anti-inflammation effect |
| 29 | **Hume RD, 2023** | PDGF-AB reduces myofibroblast differentiation without increasing proliferation after myocardial infarction |
| 30 | **Ji M, 2022** | Vaspin ameliorates cardiac remodeling by suppressing phosphoinositide 3-kinase/protein kinase B pathway to improve oxidative stress in heart failure rats |
| 31 | **Jin Y, 2018** | Exogenous BMP-7 facilitates the recovery of cardiac function after acute myocardial infarction through counteracting TGF-β1 signaling pathway |
| 32 | **Kawachi K, 2012** | Effects of erythropoietin on angiogenesis after myocardial infarction in porcine |
| 33 | **Klopsch C, 2009** | Intracardiac injection of erythropoietin induces stem cell recruitment and improves cardiac functions in a rat myocardial infarction model |
| 34 | **Klopsch C, 2018** | Intramyocardial angiogenetic stem cells and epicardial erythropoietin save the acute ischemic heart |
| 35 | **Klotz L, 2015** | Cardiac lymphatics are heterogeneous in origin and respond to injury |
| 36 | **Korf-Klingebiel M, 2015** | Myeloid-derived growth factor (C19orf10) mediates cardiac repair following myocardial infarction |
| 37 | **Krishnamurthy P, 2009** | IL-10 inhibits inflammation and attenuates left ventricular remodeling after myocardial infarction via activation of STAT3 and suppression of HuR |
| 38 | **Li G-H, 2016** | Dual effects of VEGF-B on activating cardiomyocytes and cardiac stem cells to protect the heart against short and long-term ischemia-reperfusion injury |
| 39 | **Li J, 2021** | Alarin alleviated cardiac fibrosis via attenuating oxidative stress in heart failure rats |
| 40 | **Liao Q, 2019** | Irisin exerts a therapeutic effect against myocardial infarction via promoting angiogenesis |
| 41 | **Lipšic E, 2004** | Timing of erythropoietin treatment for cardioprotection in ischemia/reperfusion |
| 42 | **Liu X, 2006** | Neuregulin-1/erbB-activation improves cardiac function and survival in models of ischemic, dilated, and viral cardiomyopathy |
| 43 | **Lobb DC, 2020** | Targeted injection of a truncated form of tissue inhibitor of metalloproteinase 3 alters post-myocardial infarction remodeling |
| 44 | **Lörchner H, 2021** | Concomitant activation of OSM and LIF receptor by a dual-specific hlOSM variant confers cardioprotection after myocardial infarction in mice |
| 45 | **Mauro AG, 2017** | A preclinical translational study of the cardioprotective effects of plasma-derived alpha-1 anti-trypsin in acute myocardial infarction |
| 46 | **Minatoguchi S, 2004** | Acceleration of the healing process and myocardial regeneration may be important as a mechanism of improvement of cardiac function and remodeling by postinfarction granulocyte colony-stimulating factor treatment |
| 47 | **Moon C, 2003** | Erythropoietin reduces myocardial infarction and left ventricular functional decline after coronary artery ligation in rats |
| 48 | **Moon C, 2005** | Cardioprotection by recombinant human erythropoietin following acute experimental myocardial infarction: dose response and therapeutic window |
| 49 | **Ohtsuka M, 2004** | Cytokine therapy prevents left ventricular remodeling and dysfunction after myocardial infarction through neovascularization |
| 50 | **O'Sullivan JF, 2011** | Potent long-term cardioprotective effects of single low-dose insulin-like growth factor-1 treatment postmyocardial infarction |
| 51 | **Pan Y, 2020** | Apela improves cardiac and renal function in mice with acute myocardial infarction |
| 52 | **Parry TJ, 2017** | Effects of neuregulin GGF2 (cimaglermin-α) dose and treatment frequency on left ventricular function in rats following myocardial infarction |
| 53 | **Prunier F, 2007** | Delayed erythropoietin therapy reduces post-MI cardiac remodeling only at a dose that mobilizes endothelial progenitor cells |
| 54 | **Qin Y-Y, 2022** | Neuropeptide Y attenuates cardiac remodeling and deterioration of function following myocardial infarction |
| 55 | **Reboll MR, 2017** | EMC10 (endoplasmic reticulum membrane protein complex subunit 10) is a bone marrow-derived angiogenic growth factor promoting tissue repair after myocardial infarction |
| 56 | **Reboll MR, 2022** | Meteorin-like promotes heart repair through endothelial KIT receptor tyrosine kinase |
| 57 | **Rotem I, 2022** | Osteopontin promotes infarct repair |
| 58 | **Serebryakova L, 2019** | Galanin and its N-terminal fragments reduce acute myocardial infarction in rats |
| 59 | **Soeki T, 2008** | Ghrelin suppresses cardiac sympathetic activity and prevents early left ventricular remodeling in rats with myocardial infarction |
| 60 | **Sugiyama A, 2020** | Long-term administration of recombinant canstatin prevents adverse cardiac remodeling after myocardial infarction |
| 61 | **Tang T-T, 2018** | Liver-heart crosstalk controls IL-22 activity in cardiac protection after myocardial infarction |
| 62 | **Tao J, 2011** | Apelin-13 protects the heart against ischemia-reperfusion injury through inhibition of ER-dependent apoptotic pathways in a time-dependent fashion |
| 63 | **Thavapalachandran S, 2020** | Platelet-derived growth factor-AB improves scar mechanics and vascularity after myocardial infarction |
| 64 | **Toldo S, 2011** | Alpha-1 antitrypsin inhibits caspase-1 and protects from acute myocardial ischemia-reperfusion injury |
| 65 | **Valle Raleigh J, 2017** | Reperfusion therapy with recombinant human relaxin-2 (serelaxin) attenuates myocardial infarct size and NLRP3 inflammasome following ischemia/reperfusion injury via eNOS-dependent mechanism |
| 66 | **van der Meer P, 2005** | Erythropoietin induces neovascularization and improves cardiac function in rats with heart failure after myocardial infarction |
| 67 | **Wang Z, 2018** | Irisin protects heart against ischemia-reperfusion injury through a SOD2-dependent mitochondria mechanism |
| 68 | **Wang C, 2020** | Cholecystokinin octapeptide reduces myocardial fibrosis and improves cardiac remodeling in post myocardial infarction rats |
| 69 | **Wang Y, 2020** | Mydgf promotes cardiomyocyte proliferation and neonatal heart regeneration |
| 70 | **Wang R, 2021** | Gastrin mediates cardioprotection through angiogenesis after myocardial infarction by activating the HIF-1α/VEGF signalling pathway |
| 71 | **Wang K, 2022** | Cardioprotection of klotho against myocardial infarction-induced heart failure through inducing autophagy |
| 72 | **Wang K, 2023** | Klotho improves cardiac fibrosis, inflammatory cytokines, ferroptosis, and oxidative stress in mice with myocardial infarction |
| 73 | **Wo D, 2016** | Opposing roles of Wnt inhibitors IGFBP-4 and Dkk1 in cardiac ischemia by differential targeting of LRP5/6 and β-catenin |
| 74 | **Wu B, 2014** | Interleukin-37 ameliorates myocardial ischaemia/reperfusion injury in mice |
| 75 | **Xi Y, 2019** | Recombinant Fc-Elabela fusion protein has extended plasma half-life and mitigates post-infarct heart dysfunction in rats |
| 76 | **Xu Z, 2020** | Human recombinant apyrase therapy protects against myocardial ischemia/reperfusion injury and preserves left ventricular systolic function in rats, as evaluated by 7T cardiovascular magnetic resonance imaging |
| 77 | **Xu J-Y, 2022** | Interleukin-5-induced eosinophil population improves cardiac function after myocardial infarction |
| 78 | **Zacharowski K, 2007** | The effects of the fibrin-derived peptide Bβ_15-42_ in acute and chronic rodent models of myocardial ischemia-reperfusion |
| 79 | **Zhang X, 2015** | OSM enhances angiogenesis and improves cardiac function after myocardial infarction |
| 80 | **Zhang X, 2016** | Apelin-13 protects against myocardial infarction-induced myocardial fibrosis |
| 81 | **Zhang NK, 2016** | Activation of endogenous cardiac stem cells by apelin-13 in infarcted rat heart |
| 82 | **Zhong S, 2020** | Apelin-13 alleviated cardiac fibrosis via inhibiting the PI3K/Akt pathway to attenuate oxidative stress in rats with myocardial infarction-induced heart failure |
| 83 | **Zhu R, 2016** | Interleukin-37 and dendritic cells treated with interleukin-37 plus troponin I ameliorate cardiac remodeling after myocardial infarction |
| 84 | **Zuo L, 2023** | Erythropoietin promotes myocardial infarction repair in mice by improving the function of Sca-1^+^ stem cells |
| 85 | **NCT05723315** | Effect of rhBNP on CMD in patients with STEMI after PPCI |
| 86 | **NCT04241601** | Low-dose interleukin-2 for the reduction of vascular inflammation in acute coronary syndromes – IVORY |

**Table S9.** *In vivo* small animal models of ischaemia/non-reperfusion (n = 52) investigating protein-based therapies that demonstrate an ability to limit damage and/or mediate cardiac repair when administered post-MI

| **Proteins, in alphabetical order** | **Administration method, timeline (when/for how long administered after MI), dosage(s)** | **Histomorphological endpoints with timepoint(s) when assessed after MI** | **Functional outcome, method (e.g. echo, MRI) with timepoints when assessed after MI** | **Other outcomes** | **Reference for protein therapy study** |
| --- | --- | --- | --- | --- | --- |
| **Agrin** Receptor Dag1, on cardiomyocytes | Intramyocardial injection of 50 μL at 1 μg per mouse recombinant rat agrin or saline (control) post-MI.  (Female mice; N varied per experiment) | Periinfarct vasculature by endomucin immunofluorescence 21 days post MI:  Infarct size **↘**  Vessel density in infarct **↗** | ND | Apoptosis in infarct areas 24 h post MI:  Myocardial cell death (TUNEL⁺) **↘**  Immunofluorescence of infarct/border areas 2 days post MI:  Myocardial cells (HSP60⁺) **↗** | Baehr A et al. (2020); doi.org/10.1161/CIRCULATIONAHA.119.045116^2^ |
| **Alarin**  Galaninergic system | I.p. injection of alarin (1.0 nM/kg/day in 300 µL saline) or saline (control) administered 1-day post-MI for 28 days.  (Male Sprague-Dawley rats; N = 8–10 per experiment) | Histology from heart sections at 30 days post MI (alarin vs control):  Fibrosis **↘** | ECG and haemodynamic monitoring (28- and 30-days post MI):  LVEF **↗**  Fractional shortening **↗**  +d*P*/dt_max_ **↗**  LVSP **↗**  LVEDP **↘**  LVVS **↘**  LVVD **↘**  LVESD **↘**  LVEDD **↘** | Collagen I, collagen III, and TGF-*β* expression in cardiac fibroblasts (30 days post MI, alarin vs control):  collagen I **↘**  collagen III **↘**  TGF-*β* **↘** | Li J et al. (2021); doi.org/10.1007/s00726-021-03005-8^3^ |
| **Alpha-1-antitrypsin (AAT)**  Caspase-1 | I.p. injection of 60 mg/kg AAT or albumin (control) immediately after AMI and then daily for 7 days.  (Male outbred ICR [CD1] mice; N = 6–21 per group) | Histology from heart transverse slides at 7 days after AMI:  Infarct size **~** | Transthoracic ECG at 7 days after AMI (prior to sacrifice):  LVEF **↗**  LVEDD **↘**  LVESD **↘** | Caspase-1 activity in heart homogenates (72 h after non-reperfused AMI):  Caspase-1 **↘** | Toldo S et al. (2011); do.org/10.1016/j.yjmcc.2011.05.003^4^ |
| **Angiopoietin-like 4 (ANGPTL4)**  Anti-inflammatory macrophages | I.p. injection of recombinant ANGPTL4 (1.5 mL; 200 ng) or vehicle (control) at day 0 and 1 after inducing MI.  (Male inbred Balb/C nude mice; N = 3–16 per group)  (Male inbred BALB/c nude mice; N varied per experiment) | ND | ECG at 14 days after MI (ANGPTL4 vs vehicle):  LVEF **↗**  Fractional shortening **↗**  IVSd **~**  IVSs **↗**  LVIDd **~**  LVIDs **↘**  LVPWd **↗**  LVPWs **↗** | Cardiac macrophage phenotype in heart tissue at 14 days after MI (ANGPTL4 vs vehicle):  CD206-expressing macrophages **↗** | Cho Di et al. (2019); doi.org/10.1172/jci.insight.125437^5^ |
| **Annexin A1 (AnxA1)**  Receptor FPR2, Rac1, NOX1 | I.p. injection of 10 µg human recombinant AnxA1 daily, up to 6 days post MI.  (Wild-type female C57BL/6 mice; N varied per experiment) | Ischaemic myocardium post MI (AnxA1 vs control):  Infarct size **↘** | Transthoracic ECG at 6 days post MI (AnxA1 vs control):  Stroke volume **↗**  LVEF **↗**  LV contractility **↗** | Angiogenesis in infarcted hearts 6 days post-MI (AnxA1 vs control):  Microvascular density **↗**  VEGF-A **↗** | Ferraro B et al. (2019); doi.org/10.1016/j.jacc.2019.03.503^6^ |
| **Apelin-13 / Apela**  Receptor APJ | Microdosing osmotic pump implanted subcutaneously continuously administered 1 mg/kg/day apela or saline (control) for 2 weeks post-MI.  (Male C57BL/6 mice; N = 5) | Histopathology at 4 weeks post MI (apela vs control):  Infarct size **↘**  Myocardial interstitial fibrosis **↘** | ECG at 2 and 4 weeks post MI (apela vs control):  LVEF **↗** | Myocardial neovascularisation (IHC) in infarcted heart 4 weeks post-MI (apela vs control):  CD31 cells **↗** | Pan Y et al. (2020); doi.org/10.1111/jcmm.15651^7^ |
| **Apelin-13 / Apela**  Receptor APJ, PI3K/akt, and NF-κB signalling pathways | I.p. injection of apelin-13 (200 µg/kg/day) for 4 weeks post-MI or saline (control).  (Male Sprague-Dawley rats; N = 6) | Histopathology post MI (apelin-13 vs control):  Fibrosis area **↘** | Physiology at 4 weeks post MI (apelin-13 vs control):  LVSP **↗**  +d*P*/dt_max_ **↗**  -d*P*/dt_max_ **↗**  LVEDP **↘** | Ang II levels and NF-κB activity in heart tissue at 4 weeks post MI (apelin-13 vs control):  Ang II **↘**  Relative NF-κB **↘** | Zhang X et al. (2016); doi.org/10.3892/mmr.2016.5163^8^ |
| **Apelin-13 / Apela**  Receptor APJ | Intramyocardial injection of 200 ng apelin (in 20 µL saline) or saline (control) at four infarct border zone sites during MI.  (Male Sprague Dawley rats; N = 10) | Histology of infarcted and LV areas at 28 days post MI (apelin-13 vs control):  Infarct size ↘ | Transthoracic ECG prior MI and at 28 days post MI (apelin-13 vs control):  LVEF ↗  Fractional shortening ↗  LVEDV ↘  LVESV ↘ | Markers of endogenous cardiac stem or progenitor cells in the border zone and infarct zone of rat hearts at 28 days post MI (apelin-13 vs control):  c-kit ↗  Flk-1 ↗  Sca-1 ↗ | Zhang NK et al. (2016); doi.org/10.3727/096368916X691123^9^ |
| **Apelin-13 / Apela**  Receptor APJ, PI3K/akt, and MAPK signalling pathways | I.p. injection of apelin-13 (10 nmol/kg/day) during MI or saline (control), with subsequent daily administrations for 28 days post-MI.  (Male Sprague-Dawley rats; N = 8) | Histology of infarcted heart and LV areas assessed by Masson’s trichrome staining at 28 days post MI (apelin-13 vs control):  Fibrosis **↘** | Haemodynamic monitoring via conductance at 28 days post MI (apelin-13 vs control):  LVSP **↗**  LVEDP **↘**  +d*P*/dtmax **↗**  Transthoracic ECG at 28 days post MI (apelin-13 vs control):  LVVs **↘**  LVVd **↘**  LVEDD **↘**  LVESD **↘**  LVEF **↗**  Fractional shortening **↗** | Collagen I, collagen III, and TGF-*β* expression in hearts (28 days post MI, apelin-13 vs control):  collagen I **↘**  collagen III **↘**  TGF-*β* **↘** | Zhong S et al. (2020); doi.org/10.1042/BSR20200040^10^ |
| **Bone morphogenetic protein-7 (BMP-7)** TGF-*β*1 signalling pathway | I.p injection of 5 µg/kg recombinant murine BMP-7 daily or saline (control) for 2 weeks post-MI. (Male Sprague-Dawley rats; N = 20 per group) | Heart tissue 2 weeks post MI with TTC or Masson staining (BMP-7 vs saline):  Infarct size **↘**  Interstitial fibrosis **↘**  Heart weight/body weight ratio **↘** | ECG of LV after 2 weeks post MI (BMP-7 vs saline):  LVEF **↗**  Fractional shortening **↗**  LVEDD **↘**  LVESD **↘** | Overall survival after 2 weeks post MI (BMP-7 vs saline):  Mortality **~** | Jin Y et al. (2018); doi.org/10.1620/tjem.244.1^11^ |
| **Canstatin** TGF-*β*1 signalling pathway | I.p. injection of recombinant mouse canstatin (20 µg/kg) or vehicle (control) daily for 28 days from MI.  (Male Wistar rats; N varied per experiment) | Cardiomyocyte hypertrophy and fibrosis at 28 days post-MI:  Scar thickness **↗**  Cardiomyocyte hypertrophy **↘**  Interstitial fibrosis **↘** | ECG at 3, 7, 14, 21, and 28 days post MI:  LVEF **↗**  Fractional shortening **↗**  LVIDs **↘**  LVIDd **~** | Overall survival after 28 days post MI (Canstatin vs control):  Mortality **↘** | Sugiyama A et al. (2020); doi.org/10.1038/s41598-020-69736-y^12^ |
| **Cholecystokinin 8 (CCK-8)** PI3K/Akt signalling pathway, TGF-*β*1, CTGF | I.p. injection of 50 µg/kg CCK-8 daily for 4 weeks following MI ligation.  (Male Sprague-Dawley rats; N = 60) | Fibrosis 4 weeks post-MI:  Fibrosis **↘**  Disorganised cardiomyocyte structure **↘** | ECG at 2- and 4-weeks post-MI:  LVEF **↗**  Fractional shortening **↗**  (At 4 weeks post-MI)  LVIDs **↘** LVIDd **↘**  (At 4 weeks post-MI) | Plasma BNP levels 4 weeks post-MI  Hypertrophic/HF marker(s) (BNP) **~**  Inflammation (TGFβ1) **↘**  Mortality **~** | Wang C et al. (2020); doi.org/10.1016/j.biocel.2020.105793^13^ |
| **Erythropoietin (EPO)**  TGF, TNF, IL-6 signalling pathways | Single i.p. injection of recombinant human EPO (3,000 units/kg in 0.3 ml of saline) or 0.3 ml of saline (control) <5 min post-MI.  (Male Sprague-Dawley rats; n = 8 for functional assessment and n = 6 for apoptosis assessment) | Infarct size 8 weeks post-MI:  Infarct size **↘** | ECG at week 8 post-MI:  LVEF **↗**  Fractional area change **↗**  Fractional shortening **↗**  LVESV **↘**  LVEDV **~**  LVEDA **↘**  LVEDD **↘**  LVESD **↘**  LVESA **~** | Haematocrit 2, 4, 7, 10, 14 and 21 days post-MI:  Haematocrit **~**  Apoptosis 24 hours post-MI:  Myocardial cell death **↘**  Mortality **~** | Moon C et al. (2003); doi.org/10.1073/pnas.1930406100^14^ |
| **Erythropoietin (EPO)**  Neovascularisation and haematopoietic effect | I.p. injection of 40 µg/kg recombinant human EPO administered in male Sprague-Dawley rats immediately post-MI (single early EPO bolus; n = 12), immediately post MI and once every 3 weeks for 9 weeks (multiple early EPO bolus; n = 13), or at 3 weeks post-MI and once every 3 weeks for 9 weeks (multiple late EPO bolus; n = 13) or MI only (control) | Infarct size at 9 weeks post-MI  Single early bolus of EPO vs control:  Infarct size **↘**  Multiple early EPO bolus vs control:  Infarct size **↘**  Multiple late EPO bolus vs control:  Infarct size **~** | Haemodynamic measurements by microtip pressure transducer at 9 weeks post-MI  Single early bolus of EPO vs control:  LVSP **~**  dLVP **~**  +d*P*/dt_max_ **~**  -d*P*/dt_max_ **~**  LVEDP **↘**  Multiple early EPO bolus vs control and multiple late EPO bolus vs control:  LVSP **↗**  dLVP **↗**  +d*P*/dt_max_ **↗**  -d*P*/dt_max_ **↗**  LVEDP **↘** | Capillary density at 9 weeks post-MI (vs control):  Capillary density **↗** (in all 3 EPO groups)  Capillary / cardiomyocyte ratio **↗** (in multiple early and multiple late EPO bolus groups only)  Survival in the first 24h post-MI:  Mortality **~** | van der Meer P et al. (2005); doi.org/10.1016/j.jacc.2005.03.044^15^ |
| **Erythropoietin (EPO)**  JAK2/STAT5, p38 MAPK, signalling pathways | I.c. injection of 30 µg/kg long-acting EPO analog or PBS (control) administered during MI.  (SPF male C57BL/6J mice; N = 6–8 per experimental group) | Infarct size at 7-, 14- and 28-days post-MI and myocardial mass at 14- and 28-days post-MI:  HW/BW **↘**  HW/TL **↘** (At 14 days post-MI)  Infarct size **↘**  LV area **↘** | ECG at 7-, 14-, and 28-days post-MI:  LVEF **↗**  Fractional shortening **↗**  (both at 7, 14, and 28 days post-MI) | Immunofluorescent staining of mouse myocardium at 2 weeks post-MI:  Microvessel density **↗**  TUNEL staining of mouse LV tissues at 7 days post-MI:  Myocardial cell death **↘**  Survival rate at 28 days post MI:  Mortality **↘** | Zuo L et al. (2023); doi.org/10.13294/j.aps.2023.0013^16^ |
| **Erythropoietin (EPO)**  Upregulation of cell-cycle proliferation genes | Four intramyocardial injections (25 μL each) of recombinant human EPO (3000 U/kg, Epoetin-α) dissolved in 0.9% saline immediately after MI, or saline alone (control).  (Male Lewis rats; N = 99 and N = 95) | Histopathology (HE staining) at 48 hours post-MI:  Area of acute ischaemic myocardial damage **~** | Pressure-volume loop with conductance catheterisation at 6 weeks post-MI:  LV cardiac output **↗**  MAP **↗**  (At 48h post-MI)  LVEDP **↘** | Haematological analyses at 24 hours, 48 hours, and 14 days post-MI:  Haematopoietic cell mobilisation **↗**  (At 24h and 48h post-MI)  Cell proliferation 24- and 48-hours post-MI:  Intracardiac cell proliferation (cyclin D1 gene at 24h) **↗**  Intracardiac cell proliferation (Ki-67^+^ at 48h) **↗**  Capillary density at 7 days post-MI:  Angiogenesis **↗**  Survival rate at 6 weeks post MI:  Therapy-related survival **~** | Gaebel R et al. (2009); doi.org/10.1510/icvts.2008.191916^17^ |
| **Erythropoietin (EPO)**  eNOS, Akt signalling pathways | Four intramyocardial injections (25 µL each) of recombinant human EPO at a total dosage of 3000 U/kg dissolved in 0.9% saline, or saline alone (control) given along the border of the blanched myocardium immediately after MI. (Male Lewis rats; N = 99) | LV transversed tissue sections (Sirius Red and Fast Green FCF staining) at 6 weeks post-MI:  Infarct size **↘**  Interventricular septum thickness **↘**  HW/BW **↘**  RVW/BW **↘**  Fibrosis **↘**  Cardiomyocyte size **↘**  Myocardial cell death **↘**  Myocardial damage (cTnT) **↘** (At 2 weeks)  Infarct wall thickness **~**  LVW/BW **~** | Cardiac function via pressure-volume loop catheterisation at 6 weeks post-MI:  LVEF **↗**  LV stroke work **↗**  LV stroke volume **↗**  LV +d*P*/dt_max_ **↗**  LV -d*P*/dt_max_ **↗**  LV relaxation time **↘**  RV Pmax **↘**  RVESP **↘**  HR **~**  LV Pmax **~**  LVEDV **~**  LVESV **~**  RV +d*P*/dt_max_ **~** | Haematological analyses 24 hours, 48 hours, and 2 weeks post-MI:  Capillary density **↗** (At 6 weeks)  Stem cell recruitment/mobilisation **↗** (At 24 hours)  Upregulation of tissue pro-survival signals (Akt, eNOS) **↗** (At 24 and 48 hours)  Haematocrit **↗** (At 48 hours) | Klopsch C et al. (2009); doi.org/10.1111/j.1582-4934.2008.00546.x^18^ |
| **Erythropoietin (EPO)**  TGF-*β*/WNT signalling pathway via Akt activation and FOS upregulation | 300 U/kg recombinant human EPO delivered immediately after MI by epicardial fibrin hydrogel patch (EPO-F) implant vs epicardial fibrin patch alone (control).  (Male Lewis rats; N = 29 per group) | LV transversed tissue sections (Sirius Red and Fast Green FCF staining) at 6 weeks post-MI:  Infarct size **↘**  Cardiomyocyte size **↘**  Fibrosis **↘**  Infarction zone wall thickness **↗** | Cardiac function via pressure-volume loop catheterisation at 6 weeks post-MI:  LVEF **↗**  +d*P*/dt_max_ **↗**  -d*P*/dt_max_ **↗**  LV Pmax **↗**  LVEDV **↘**  LVESV **↘**  HR **↗**  LVEDP **~**  LV stroke volume **~** | Cardiac mesenchymal cell proliferation and density at 24 hours post-MI:  Cardiac mesenchymal stem cells signalling factors **↗**  Cardiac mesenchymal cell proliferation and density **↗**  Inflammation ~  Reticulocyte count ~ | Klopsch C et al. (2018); doi.org/10.1242/dmm.033282^19^ |
| **Erythropoietin (EPO)**  PI3K signalling pathway | IV injection of 50, 150, 500, 1000, or 3000 IU/kg of recombinant human EPO or sterile water (control) administered immediately post-MI. Additionally, delayed doses of 150 and 3000 IU/kg given after 4, 8, 12, and 24 hours post MI.  (Male Sprague Dawley rats; N = 12 per group) | Heart transverse sections (HE and azan staining) at 4 weeks post-MI for all dose groups:  Infarct size **↘** (except for 50 IU/kg dose)  For the 3000 µg/kg dose with delayed administration:  Infarct size **↘** (after 12 hours of delay)  Infarct size **~** (after 4, 8, 24 hours of delay)  For the 150 µg/kg dose with delayed administration:  Infarct size **~** (after 4, 8, 12, 24 hours of delay) | Cardiac function via ECG at 4 weeks post-MI for all dose groups (immediate administration):  **150, 500, 1000 and 3000 IU/kg doses** (except for 50 IU/kg dose)**:**  LVEF **↗**  LVEDV **↘**  LVESV **↘**  Cardiac function via ECG at 4 weeks post-MI for 3000 IU/kg **after 4, 8, 12, and 24 hours of delay** post MI:  LVEF **↗** (except for 24 hours)  LVEDV **↘** (except for 8 and 24 hours)  LVESV **↘** (except for 8 and 24 hours) | Apoptotic cardiomyocytes in the area at risk 24 hours post-MI:  **150 and 3000 IU/kg doses:**  Apoptosis **↘**  **50 IU/kg doses:**  Apoptosis **~** | Moon C et al. (2005); doi.org/10.1007/s10557-005-3189-6^20^ |
| **Fc-Elabela fusion protein (Fc-ELA-21)** Apelin-APJ signalling pathway | S.c administration of 300 µg/kg Fc-ELA-21 immediately post-MI and daily for 4 weeks (or PBS as control) post-MI.  (Male Sprague-Dawley rats; N = 12) | Heart tissue samples (Masson’s trichrome staining) at 4 weeks post-MI:  Fibrosis **↘** | Haemodynamic measurements via pressure transducer and ventricular catheterisation at 4 weeks post-MI:  LVSP **↗**  +d*P*/dt_max_ **↗**  -d*P*/dt_max_ **↗**  HR **↘**  LVEDP **↘** | Immunofluorescence staining at 4 weeks post MI:  Angiogenesis **↗**  Cardiomyocyte proliferation **↗**  Myocardial cell death **↘** | Xi Y et al. (2019); doi.org/10.1016/j.ijcard.2019.04.089^21^ |
| **Gastrin** PI3K/Akt/VEGF pathway | S.c. infusion of gastrin at 120 µg/kg body weight/day, or saline (control) via minipump for 28 days post-MI.  (Male C57/BL6 mice; N varied per experiment) | Ventricular tissue sections (Masson’s trichrome staining) at 28 days post-MI (vs control):  Fibrosis **↘** | Cardiac function via ECG at 28 days post-MI (vs control):  LVEF **↗**  Fractional shortening **↗**  LVEDD **↘**  LVESD **↘** | Immunofluorescence staining at 14 days post-MI:  Angiogenesis **↗**;  TUNEL^+^ cardiomyocytes at 2 days post MI:  Myocardial cell death **↘**  Ki67 immunostaining at 28 days post MI:  Cardiomyocyte proliferation **~**  Mortality **↘** | Fu J et al. (2021); doi.org/10.1186/s10020-021-00352-w^22^ |
| **Gastrin** HIF-1α/VEGF pathway | I.p. injection of gastrin 30 µg/kg, or saline (control), twice daily for 2 weeks post-MI.  (Male C57BL/6J mice; N = 40) | Heart tissue sections (TTC and Masson’s trichrome staining) at 2- and 4-weeks post MI:  Infarct size **↘**  Fibrosis **↘** | Cardiac function via ECG at 2- and 4-weeks post MI:  LVEF **↗**  Fractional shortening **↗**  LVEDD **↘**  LVESD **↘** | Immunofluorescence staining at 1 month post MI:  Angiogenesis **↗**  Mortality **↘** (at 4 weeks post MI) | Wang R et al. (2021); doi.org/10.1038/s41598-021-95110-7^23^ |
| **Ghrelin** Receptor GHS-R, cardiac sympathetic nerve activity pathways | S.c injection of ghrelin (100 µg/kg, twice daily) or saline (control) administered for 2 weeks from the day after MI.  (Sprague-Dawley rats; N = 15) | Heart transverse tissue sections (Masson’s trichrome and Sirius Red staining) at 2 weeks post-MI:  Infarct size **~**  Fibrosis **↘**  HW/BW **↘**  Anterior wall thickness **~**  Posterior wall thickness **~** | Cardiac function via ECG and haemodynamic measurements via LV pressure and catheterisation at 2 weeks post-MI:  Fractional shortening **↗**  +d*P*/dt_max_ **↗**  -d*P*/dt_max_ **↗**  LVEDP **↘**  HR **↘**  LVSP **~**  MAP **~**  LVEDD **↘** |  | Soeki T et al. (2008); doi.org/10.1152/ajpheart.00643.2007^24^ |
| **Ghrelin** Receptor GHS-R, inflammatory, TNF-α, NF-kB signalling pathways | S.c injection of 100 µg/kg ghrelin (twice daily), or saline (control) 7 days post-MI for 4 weeks.  (Male Sprague-Dawley rats; n = 12 [control] or 15 [ghrelin]) | LV tissue sections (Masson’s trichrome staining) at 4 weeks post-treatment:  Infarct size **~**  Scar thickness **↗** | Cardiac function via ECG and haemodynamic measurements via LV catheterisation at 4 weeks post-treatment:  +d*P*/dt_max_ **↗**  Fractional shortening **↗**  LVEDP **↘**  HR **~**  LVESP **~**  LVEDD **↘**  LVESD **↘** | ELISA and RT-PCR at 4 weeks post treatment:  Markers of cardiac remodelling (MMP-2 and MMP-9) **↘**  Myocardial cell death **↘**  Inflammation **↘**  Mortality **~** | Huang C-X et al. (2009); doi.org/10.1016/j.peptides.2009.09.004^25^ |
| **Ghrelin** Disruption of TGF-*b*1/Smads signalling pathway in a GHSR-1a/AMPK-dependent manner | I.p. injection of synthetic rat ghrelin (10 or 100 µg/kg), or saline (control) twice a day for 8 weeks post-MI.  (Male Sprague-Dawley rats; n = 10 per dose group) | Heart sections (Masson’s trichrome and HE staining) at 8 weeks post-MI:  Fibrosis **↘**  HW/BW **↘** | Cardiac function via ECG at 8 weeks post-MI:  LVEF **↗**  Fractional shortening **↗**  E/A **↗**  LVEDV **↘** |  | Chen H et al. (2019); doi.org/10.1016/j.peptides.2018.09.001^26^ |
| **Glial growth factor 2 (GGF2)** Receptor ErbB, Akt pathway | **Daily dosing experiments:**  IV injection of GGF2 (two dose levels: 10 nmol/kg/dose, 0.65 mg/kg and 50 nmol/kg/dose, 3.25 mg/kg), or vehicle (control), daily from post-MI days 8–28  **Effect of dose frequency experiments:**  IV injection of GGF2 (50 nmol/kg/dose, 3.25 mg/kg) from post-MI days 8–28 once every 24, 48, or 96 hours  **Once a week versus every two weeks dosing experiments:**  IV dosing of GGF2 (2.6 mg/kg) either once a week for 6 weeks post-MI, once a week for 40 weeks post-MI, or once every 2 weeks for 40 weeks post-MI  **Various dose levels once every two weeks experiments:**  IV dosing of GGF2 (0.35, 0.875, 1.75 or 3.5 mg/kg) once every two weeks for 13 weeks post-MI  (Sprague Dawley rats; N varied per experiment) | **Daily dosing experiments –**  Heart tissue sections (HE and Masson’s trichrome staining) at 38 days post-MI:  Infarct size **~** | **Daily dosing and effect of dose frequency experiments –** Cardiac function via ECG at 10-day intervals through 38 days post-MI  **Daily dosing experiments:**  LVEF **↗** Fractional shortening **↗**  LVESV **↘**  (All doses at 18- and 28-days post-MI)  **Effect of dose frequency experiments:**  LVEF **↗**  Fractional shortening **↗**  LVESV **↘**  (All doses at 28- and 38-days post-MI)  **Once a week dosing experiments –** Cardiac function via ECG at 1-week intervals through 6 weeks post-MI or at 4-week intervals through 44 weeks post-MI:  LVEF **↗** Fractional shortening **↗**  LVESV **↘**  (At 1–40 weeks post-MI)  **Various dose levels once every two weeks experiments –** Cardiac function via ECG at 1-week intervals through 15 weeks post-MI:  LVEF **↗** Fractional shortening **↗**  (All dose levels)  LVESV **↘**  (Only 3.5 mg/kg dose) |  | Parry TJ et al. (2017); doi.org/10.1016/j.ejphar.2016.12.024^27^ |
| **Glial growth factor 2 (GGF2)** Receptor ErbB, pathway | IV injection of 0.625 mg/kg or 3.25 mg/kg recombinant GGF2 or vehicle (control) every second day starting at 1 (early treatment) or 8 (late treatment) weeks post-MI and continuing for 4 weeks.  (Male Sprague Dawley rats; n varied per group) | Heart tissue sections (Masson’s trichrome staining) at 5 weeks post-MI:  Infarct size **~**  HW/BW **~**  (both doses)  Fibrosis **~**  (both doses) | Cardiac function via ECG at two-week intervals until 5 weeks post-MI:  **Both doses, administered early post MI:**  Fractional shortening **↗**  (At 5 weeks post-MI)  LVIDd **~**  **High dose, administered late post MI:**  Fractional shortening **↗**  (At 10, 12, 14, 16 weeks post-MI) | Reactive carbonyl derivatives content in post-MI heart homogenates (low or high dose group):  Myocardial oxidative stress **~** | Hill MF et al. (2013); doi.org/10.1371/journal.pone.0055741^28^ |
| **Granulocyte colony-stimulating factor (G-CSF)** Jak-Stat signalling pathway | S.c. injection of G-CSF (10–100 µg/kg/day) or saline (control) for 5 days post-MI starting either immediately post-MI, 3 days post-MI, or 7 days post-MI (Male C57BL/6 mice; N varied per experiment) | Heart tissue sections (Masson’s trichrome staining) at 2 weeks post-MI:  Infarct size **↘** | Cardiac function via ECG and pressure transducer with catheterisation at 2 weeks post-MI:  Fractional shortening **↗**  (At 100 µg/kg/day dose and treatment started either immediately or 3 days post-MI)  +d*P*/dt **↗**  -d*P*/dt **↗**  LVEDP **↘**  (Treatment starting immediately post-MI)  LVEDD **↘**  (At 100 µg/kg/day dose and treatment starting immediately post-MI) | Immunohistochemistry staining at 2 weeks post-MI:  Angiogenesis **↗**  Myocardial cell death **↘**  Cardiomyocyte proliferation **~**  Mortality **↘**  Stem cell mobilisation **↗** | Harada M et al. (2005); doi.org/ doi.org/ 10.1038/nm1199^29^ |
| **Granulocyte colony-stimulating factor (G-CSF)** Akt/VEGF signalling pathway | S.c. injection of G-CSF (100 µg/kg/day) or vehicle (control) for 5 days following MI.  (Male C57BL/6 mice; N = varied per group) | Heart tissue sections (HE and Azan-Mallory staining) at 2 weeks post-MI:  LV free wall thickness **↗**  Infarct area wall thickness **↗**  Fibrosis **~** | Cardiac function via microtip transducer and cannulation at 2 weeks post-MI:  LVSP **↗**  LVEDP **↘**  +d*P*/dt_max_ **↗**  -d*P*/dt_max_ **↗**  HR **~** | Immunostaining and immunohistochemistry at 4 days post-MI:  Angiogenesis **↗**  Inflammation (granulocytes) **↗**  TUNEL assay at 1-, 4-, and 7-days post-MI:  Apoptosis **↘**  Mortality **↘** | Ohtsuka M et al. (2004); doi.org/10.1096/fj.03-0637fje^30^ |
| **Growth hormone (GH)** Undetermined pathway | S.c injection of GH (2 IU/kg/day) or vehicle (control) administered immediately post-MI for four weeks (Male Wistar rats). Rats were split into groups according to MI size: small (n = 19), moderate (n = 21) and large (n = 20). Only rats with moderate and large MI were selected for detailed investigation | LV tissue sections (Masson’s trichrome staining) at 28 days post-MI:  **Moderate and large MI:**  Infarct size **~**  Cardiomyocyte width **↗**  Fibrosis **↘**  **Large MI:**  LV/BW ↗ | Cardiac function via ECG and haemodynamic measurements via pressure transducer with catheterisation at 28 days post-MI:  **Moderate MI:**  LVEDP **~**  LV/BW **~**  LVEDD **~**  **Large MI:**  LVEDP **↘**  LVESD **↘**  LVEDD **↘**  **Moderate and large MI:**  Fractional shortening **↗**  Mean right arterial pressure **↘**  𝜏 **↘**  HR **~**  Peak LVSP **~**  MAP **~**  LVEDD/BW **~** | **All animals (prior to groupings):**  Mortality **~** | Grimm D et al. (1998); doi.org/10.1016/s0008-6363(98)00181-3^31^ |
| **Insulin like growth factor binding protein 4 (IGFBP-4) mutant (IGFBP-4/H95P)** Wnt/*b*-catenin signalling pathway | I.c. injection of purified IGFBP-4/H95P (dose unclear) at 3 sites in the ischaemic border of the infarcted myocardium immediately following MI.  (Male C57BL/6 mice; N = 16) | Heart and hindlimb sections (Masson’s trichrome and HE staining) and immunofluorescence (hypertrophic/HF markers) at 4 weeks post-MI:  Infarct size **↘**  Hypertrophic/HF marker(s) (ANP, BNP and skeletal α-actin) **↘** | Cardiac function via 2D ECG 2-, 4-, 6- and 20-weeks post-MI:  LVEF **↗**  LV wall movement **↗**  Fractional shortening **↗** | ND | Wo D et al. (2016); doi.org/10.1161/CIRCULATIONAHA.116.024441^32^ |
| **Interleukin-5 (IL-5)** IL-5 receptor on eosinophils | I.p. injection of recombinant mouse IL-5 (100 µg/kg) 20 min, 1 day and 2 days after MI.  (Male C57BL/6 mice; N varies per experiment) | Heart tissue sections (HE and Sirius Red staining) at 28 days post-MI:  Infarct size **↘**  Infarct area wall thickness **~** | Cardiac function via ECG at 28 days post-MI:  LVEF **↗**  Fractional shortening **↗**  LVEDV **↘**  LVESV **↘** | FACS analysis (eosinophil count), and immunohistochemistry and immunofluorescence at 1-, 3- and 7-days post-MI:  Eosinophil recruitment **↗**  Angiogenesis **↗** | Xu JY et al. (2022); doi.org/10.1093/cvr/cvab237^33^ |
| **Interleukin-10 (IL-10)** HuR, p38 MAPK, and STAT3 signalling pathways | S.c injection of 50 µg/kg mouse recombinant IL-10 on 0, 1-, 3-, 5-, and 7-days post-MI.  (C57BL/6J mice; N = 8) | Heart slices (Masson’s trichrome staining) at 28-days post-MI:  Infarct size **↘**  Infarct wall thickness **↗**  Fibrosis **↘** | Cardiac function via 2D ECG at 14- and 28-days post-MI:  LVEF **↗**  Fractional shortening **↗**  HR **~**  LVESD **↘**  LVEDD **↘** | Immunohistochemistry at 3 days post-MI:  Inflammation **↘**  TUNEL assay at 3- and 28-days post-MI:  Cardiac cell apoptosis **↘**  Immunohistochemistry at 28 days post-MI:  Angiogenesis **↗**  (border zone)  Smooth muscle cell hyperplasia **↘** | Krishnamurthy P et al. (2009); doi.org/10.1161/CIRCRESAHA.108.188243^34^ |
| **Interleukin-19 (IL-19)** HO-1; STAT3 | I.p. injection of 10 ng/g^-1^ murine recombinant IL-19 immediately post-MI. IL-19 injections were continued daily for varying timeframes (3 days, 7 days and survival). (Male C57BL/6J mice; N varied per experiment) | Hearts (Evans Blue and TTC staining) at 24 hours post-MI:  Infarct size **↘** | Cardiac function via ECG at 7 days post-MI:  LVEF **↗**  Fractional shortening **↗**  HR **~**  LVEDD **~**  LVESD **~** | TUNEL assay at 24 hours post-MI:  Myocardial cell death **↘**  ELISA at 3 days post-MI:  Inflammation **↘**  VEGF mouse ELISA at 7 days post-MI:  Angiogenesis **↗**  Mortality **↘** | An W et al. (2019); doi.org/10.1111/bph.14549 ^35^ |
| **Interleukin-22 (IL-22)** FGF21, STAT3 | S.c. injection of recombinant mouse IL-22 (100 µg/kg) daily for 7 days beginning immediately post-MI.  (Male C57BL/6J mice; N = 50) | Heart sections (Masson’s trichrome staining) at 28 days post-MI:  Infarct size ↘  Fibrosis **↘** | Cardiac function via ECG and haemodynamic measurements via micromanometer-tipped catheter at 28 days post-MI:  LVEDP **↘**  LVEF **↗**  Fractional shortening **↗**  +d*P*/dt_max_ **↗**  -d*P*/dt_min_ **↗**  LVEDD **↘**  LVESD **↘** | Mortality and angiogenesis 28 days post-MI:  Mortality **↘**  Angiogenesis **↗**  Flow cytometry at 3- and 7-days post-MI:  Inflammation **↘** | Tang TT et al. (2018); doi.org/10.7150/thno.24723 ^36^ |
| **Interleukin-37 (IL-37)** TLRs, proinflammatory cytokines | I.p. injection of 1 µg of recombinant human IL-37 diluted in 200 µL PBS or PBS alone (control) 15 min prior to MI or 24 hours after MI, twice per week up to 28 days post-MI.  (Male C57BL/6 mice; N = varied per experiment) | Heart tissue sections (TTC staining) at 1-day post-MI:  Infarct size **↘**  Heart tissue sections (Masson’s trichrome staining) at 28 days post-MI:  Fibrosis **↘** | Cardiac function via ECG at 7- and 28-days post-MI:  LVEF **↗** Fractional shortening **↗**  LVESD **↘**  LVEDD **↘**  (At day 28 post-MI) | RT-PCR at 3-, and 7-days post-MI:  Inflammation **↘**  TUNEL assay at 1- and 28-days post-MI:  Myocardial cell death **↘**  Survival 28 days post MI:  Mortality **↘** | Zhu R et al. (2016); doi.org/10.1161/  JAHA.116.004406^37^ |
| **Irisin** ERK pathway, SOD2 | I.p. injection of irisin (5 µg/g/day) for 2 weeks post-MI.  (Male C57/BL6 mice; N varied per experiment) | Transcardial TTC perfusion (infarct size) and heart tissue sections (Masson’s trichrome staining; fibrosis) at 28 days post-MI:  Infarct size **↘**  Fibrosis ↘ | Cardiac function via ECG at 7-, 14- and 28-days post-MI:  LVEF **↗**  Fractional shortening **↗**  (At 28 days post-MI)  LVEDD **↘**  LVESD **↘** | Cardiomyocyte apoptosis (TUNEL assay) at 2 days post-MI and angiogenesis (immunofluorescence) at 14 days post-MI:  Myocardial cell death **↘**  Non-cardiomyocyte cell death **↘**  Angiogenesis **↗**  Immunofluorescence at 28 days post-MI:  Cardiomyocyte proliferation **~** | Liao Q et al. (2019); doi.org/10.1038/s41401-019-0230-z^38^ |
| **Klotho** AMPK/mTOR pathway | I.p. injection of 20 µg/kg Klotho protein every other day for 28 days starting 1-week post-MI.  (Male C57BL/6 mice; N = 5) | LV sections (Masson’s trichrome staining) at 5 weeks post-MI:  Fibrosis **↘** | Cardiac function via ECG at 5 weeks post-MI:  LVEF **↗**  Fractional shortening **↗** | RT-PCR, Western blots, and immunofluorescence at 5 weeks post-MI:  Inflammation **↘**  Oxidative stress **↘**  Ferroptosis **↘**  Hypertrophic/HF marker(s) (ANP and BNP) **↘**  Survival at 5 weeks post-MI:  Mortality **~** | Wang K et al. (2023); doi.org/10.1007/s13105-023-00945-5^39^ |
| **Klotho** PI3K/AKT/mTOR pathway | I.p. injection of 20 µg/kg Klotho for 28 days post-MI. (Male C57BL/6 mice; N = 8) | Heart tissue sections (wheat germ agglutinin, Masson’s trichrome, and Sirius Red staining) at 28 days post-MI:  Fibrosis **↘**  HW/BW **↘**  HW/TL **↘** | Cardiac function via ECG at 28 days post-MI:  LVEF **↗**  Fractional shortening **↗**  SBP **~**  DBP **~**  HR **~** | Flow cytometry, TUNEL assay, and RT-PCR at 28 days post-MI:  Autophagy **↗**  α-MHC mRNA levels **↗**  Myocardial cell death **↘**  Cardiac hypertrophy **↘**  Hypertrophic/HF marker(s) (ANP, BNP, and β-MHC) **↘**  Inflammation **↘**  Survival at 28 days post-MI:  Mortality ~ | Wang K et al. (2022); doi.org/10.1016/j.mad.2022.111714^40^ |
| **Meteorin-like protein (METRNL)** KIT receptor on endothelial cells | Bolus injection of 2 µg recombinant mouse METRNL into the left ventricular cavity immediately post-MI. S.c infusion of METRNL for up to 7 days (2 µg/day).  (HF-prone FVB/N mice; N unclear) | Heart tissue sections (Evans Blue and TTC staining) at 24h and 28-days post-MI:  Infarct size **↘**  (At 28-days post-MI) | Cardiac function via ECG and LV pressure-volume loops at 28 days post-MI:  LVEF **↗**  Fractional area change **↗**  d*P*/dt_min_ **↗** Stroke work **↗**  𝜏 **↘**  LVEDP **~**  LVESP **~**  LVEDV **~**  LVESV **~** +d*P*/dt_max_ **~** Cardiac output **~**  HR **~**  LVESA **↘**  LVEDA **~** | TUNEL assay at 24h post-MI:  Myocardial cell death **~**  Immunofluorescence at 2-, 6-, and 28-days post-MI:  Angiogenesis **↗** | Reboll MR et al. (2022); doi.org/10.1126/science.abn3027^41^ |
| **Myeloid-derived growth factor (MYDGF)** c-Myc/FoxM1 signalling pathways | Intramyocardial injection of recombinant MYDGF (5 µg/mouse) into mice hearts post-MI, followed by continuous tail vein injection for 7 days (10 µg/day). (C57BL/6J mice; N = 25) | Heart tissue sections (Masson’s trichrome staining) at 21 days post-MI:  Infarct size **↘**  Fibrosis **↘** | Cardiac function via ECG at 21 days post-MI:  LVEF **↗** | Immunofluorescence at 7 days post-MI:  Cardiomyocyte proliferation **↗**  Survival at 21 days post MI:  Mortality **↘** | Wang Y et al. (2020); doi.org/10.7150/thno.44281^42^ |
| **Neuregulin 1 (NRG-1)** ErbB signalling pathway | IV administration of 10 µg/kg/day recombinant human NRG-1 1-week post-MI in rats for 5- or 10-days.  (Sprague Dawley rats; N varied per experiment) | Heart transverse sections (HE staining) at 5-days post-MI:  Infarct size **~** | Cardiac function via ECG at 5- and 10-days post-MI:  LVEDD **~**  LVESD **↘**  LVEF **↗**  Fractional shortening **↗**  Haemodynamic measurements via micromanometry at 33 weeks post-MI:  HR **~**  MAP **↗**  +d*P*/dt **↗**  -d*P*/dt **↗**  LVESP **↗**  LVEDP **↘** | Plasma angiotensin I and II, aldosterone, and renin levels by radioimmunoassay at 5-days post-MI:  Angiogenesis **↗**  Cardiac failure **↘**  Survival at >167 days post MI:  Mortality **↘** | Liu X et al. (2006); doi.org/10.1016/j.jacc.2006.05.057^43^ |
| **Neuregulin 1 (NRG-1)** ErbB signalling pathway | Daily i.p injections of NRG-1 (2.5 mg/mouse dissolved in 0.1% bovine serum albumin) administered one week after MI for 12 weeks.  (C57B1/6 mice; N varied per experiment) | Infarct size, HW/TL, cardiomyocyte area 1- and 14-weeks post-treatment initiation:  Infarct size **↘**  (At 12 -weeks post-treatment initiation)  Cardiomyocyte area **↘**  LVPWd **~**  HW/TL **~** | Cardiac function and LVEDD, LVESD, IVSd, and LVPWd 1, 4-, 8-, 12-, and 14-weeks post-treatment initiation:  LVEF **↗**  (At 4, 8, 12 and 14-weeks post-treatment initiation)  LVEDD **↘**  LVESD **↘**  IVSd **↗**  (At 12 and 14-weeks post-treatment initiation) | Recruitment of progenitor cells 1-week post-treatment initiation:  Recruitment of progenitor cells **~**  Apoptosis, cardiomyocyte proliferation 1- and 14-weeks post-treatment initiation:  Cardiomyocyte proliferation **↗**  Myocardial cell death **~** | Bersell K et al. (2009); doi.org/10.1016/j.cell.2009.04.060^44^ |
| **Neuropeptide Y (NPY)** Y1Ra receptor, p38 MAPK, NF-κB signalling pathways | I.p. injection of NPY (12.5, 25, 50, 100 or 200 µg/kg body weight) once every 24 hours for 7 consecutive days post-MI.  (129S-Npy^tm1Rpa^/J NPY WT mice; N varied per experiment) | LV transverse slides (TTC staining for infarct size and Masson’s trichrome staining for fibrosis) (12.5, 25, 50 and 100 µg/kg doses) at 7 days post-MI:  Infarct size **↘**  (At 25 µg/kg dose)  Fibrosis **↘** (At 25 µg/kg dose) | Cardiac function via ECG (12.5, 25, 50 and 100 µg/kg doses) at 7 days post-MI:  LVEF **↗**  LVAWs **↗**  LVAWd **↗**  Fractional shortening **↗**  (At 25 µg/kg dose)  LVIDd **↘**  LVIDs **↘**  (At 25 µg/kg dose) | TUNEL assay (apoptosis) (50, 100 and 200 µg/kg doses) at 7 days post-MI:  Apoptosis **↘**  Immunohistochemistry (angiogenesis) (25 µg/kg dose) 7 days post-MI:  Angiogenesis ↗  (Border and infarct zones) | Qin YY et al. (2022); doi.org/10.1016/j.ymthe.2021.10.005^45^ |
| **Human-like oncostatin M (hIOSM)** Jak/STAT, MAPK, and PI3K/Akt pathways | Repetitive i.p. injections of hlOSM (100 ng per gram of body weight and day) or sterile PBS (control) administered after the onset of MI and 1-, 2- and 3-days post-MI.  (Male C57BL/6J mice; N varied per experiment) | Myocardium infarct size via cardiac MRI at 28 days post-MI:  LVW **~**  Infarct size **~** | Cardiac function via cardiac MRI at 28 days post-MI:  Global longitudinal strain **↘**  Global circumferential strain **↘**  Basal regional circumferential strain **↘**  Midventricular regional circumferential strain **↘**  Apical regional circumferential strain **↘**  LVEDV **~**  LVESV **~**  LV stroke volume **~**  LV mass **~**  Global radial strain **~**  LVEF **~** | ND | Lörchner H et al. (2022); doi.org/10.3390/ijms23010353^46^ |
| **Oncostatin M (OSM)** Receptor O*b*, VEGF, bFGF signalling pathways | I.p. injection of OSM (60 ng/g of body weight) administered twice a day for 14 days post-MI.  (129-O*smr^tm1.1Nat^*/J mice; N varied per experiment) | Heart tissue staining (Masson’s trichrome and Weigert’s iron haematoxylin staining) at 14 days post-MI:  Fibrosis **↘**  HW/BW **↘**  LW/BW ratio ↘ | Cardiac function via ECG at 28 days post-MI:  LVEF **↗**  Fractional shortening **↗**  LVIDd **↘**  LVIDs **↘** | Immunohistochemistry, Western blot assay, and TUNEL assay at 14 days post-MI:  Angiogenesis **↗**  Myocardial cell death **↘** | Zhang X et al. (2015); 53:11; doi.org/10.1155/2015/317905^47^ |
| **Osteopontin (OPN)** Receptor CD44, YAP1 | Injection of 200 ng recombinant OPN in 40 μL of PBS or PBS alone (control) to the border of the ischaemic zone 1 min post-MI.  (Female C57BL/6 mice; N = 20) | Heart tissues slices (HE or picrosirius red staining) at 30 days post-MI:  Scar thickness **↗**  Scar length **↘**  Expansion index **↘**  Total scar area **~**  Whole LV area **~**  LV cavity area **~**  Muscle area **~** | Cardiac function via ECG at 30 days post-MI:  Fractional area change **↗**  IVSd **↗**  IVSs **↗**  PWd **↗**  PWs **↗**  Contractility **↗**  Recovery of specific infarcted segments **↗**  LVVD **~**  LVVS **~**  Fractional shortening ~  LVEF **~**  LVESA **↘**  LVEDA **~**  LVESD **↘**  LVEDD **~** | Cardiomyocyte staining with antibodies at 3 days post-MI:  Cardiomyocyte cell-cycle re-entry **↗** | Rotem I et al. (2022); doi.org/10.1007/s00395-022-00957-0^48^ |
| **Prominin-1-derived peptide (PR1P)** VEGF | I.p. administration of PR1P (scrambled peptide used as control) starting after MI, followed by every other day until 12 days post-MI; 10 µg PR1P in 100 μL saline in mice (female C57BL/6J mice; N = 18) and 200 µg PR1P in 100 μL saline in rats (female Sprague-Dawley rats; N = 9) | **Rat model:**  Heart tissue sections (Masson’s trichrome staining) at 3- and 14-days post-MI:  LV cavity area **↘**  Infarct size **↘**  (At 3 days post-MI) | **Mouse and rat models:**  Cardiac function via ECG and haemodynamic measurements (microtip pressure-volume with catheterisation) at 14 days post-MI:  **Mouse model:**  LVEF **↗**  Fractional shortening **↗**  Stroke volume **↗**  Stroke work **↗**  Cardiac output **↗**  Max LV volume **↘**  Min LV volume **↘**  Min LVEDP **~**  HR **~**  Max LVP **~**  Min LVP **~**  -d*P*/dt_min_ **~**  +d*P*/dt_max_ **~**  Max systolic aortic pressure **~**  Min diastolic aortic pressure **~**  **Rat model:**  Fractional area change **↗**  Fractional shortening **↗**  **Mouse and rat models:**  LVESD **↘**  LVEDD **~** | N/A | Adini A et al. (2021); doi.org/10.3390/  ijms22105169^1^ |
| **Thymosin β4** ILK, Akt pathway | I.p. injection of thymosin β4 (150 µg in 300 µL PBS) or PBS alone (control) immediately after MI, with administration repeated every day for 3 days post MI (male C57BL/6J mice; N = 45)  (also intracardiac injection of thymosin β4 (400 ng in 10 µL collagen or collagen alone immediately after ligation ± i.p. thymosin β4) | Heart tissue sections (trichrome staining) at 14 days post-MI:  Infarct size **↘** | Cardiac function via ECG  At 4 weeks post-MI:  LVEDD **↘**  LVESD **↘**  At 2 and 4 weeks post-MI:  LVEF **↗**  Fractional shortening **↗** | Immunohistochemistry (TUNEL assay) at 24 hours post MI:  Myocardial cell death **↘**  Cell survival and stem cell recruitment (time point unclear):  Cell survival **↗**  Stem cell recruitment **~** | Bock-Marquette I et al. (2004); doi.org/10.1016/j.intimp.2023.109741^49^ |
| **Tissue inhibitor of matrix metalloproteinase-3 (TIMP-3)** MMP | Intramyocardial injection of N domain-TIMP3 (1 mg/rat), TIMP3v2 (glycosylation mutation; 2 mg/rat), TIMP3v82 (glycosylation mutation; 2 mg/rat); vehicle (control) 3 hours post-MI.  (Male Sprague Dawley rats; N = 8–10 per group) | N/A | Cardiac function via ECG at 3- and 7-days post-MI:  (Only LVEF data captured here due to unclear statistical analyses):  LVEF **↗**  (At days 3 and 7 post-MI for TIMP3v82 and at 3 days post-MI for N domain-TIMP3 and TIMPv2) | N/A | Chintalgattu V et al. (2018); doi.org/10.1002/prp2.442^50^ |
| **Vascular endothelial growth factor C (VEGF-C)** Lymphatic endothelial cells | I.p. injection of 0.1 µg/g recombinant human VEGF-C (Cys156Ser) or PBS (control) at days 0, 2, 3, 4 and 6 post-MI. (Female C57BL/6 mice; N varied per experiment) | Heart tissue sections (HE staining) at 7-, 14-, 21- and 28-days post-MI:  LVW **~**  Infarct size **~** | Cardiac function via cardiac cine-MRI at 7-, 14-, 21- and 28-days post-MI:  LVEF **↗**  (At 14- and 21-days post-MI)  LVESV **↘**  (At 14 days post-MI)  HR **~**  LVEDV **~**  Stroke Volume **~**  Cardiac output **~** | Lymphangiogenic response via RT-PCR at 7 days post-MI:  Angiogenesis **↗** | Klotz L et al. (2015); doi.org/10.1038/nature1448^51^ |
| **Vaspin** PI3K/Akt pathway | I.p. injection of 320 ng/kg vaspin or saline (control) daily for 4 weeks post-MI.  (Male Sprague Dawley rats; N = 4–8 per experiment) | Heart tissue sections (Masson staining) at 4 weeks post-MI:  Fibrosis **↘** | Cardiac function via ECG and haemodynamic measurements (micromanometer with catheterisation) at 4 weeks post-MI:  LVEDP **↘**  LVSP **↗**  LVVS **↘**  LVVD **↘**  LVEF **↗**  Fractional shortening **↗**  +d*P*/dt_max_ **↗**  LVIDd **↘**  LVIDs **↘** | RT-PCR, immunofluorescence, Western blot, chemiluminescence at 4 weeks post-MI:  Oxidative stress **↘**  Hypertrophic/HF marker(s) (ANP and BNP) **↘** | Ji M et al. (2022); doi.org/10.1097/fjc.0000000000001291^52^ |

**↗ =** Increase in a parameter; **↘ =** decrease in a parameter**; ~** = No significant change in a parameter. Changes in a parameter are relative to controls included in each study (e.g. untreated MI). Colours of arrow and tilde symbols denote a positive effect (**green**), negative effect (**red**), and neutral effect (**orange**) of a protein-based therapy regarding limiting damage and/or mediating cardiac repair when administered post-MI.

+d*P*/dt_max_, maximal rate of increase in left ventricular pressure; -dP/dt_max_, maximal rate of decrease in left ventricular pressure; Akt, protein kinase B; Α-MHC, α-myosin heavy chain; AMI, acute myocardial infarction; ANP, atrial natriuretic peptide; APJ, apelin peptide jejunum or apelin-angiotensin receptor-like 1; β-MHC, β-myosin heavy chain; bFGF, basic fibroblast growth factor; BNP, brain natriuretic peptide; c-KIT, receptor tyrosine kinase; CTGF, connective tissue growth factor; cTnT, cardiac troponin T; Dag1, alpha-dystroglycan; DBP, diastolic blood pressure; dLVP, developed left ventricular pressure; E/A, ratio of peak early diastolic ventricular filling velocity to peak atrial filling velocity; ECG, electrocardiogram; ELISA, enzyme-linked immunosorbent assay; ErbB, receptor family members; ERK, extracellular signal-regulated kinase; FACS, fluorescence-activated cell sorting; FGF21, fibroblast growth factor; FPR, formyl-peptide receptor; GHS-R, growth hormone secretagogue receptor; HE, haematoxylin and eosin; HF, heart failure; HO-1, haem oxygenase-1; HR, heart rate; HW/BW, heart-weight-to-body-weight ratio; HW/TL, heart-weight-to-tibial length ratio; ILK, integrin-linked kinase; i.p., intraperitoneal; IVSd, intraventricular septum thickness in diastole; IVSs, intraventricular septum thickness in systole; LV/BW, left ventricle-to-body weight ratio; LVAWd, left ventricular anterior wall diastolic thickness; LVAWs, left ventricular anterior wall systolic thickness; LVEDA, left ventricular end-diastolic area; LVEDD, left ventricular end-diastolic diameter; LVEDD/BW, left ventricular end-diastolic dimension-to-body- weight ratio; LVEDP, left ventricular end-diastolic pressure; LVEDV, left ventricular end-diastolic volume; LVEF, left ventricular ejection fraction; LVESA, left ventricular end-systolic area; LVESD, left ventricular end-systolic diameter; LVESP, left ventricular end-systolic pressure; LVESV, left ventricular end-systolic volume; LVIDd, left ventricular internal diameter at end-diastole; LVIDs, left ventricular internal diameter at end-systole; LVP, left ventricular pressure; LVPWd, left ventricular posterior wall thickness in diastole; LVPWs, left ventricular posterior wall thickness in diastole; LVSP, left ventricular systolic pressure; LVVD, left ventricular volume in diastole; LVVS, left ventricular volume in systole; LVW/BW, left ventricular free wall weight-to-body weight ratio; LW/BW, lung weight-to-body weight ratio; MAP, mean arterial pressure; MAPK, mitogen-activated protein kinase; METRNL, meteorin-like; MI, myocardial infarction; MMP, matrix metalloproteinase; MRI, magnetic resonance imaging; mTOR, mammalian target of rapamycin; N/A, not available; ND, not disclosed; NF-κB, nuclear factor kappa B; NOX, NADPH oxidase; NRG, neuregulin; OPN, osteopontin; PBS, phosphate-buffered saline; PI3K, phosphatidylinositol 3-kinase; Pmax, maximum pressure; PR1P, prominin-1-derived peptide; PWd, posterior wall thickness in diastole; PWs, posterior wall thickness in systole; Rac1, Ras-related C3 botulinum toxin substrate 1; RT-PCR, reverse transcription polymerase chain reaction; RV, right ventricular; RVESP, right ventricular end-systolic pressure; RVW/BW, right ventricular weight-to-body weight ratio; SBP, systolic blood pressure; s.c., subcutaneous; SOD2, Superoxide dismutase 2; STAT3, signal transducer and activator of transcription 3; 𝜏, time constant of left ventricular isovolumic pressure decline; TGF-*β,* transforming growth factor *β*; TIMP, tissue inhibitor of metalloproteinases; TnI, troponin I; TLR, toll-like receptor; TTC, triphenyl tetrazolium chloride; TUNEL, Terminal deoxynucleotidyl transferase dUTP nick end labelling; VEGF, vascular endothelial growth factor; YAP, yes-associated protein.

**Table S10.** *In vivo* small animal models of ischaemia/reperfusion injury (n = 23) investigating protein-based therapies that demonstrate an ability to limit damage and/or mediate cardiac repair when administered post-MI

| **Proteins, in alphabetical order** | **Administration method, timeline (when/for how long administered after MI), dosage(s)** | **Histomorphological endpoints with timepoint(s) when assessed after MI** | **Functional outcome, method (e.g. echo, MRI) with timepoints when assessed after MI** | **Other outcomes** | **Reference for protein therapy study** |
| --- | --- | --- | --- | --- | --- |
| **Alpha-1-antitrypsin (AAT)/prolastin C**  Caspase-1 inhibitor | I.p. injection of 60 mg/kg AAT or albumin (control) immediately after reperfused AMI and then daily for 7 days.  (Male outbred ICR [CD1] mice; N = 6–21 per group) | Histology of heart transverse slides (Masson’s trichrome and TTC staining) at 1 and 7 days after AMI:  Infarct size at 1 day ↘  Infarct size at 7 days ↘  HR **~** | Transthoracic ECG at 1 and 7 days after AMI (prior to sacrifice):  LVEF **↗** (at 1 and 7 days)  LVEDD **↘** (at day 7)  LVESD **↘** (at day 7)  LVAWDT **↗** (at 7 days)  LVAWST ↗ (at 1 and 7 days)  LVW **~** | Heart homogenates (24 h after AMI) (AAT vs control):  Caspase-1 **↘**  Caspase-3 **~** | Toldo S et al. (2011); doi.org/10.1016/j.yjmcc.2011.05.003^4^ |
| **Alpha-1-antitrypsin (AAT)/prolastin C**  Inflammation | **30 mins ischaemia:**  I.p. injection of 60, 120, or 180 mg/kg of prolastin C or vehicle (control) at reperfusion.  (Male CD1 mice; N = 6–8)  **75 mins ischaemia:**  I.p. injection of 60 mg/kg of prolastin C or vehicle (control) at reperfusion. (Male CD1 mice; N = 6–8)  **Delayed reperfusion:**  I.p. injection of 180 mg/kg of prolastin C or vehicle (control) 30 mins after MI-reperfusion.  (Male CD1 mice; N = 6–8)  **Multiple administrations:**  I.p. injection of 60 mg/kg of prolastin C or vehicle (control) at reperfusion, with daily administrations for 7 days.  (Male CD1 mice; N = 6–8) | **30 mins ischaemia:**  Heart transverse slices (TTC and Evans blue staining) at 24 hours post-reperfusion:  Infarct size **↘**  **75 mins ischaemia or delayed reperfusion:**  Heart transverse slices (TTC and Evans blue staining) at 24 hours post-reperfusion:  Infarct size **↘** | **30 mins ischaemia:**  Cardiac function by ECG at 24 hours post-reperfusion:  Wall motion score index **↘**  **Multiple administrations:**  Cardiac function by ECG at 7 days post-reperfusion:  LVEF **↗**  LVEDV **↘** | **30 mins ischaemia:**  Serum cardiac troponin I (ELISA) at 24 hours post-reperfusion:  Myocardial damage (cardiac TnI) **↘** | Mauro AG et al. (2017); doi.org/ 10.1097/fjc.0000000000000474^53^ |
| **Apelin-13**  VEGFA, Kdr,  Ang-1, Tie2 and eNOS signalling pathways | Apelin-13 was dissolved in normal saline and administered i.p. 24h after induction of MI (10 nmol/kg). Successive doses were administered once a day for 5 days post-MI or saline (control).  (Male Wistar rats; N = 36) | Histology of heart transverse sections in infarcted areas of LV at 14 days post MI (apelin-13 vs control):  Fibrosis (% of LV) **↘** | Haemodynamic monitoring at 14 days post MI (apelin-13 vs control):  LVSP **↗**  dLVP **↗**  RPP **↗**  +d*P*/dt_max_ **↗** -d*P*/dt_max_ **↗**  LVEDP **↘** | Angiogenesis (mRNA expression in LV peri-infected area) and myocardial neovascularisation (IHC) at 14 days post MI (apelin-13 vs control):  VEGF-A, Kdr, Ang I, Tie2, eNOS ↗  Capillary density ↗ | Azizi Y et al. (2015); doi.org/10.1016/j.ejphar.2015.04.034^54^ |
| **Apelin-13**  Receptor APJ, PI3K/akt, ERK, MAPK, eNOS signalling pathways | IV infusion of 0.1, 1, or 10 µg/kg (Male Sprague-Dawley rats; n = 8), 1 µg/kg (n = 8), 10 µg/kg apelin (n = 8), or no treatment (control, n = 8) 15 minutes before end of ischaemic period vs reperfusion alone (control) | Histology of heart transverse sections in infarcted areas of LV at 2 h post MI/reperfusion (all apelin-13 doses vs control):  Infarct size **↘** | Haemodynamic monitoring at 2 h, 12 h, and 24 h post MI/reperfusion (1 µg/kg apelin-13 vs control):  MAP **↗**  +d*P*/dt_max_ **↗**  -d*P*/dt_min_ **↗**  LVEDP **↘**  HR **~** | Mortality rate during the 24 h reperfusion process (apelin-13 vs control) **~**  ER-dependent stress signalling markers at 2 h, 12 h, and 24 h post MI/reperfusion (apelin-13 vs control):  GRP78 **↘**  CHOP **↘**  JNK (phosphorylated and total) **↘**Caspase-12 (cleaved) ↘ | Tao J et al. (2011); doi.org/10.1152/ajpheart.00097.2011^55^ |
| **Apyrase** Markers of inflammation and pyroptosis | IV recombinant apyrase (0.1, 0.3, 1.0, 3.0 or 5.0 mg/kg) or vehicle (control) administered pre-reperfusion, at 25 min of ischaemia. (Sprague-Dawley rats; N varied per experiment). Reperfusion lasted for 24h  IV recombinant apyrase (1.0 or 3.0 mg/kg) or vehicle (control) administered pre-reperfusion, at 25 min of ischaemia.  (Sprague-Dawley rats; N varied per experiment). Reperfusion lasted for 4 weeks | Transverse LV sections by TTC staining obtained 24h after reperfusion (apyrase ≥0.3 mg/kg vs control):  Area at risk **~**  Infarct size **↘**  Cardiomyocytes from tissue sections stained with HE and Masson’s trichrome obtained after 4 weeks reperfusion (apyrase vs control):  Fibrosis **↘** | ECG at 4 weeks post MI (apyrase vs control):  LVEF **↗**  Fractional shortening **↗**  LVEDV **↘**  LVESV **↘**  IVSd **↘**  IVSs **↘**  LVEDD **↘**  LVESD **↘**  (Improvements in parameters were dose dependent)  Pressure transducer on left carotid artery after reperfusion (apyrase vs control):  Mean arterial blood pressure **~**  HR **~** | Immunoblotting from ischaemic area of LV and mRNA expression 24h after reperfusion (apyrase vs control):  IL-15 **↘**  MCP1 **↘**  TNFα **↘**  IL-6 **↘**  RIP1 **↘**  RIP3 **↘** | Birnbaum Y et al. (2022); doi.org/10.1007/s10557-022-07329-9^56^ |
| **Apyrase** ATP | Human recombinant apyrase APT102 (0.3 mg/kg) or placebo (control) was injected via the tail vein at 15 minutes before reperfusion, and then at 1, 2, 3, and 4 days afterward.  (Female Sprague-Dawley rats; N = 27) | Histopathology of heart sections obtained at 1, 2, 3, and 5 days after reperfusion and stained with HE apyrase vs control):  Infarct size **↘**  Oedema size **↘**  Intramyocardial haemorrhage size **↘**  Microvascular obstruction size **↘**  Left ventricular myocardial volume **~** | Cardiovascular MRI on days 1, 2, 3, and 5 after reperfusion (apyrase vs control):  LVEF **↗**  LVESV **↘**  LVEDV **~**  Stroke volume **~** | ND | Xu Z et al. (2020); doi.org/10.3348/kjr.2019.0853^57^ |
| **B*β*_15–42_** Inhibits proinflammatory cytokine release | **Acute myocardial ischaemia-reperfusion models (25 min occlusion + 2 hours reperfusion):**  IV injection of 2.4 or 7.2 mg/kg human B*β*_15–42_ or random peptide (control) at the start of reperfusion.  (Male Wistar rats and C57/BL6 mice; N = 10–14)  **Chronic myocardial ischaemia-reperfusion model (25 min occlusion + 30 days reperfusion):**  IV injection of 2.4 mg/kg hB*β*_15–42_ or random peptide (control) at the start of reperfusion.  (Male Wistar rats; N = 6–9) | Infarct size (pNBT staining) and fibrosis of heart sections (Masson’s trichrome staining) in AMI (2 h reperfusion) and chronic MI (30 days reperfusion) (Bβ15–42 vs random peptide):  AMI model: infarct size **↘**  Chronic MI model: infarct size **↘**  Chronic MI model fibrosis **↘** | AMI model: haemodynamic monitoring of right carotid artery via pressure transducer 2 h post MI-reperfusion (Bβ15–42 vs random peptide):  Mean arterial blood pressure **~**  HR **~**  Pressure rate index **~** | Plasma cytokines from wild-type BALB/c mice after AMI-reperfusion (20 min of ischaemia followed by 2 h of reperfusion) (Bβ15–42 vs random peptide):  TNF-α **↘**  IL-1 **↘**  IL-6 **↘**  IL-10 **~**  IL-12 **~**  Plasma cytokines from fibrogen⁻^/^⁻ mice after AMI-reperfusion (20 min of ischaemia followed by 2 h of reperfusion) (Bβ15–42 vs random peptide):  TNF-α **~**  IL-1 **~**  IL-6 **~**  IL-10 **~**  IL-12 **~** | Zacharowski K et al. (2007); doi.org/10.1097/SHK.0b013e31802fa038^58^ |
| **Endoplasmic reticulum membrane protein complex subunit 10 (EMC-10)** Small GTPases, PAK2, p38 MAPK | S.c injection of 10 µg recombinant EMC10 or diluent (control) at the time of reperfusion followed by s.c infusion for 7 days (10µg/day).  (Male FVB/N mice; N = 10) | Tissues sections stained with Masson’s trichrome 28 days after MI (EMC10 vs control):  Infarct size **↘**  Capillary density by wheat germ agglutinin staining 28 days post MI (EMC10 vs control):  Myocardial cells (IB4⁺) **↗** | High-resolution 2D transthoracic ECG and invasive pressure-volume measurements of LV at days 6 and 28 post-MI (EMC10 vs control):  Fractional area change **↗**  LVESA **↘** | Survival was assessed daily up to 28 days post-MI (EMC10 vs control):  Mortality **↘**  Angiogenesis at day 28 post-MI (EMC10 vs control):  Angiogenesis **↗** | Reboll MR et al. (2017); doi.org/10.1161/CIRCULATIONAHA.117.029980^59^ |
| **Erythropoietin (EPO)**  Endothelial progenitor cells mobilisation | Weekly i.p. injection of 1.5 µg/kg of recombinant human EPO or vehicle (control) for 8 weeks. EPO was first administered 7 days post-MI.  (Male Sprague-Dawley rats; N = varied per group) | Heart tissue sections (TTC staining) post death:  Infarct size **↘**  HW/BW **~**  LVW/BW **~**  RVW/BW **~** | Cardiac function via ECG and pressure-volume catheter after 8 weeks of treatment:  AWd **↗**  AWs **↗**  +d*P*/dt_max_ **↗**  LVEDD **~**  LVESD **~**  HR **~**  Fractional shortening **~**  SBP **~**  DBP **~**  LVSP **~**  LVEDP **~**  PRSW**~**  -d*P*/dt_max_ **~** | Haematologic analyses, and angiogenesis post-death:  Capillary density **↗**  Stem cell mobilisation **↗**  Mortality **~**  Haematocrit **↗** | Prunier F et al. (2007); doi.org/10.1152/ajpheart.00357.2006^60^ |
| **Erythropoietin (EPO)**  JAK2/STAT5, Akt-1, MAPK signalling pathways | I.p. injection of recombinant human EPO (5000 IU/kg in 0.5 mL of saline) or saline (control) administered at 2 different time points: at the start of ischaemia (EPO-isch group, male Sprague-Dawley rats; n = 16), and 5 minutes after the onset of reperfusion (EPO-rep group, n = 20); sham-operated with saline administration (SHAM group, n = 6)  (coronary occlusion for 45 min with 24 h of reperfusion) | Heart tissue sections (nitro blue tetrazolium staining) at 24h post-coronary reperfusion:  **EPO-isch, and -rep dose groups:**  Infarct size **↘** | Cardiac function (via pressure transducer with catheterisation) at 24h post-coronary reperfusion:  **EPO-isch and -rep dose groups:**  LVEDP **↘**  +d*P*/dt_max_ **~**  LVSP **~**  MAP **~**  **EPO-rep dose group:**  -d*P*/dt_max_ **↗**  **EPO-isch dose group:**  -d*P*/dt_max_ **~** | Apoptosis and cell proliferation (immunohistochemistry) at 24h post-coronary reperfusion:  **EPO-isch, and -rep dose groups:**  Apoptosis **↘**  Cell proliferation **~** | Lipsic E et al. (2004); doi.org/10.1097/01.fjc.0000140209.04675.c3^61^ |
| **Erythropoietin (EPO)**  JAK2/STAT, PI3K/Akt, eNOS signalling pathways | IV injection of 5000 unit/kg EPO administered either 15 min after ischaemia (EPO-ischaemia) or at the onset of reperfusion (EPO-reperfusion), or vehicle (control).  (Male Wistar rats; n = varied per experiment) | Heart tissue sections (TTC and Evans blue staining) at the end of reperfusion:  **EPO-ischaemia dose group:**  Infarct size **↘**  Apoptosis **↘**  **EPO-reperfusion dose group:**  Infarct size **~**  Apoptosis **~** | Cardiac function during cardiac ischaemia and through the end of reperfusion (pressure transducer with catheterisation):  **EPO-ischaemia dose group:**  LVEF **↗**  **EPO-reperfusion dose group:**  LVEF **~** | Mitochondrial function at the end of reperfusion:  **EPO-ischaemia dose group:**  Arrhythmia **↘**  Mitochondrial dysfunction/fission **↘**  **EPO-reperfusion dose group:**  Arrhythmia **~**  Mitochondrial dysfunction/fission **~** | Benjanuwattra J et al. (2022); doi.org/10.1016/j.yexmp.2022.104802^62^ |
| **Extracellular domain of the p75 neurotrophin receptor (p75ECD)** p-JNK/caspase 3 pathway | Tail vein injection of 1, 3, or 9 mg/kg p75ECD 5 minutes before reperfusion (vs I/R alone).  (Male Sprague-Dawley rats; n = 8 per group) | Heart slices (TTC staining) at 24 hours post-reperfusion (1, 3 and 9 mg/kg dose groups):  **1 mg/kg dose group:**  Infarct size **~**  **3 and 9 mg/kg dose groups:**  Infarct size **↘**  Heart slices (Masson’s trichrome staining or immunofluorescence staining) at 28 days post-reperfusion (3 mg/kg dose group):  Fibrosis **↘**  Microvascular dysfunction **↘** | Cardiac function (by ECG) 28 days post-reperfusion (3 mg/kg dose group):  LVEDD **↘**  LVESD **↘** | Pro-neurotrophin expression (Western blot) 24h and 28 days post-MI (3 mg/kg dose group):  Harmful pro-neurotrophin expression **↘** | Fang J et al. (2020); doi.org/10.1161/JAHA.119.016047^63^ |
| **Galanin** Receptor GalR1-3 signalling pathway | IV injection of galanin peptides at various doses based on the optimum dose according to ability to reduce infarct size (G1 – 0.5 mg/kg; G2 – 2.0 mg/kg; G3 – 1.0 mg/kg; G4 – 0.5 mg/kg; G5 – 1.0 mg/kg; vehicle [control]).  (Male Wistar rats; n = 10 per dose group) | LV transverse sections (Evans blue and TTC staining) and markers of necrosis (plasma CK-MB and LDH levels) at the end of reperfusion:  **G1–G5 peptides**:  Infarct size **↘**  Necrosis **↘** | Cardiac function (arterial blood pressure by pressure transducer) at 2–3 and 60 minutes of reperfusion:  **G1 peptide:**  SAP **↘**  (At 2–3 minutes of reperfusion)  **G5 peptide:**  HR **↗**  (At 2–3 minutes of reperfusion)  **G2–G5 peptides**:  SAP **~** (At 2–3 min and at 60 min of reperfusion)  **G2 peptide:**  HR **↘** (At 2–3 min of reperfusion)  **G1, G3 and G4 peptides:**  HR **~** (At 2–3 min and at 60 min of reperfusion) | ND | Serebryakova L et al. (2019); doi.org/10.1016/j.peptides.2018.05.001^64^ |
| **Granulocyte colony-stimulating factor (G-CSF)** Expression of cytokines and MMPs | S.c injection of recombinant human G-CSF (10 µg/kg/day) or saline (control) administered 24h post-MI for 1 day in the 2-day reperfusion group (male Japanese White rabbits; n = 15),  for 5 days in the 7-day reperfusion group (n = 15), the 14-day reperfusion group (n = 15), and the 3-month reperfusion group (n = 15) | LV wall thickness at the end of reperfusion in the 14-day and 3-month reperfusion groups:  LV anterior wall thickness/ LV posterior wall thickness without infarction **↗**  LV weight, risk area and infarct size at the end of reperfusion in the 48-hour, 7-day, 14-day, and 3-month reperfusion groups:  Infarct size **↘**  (At 7 days, 14 days, and 3-months post-MI)  LVW **~**  Fibrosis (collagen areas) at the end of reperfusion in the 14-day and 3-month reperfusion groups:  Fibrosis **↘** | Cardiac function, LVEDD, LVESD (by ECG) at the end of reperfusion in the 14-day and 3-month reperfusion groups:  LVEF **↗**  Fractional shortening **↗**  HR **~**  Blood pressure **~**  LVEDD **↘**  LVESD **↘** | Peripheral blood cell counts at the end of reperfusion in the 7 day and 14-day reperfusion groups:  STEM cell mobilisation (WBC count) **↗**  (At 7 days post-MI)  Inflammation, myocyte size, at the end of reperfusion in the 48-hour, 7-day, 14-day, and 3-month reperfusion groups:  Inflammation **↘**  (At 14 days and 3 months post-MI)  Myocyte size **~**  Angiogenesis at the end of reperfusion in the 14-day and 3-month reperfusion groups:  Microvessels with CD31^+^ cells and myofibroblasts with positive α-smooth muscle actin in infarcted tissues **↗**  (At 14 days post-MI)  Mortality **~** | Minatoguchi S et al. (2004); doi.org/10.1161/01.Cir.0000129770.93985.3e^65^ |
| **High-density lipoprotein (HDL)** Akt signalling pathway | IV bolus via tail vein injection of reconstituted HDL (80 mg/kg body weight) or saline (control) administered at the onset of reperfusion, post-MI.  (Male C57BL/6 mice; N = 14–18) | LV sections (Masson’s trichrome, Sirius red, endothelial cell BS-1 lectin, and wheat germ agglutinin staining) at 15 days post-MI:  Infarct size **↘**  Fibrosis (collagen deposition) **↘**  Capillary density **↗** (peri-infarct region) | Cardiac function (by ECG) 15 days post-MI:  LVEF**↗**  Cardiac output **↗**  Stroke volume **↗**  HR **↘** | ND | Heywood SE et al. (2017); doi.org/10.1126/scitranslmed.aam6084^66^ |
| **Insulin-like growth factor-1 (IGF-1)** Immune-modulatory cytokine (M2-like macrophages) | S.c. bolus of mature recombinant IGF-1 (40 ng/g) or vehicle (control) at the start of reperfusion, post-MI. IGF-1 was administered continuously for the first 3 days (1 µg/g/day) of 1 or 4 weeks of reperfusion using micro-osmotic pumps.  For experiments relating to the measurement of infarct size, mice were subjected to 2h of reperfusion, with IGF-1 administered at the start.  (C57BL/6J mice; N varied per experiment) | Heart tissue sections (immunofluorescence, Masson’s trichrome staining, Evans blue and TTC) at hour 2 and week 1 of reperfusion; fibrosis 1 week post MI:  Scar size **~** (after 2 h of reperfusion)  Scar size **↘** (At week 1 of reperfusion)  Fibrosis ~ (At week 1 of reperfusion) | Cardiac function (by ECG) 1- and 4-weeks post-MI:  LVEF **↗**  Fractional area change **↗**  Stroke volume **↗**  (At 1-week post-MI)  Radial displacement, radial strain and circumferential strain in the ischaemic and remote myocardium, 1 week post MI:  Radial displacement **↗**  Radial strain **↗**  Circumferential strain **↘**  (In the ischaemic myocardium only, not remote) | Immunofluorescence imaging at 1 week post MI:  Capillary density **↗** (in border zone and scar area)  Inflammation 3 days post-MI:  Inflammation **↘** | Heinen A et al. (2019); doi.org/10.1016/j.ymthe.2018.10.020^67^ |
| **Interleukin-37 (IL-37)** TLR-4, NF-κB, IL-10 | Recombinant human IL-37 (2 µg) or vehicle (control) administered via the tail vein before reperfusion in ischaemia/reperfusion mice. Additional IL-37 administration every 24 hours over a 72-hour period post-MI.  (Male C57BL/6 mice; N = 6–8) | Heart tissue sections (TTC staining) at 24- and 72-hours post-reperfusion:  Infarct size **↘** | Cardiac function (by ECG) 24- and 72-hours post-reperfusion  LVEF **↗**  Fractional shortening **↗** | Cardiomyocyte damage and apoptosis (TUNEL assay) 24 hours post- reperfusion:  Myocardial damage (cTnT) **↘**  Myocardial cell death **↘**  Immunohistochemistry at 4 hours post-reperfusion (serum levels, vs I/R alone):  TNF-α **↘**  IL-6 **↘**  IL-1β **↘**  IL-10 **↗**  TGF-*β* **↗** | Wu B et al. (2014); doi.org/10.1111/cei.12284^68^ |
| **Irisin** SOD2 pathway | IV injection of 1 µg/kg recombinant irisin or vehicle (control) prior to reperfusion.  (Sprague-Dawley rats; N varied per experiment) | Heart tissue sections (TTC staining) at 24 hours post-MI:  Infarct size **↘** | Cardiac function (by ECG) 24 hours post-MI:  LVEF **↗** | Myocardial cell death and damage 24 hours post-MI:  Myocardial cell death **↘**  Myocardial damage (cardiac TnI) **↘** | Wang Z et al. (2018); doi.org/10.1097/FJC.0000000000000608^69^ |
| **Myeloid-derived growth factor (MYDGF)** PI3K-AKT, MAPK-STAT3 signalling pathways | **Reperfusion onset:**  IV bolus injection of recombinant MYDGF (10 µg) or PBS (control) into the LV cavity at the onset of reperfusion followed by a continuous s.c. infusion for 7 days via osmotic minipumps (10 µg/day).  (Male C57BL/6N mice; N = 25)  **Delayed treatment:**  IV bolus injection of recombinant MYDGF (10 µg) into the LV cavity 6 hours post-reperfusion followed by a continuous s.c. infusion for 7 days via osmotic minipumps (10 µg/day).  (FVB/N mice; N = varied) | **Reperfusion onset:**  LV sections (TTC and Evans blue staining) at 24 hours post-reperfusion:  Infarct size **↘**  LV sections (TTC and Evans blue staining) at 28-days post-reperfusion:  Infarct size **↘**  **Delayed treatment:**  LV sections (TTC and Evans blue staining) at 28-days post-reperfusion:  Infarct size **↘** | **Reperfusion onset:**  Cardiac function (by ECG) 6- and 28-days post-reperfusion:  LVESA **↘**  LVEDA **↘**  (At 28-days post-reperfusion)  Fractional area change **↗**  **Delayed treatment:**  Cardiac function 28-days post-reperfusion:  Fractional area change **↗** | **Reperfusion onset:**  Myocardial cell death 24 hours post-reperfusion:  TUNEL^+^ cardiac myocyte nuclei **↘**  Angiogenesis 2-, 6-, and 28-days post-MI:  Ki67^+^ IB4^+^ cells and IB4^+^ capillaries **↗**  Mortality **↘** | Korf-Klingebiel M et al. (2015); doi.org/10.1038/nm.3778^70^ |
| **Neurotrophin 3 (NT-3)** ERK-Bim pathway | Injection of human recombinant NT-3 (0.5 µg in 20 µL PBS) or PBS (control) into peri-infarct regions at 3 sites at the onset of reperfusion. This was followed by continuous NT-3 release (0.5 µg/day) for up to 1 week via a s.c. pump. (Male C57BL/6 mice; N varied per experiment) | Heart tissue sections (TTC and Evans blue staining) at 1-day post-MI:  Infarct size **↘**  Heart tissue sections (Masson’s trichrome and FITC-labelled wheat germ agglutinin staining) at 28-days post-MI:  Scar area **↘** | Cardiac function (by ECG) 2-, 7- and 28-days post-MI:  LVEF **↗**  Fractional shortening **↗**  (At day 28 post-MI)  LVDs **~** | Cardiomyocyte apoptosis (TUNEL assay) at 1-day post-MI:  Myocardial cell death **↘**  Angiogenesis 28-days post-MI:  CD31^+^ endothelial cells and α-SMA^+^ blood vessels in the infarct and border zones **↗** | Bi W et al. (2021); doi.org/10.1002/sctm.20-0456^71^ |
| **Relaxin**  Receptor RXFP1, VE-cadherin expression | I.p administration of a human recombinant relaxin peptide bolus (50 µg/kg via osmotic minipump) immediately after 1 or 4 hours of coronary artery occlusion, prior to 24 hours of reperfusion. Relaxin was subsequently administered at 50 µg/kg/day for 4 weeks (or vehicle [control]). (Male C57B1/6 mice; N varied per experiment) | **1 hour of occlusion:**  LV sections (Evans blue and TTC staining) at hour 24 of reperfusion:  Infarct size **~**  LV sections (Picrosirius Red staining for cardiac fibrosis) and organ weight at week 4 of reperfusion:  Atria/TL **~**  Lung/TL **~**  Fibrosis **~**  LV/TL**↘**  RV/TL **↘** | **1 hour of occlusion:**  Cardiac function (by ECG) at week 2 of reperfusion:  LVEF **↗**  LVEDV **~**  LVESV **~**  HR **~**  LVESA **~**  LVEDA **~**  Fractional area change **~** | **4 hours of occlusion:**  Microvascular obstruction at hour 24 of reperfusion:  Microvascular obstruction **↘**  **1 hour of occlusion:**  Lumen opened capillaries, microvascular leakage at hour 24 of reperfusion:  Lumen opened capillaries **↗**  Microvascular leakage **↘**  Inflammation at hour 48 of reperfusion (RT-PCR of cytokines in ischaemic myocardium):  IL-1β **↘**  IL-6 **↘**  TNFα **~**  VEGF **~** | Gao XM et al. (2019); doi.org/10.1007/s00395-019-0739-9^72^ |
| **Relaxin**  Receptor RXFP1, NO pathway | S.c injection of recombinant human relaxin-2 (serelaxin; 10 µg/kg) or saline (control), administered 1 h prior I/R (pretreatment) or administered 5 min prior to reperfusion (reperfusion therapy).  (Male C57BL mice; N varied per experiment [3-9 per group]) | Heart tissue sections (TTC and Phthalo blue staining) at 24 hours post-MI:  **Pretreatment:**  Infarct size **↘**  **Reperfusion therapy:**  Infarct size **↘** | Cardiac function (by ECG) 24 hours post-MI:  **Pretreatment:**  LVFS **↗**  LVESD **↘**  **Reperfusion therapy:**  LVFS **↗**  LVESD **↘** | Inflammation 24 hours post-MI:  **Reperfusion therapy**  Caspase-1 **↘**  **Pretreatment and reperfusion therapy:**  Mortality **↘** | Valle Raleigh J et al. (2017); doi.org/10.1093/cvr/cvw246^73^ |
| **Vascular endothelial growth factor B (VEGF-B)** PI3K/Akt, p38 MAPK, ERK1/2 signalling pathways | Myocardial injection of VEGF-B solution 200–300 µl (1.0 or 10 µg/mL)  into four sites (50 µl per site; two in the myocardium bordering the ischaemic area, and two within the ischaemic area) post-MI for 24 hours of reperfusion; MIRI alone (control). (Male Sprague Dawley rats; N = 6 per group) | Heart tissue sections (TTC staining) 24 hours post-reperfusion:  Infarct size **↘** | Cardiac function (via pressure transducer with catheterisation) after 24 hours of reperfusion:  LVSP **↗**  +d*P*/dt_max_ **↗**  -d*P*/dt_max_ **↗**  LVEDP **↘** | Myocardial injury (24 hours after reperfusion), and cardiac stem cell mobilisation (7 days post-reperfusion):  Cardiomyocytes apoptosis **↘**  Angiogenesis **↗**  SDF-1α and HGF expression **↗** | Li G et al. (2016); doi.org/10.1007/s00726-021-03005-8^74^ |

**↗ =** Increase in a parameter; **↘ =** decrease in a parameter**; ~** = No significant change in a parameter. Changes in a parameter are relative to controls included in each study (e.g. untreated MI). Colours of arrow and tilde symbols denote a positive effect (**green**), negative effect (**red**), and neutral effect (**orange**) of a protein-based therapy regarding limiting damage and/or mediating cardiac repair when administered post-MI.

+d*P*/dt_max_, maximal rate of increase in left ventricular pressure; -dP/dt_max_, maximal rate of decrease in left ventricular pressure; Akt, protein kinase B; APJ, apelin peptide jejunum or apelin-angiotensin receptor-like 1; AMI, acute myocardial infarction; atria/TL, atrial-to-tibial-length ratio; Aws, anterior wall systolic thickness; cTnT, cardiac troponin T; DBP, diastolic blood pressure; dLVP, developed left ventricular pressure; ECG, electrocardiogram; ELISA, enzyme-linked immunosorbent assay; ERK, extracellular signal-regulated kinase; GalR, galanin receptor; HE, haematoxylin and eosin; HGF, hepatocyte growth factor; HR, heart rate; HW/BW, heart-weight-to-body-weight ratio; IL, interleukin; i.p., intraperitoneal; IVSd, intraventricular septum thickness in diastole; IVSs, intraventricular septum thickness in systole; Lung/TL, lung-to-tibial-length ratio; LV/TL, left ventricular weight-to-tibial length ratio; LVAWDT, left ventricular anterior wall end-diastolic thickness; LVAWST, left ventricular anterior wall systolic thickness; LVDS, left ventricular diastolic septum thickness; LVEDA, left ventricular end-diastolic area; LVEDD, left ventricular end-diastolic diameter; LVEDP, left ventricular end-diastolic pressure; LVEDV, left ventricular end-diastolic volume; LVEF, left ventricular ejection fraction; LVESA, left ventricular end-systolic area; LVESD, left ventricular end-systolic diameter; LVESV, left ventricular end-systolic volume; LVFS, left ventricular fractional shortening; LVSP, left ventricular systolic pressure; LVW, left ventricular free wall weight; LVW/BW, left ventricular free wall weight-to-body weight ratio; MAP, mean arterial pressure; MAPK, mitogen-activated protein kinase; MI, myocardial infarction; MIRI, myocardial ischemia–reperfusion injury; MMP, matrix metalloproteinase; ND, not disclosed; NF-κB, nuclear factor kappa B; NO, nitric oxide; PAK, p21 -activated kinase; PBS, phosphate-buffered saline; PI3K, phosphatidylinositol 3-kinase; PRSW, preload-recruitable stroke work; RPP, rate pressure product; RT-PCR, reverse transcription polymerase chain reaction; RV/TL, right ventricular weight-to-tibial length ratio; RVW/BW, right ventricular weight-to-body weight ratio; RXFP, relaxin family peptide receptor; SAP, systolic arterial pressure; SBP, systolic blood pressure; SDF-1α, stromal cell-derived factor 1 alpha; SOD2, Superoxide dismutase 2; STAT3, signal transducer and activator of transcription 3; TGF-*β,* transforming growth factor *β*; TL, tibial length; TLR, toll-like receptor; TNF, tumour necrosis factor; TnI, troponin I; TTC, triphenyl tetrazolium chloride; TUNEL, Terminal deoxynucleotidyl transferase dUTP nick end labelling; VE, vascular endothelial; VEGF, vascular endothelial growth factor; WBC, white blood cell.

**Table S11.** *In vivo* large animal models of ischaemia/non-reperfusion and ischaemia/reperfusion injury (n = 9) investigating protein-based therapies that demonstrate an ability to limit damage and/or mediate cardiac repair when administered post-MI

| **Proteins, in alphabetical order** | **Administration method, timeline (when/for how long administered after MI), dosage(s)** | **Histomorphological endpoints with timepoint(s) when assessed after MI** | **Functional outcome, method (e.g. echo, MRI) with timepoints when assessed after MI** | **Other outcomes** | **Reference for protein therapy study** |
| --- | --- | --- | --- | --- | --- |
| **Agrin** Receptor Dag1, on cardiomyocytes | Single antegrade infusion of 33 µg/kg recombinant human agrin or saline (control) immediately after reperfusion with or without a second dose 3 days post-MI. (German pigs; single dose N varied per experiment) | Heart sections with TTC, Sirius Red, or wheat germ agglutinin staining at 28 days post MI (agrin vs control):  Infarct size **~**  HW/BW **↘**  Fibrosis **↘**  Myocyte size **↘** | MRI at 25 days post MI (agrin vs control):  Stroke volume **↗**  (For one agrin dose)  +d*P*/dt_max_ **~**  -d*P*/dt_min_ **~**  Myocardial contractility **↗** (trend observed)  Fluoroscopy at 28 days post MI (agrin vs control):  LVEF **↗**  PV-loop system at 28 days post MI (agrin vs control):  LVEDP **↘** | Repair mechanisms in infarct areas at day 3 (post MI followed by a 3-day reperfusion period with agrin vs saline treatment):  Activated macrophage (CD68⁺ and CD14⁺) **↘**  Myocardial cell proliferation (Ki67⁺) **↗**  Apoptotic cell death (TUNEL⁺) **↘** | Baehr A et al. (2020); doi.org/10.1161/CIRCULATIONAHA.119.045116^2^ |
| **Erythropoietin (EPO)**  Endothelial progenitor cells mobilisation | IV injection of recombinant human EPO (1,000 IU/kg; 5 ml) or saline (control) administered immediately (0h) post-MI or delayed after 6h or 1 week post MI. (Beagle dogs; 0h [n = 6–8], 6h post-MI [n = 8], or 1-week post-MI [n = 7]) | Myocardial tissue (TTC staining) at 6 h and 4 weeks post-MI (EPO vs control):  Infarct size **↘**  (0h dose schedule)  Infarct size **~**  (6h and 1 week delayed dose schedule) | Haemodynamic measurements (by pressure transducer via pigtail catheterisation and sidearm sheath) immediately post-MI and at 90 min and 4 weeks post-MI:  LVEDP **↘**  (0- and 6-hour dose schedules)  ABP **~**  HR **~**  Cardiac function by ECG immediately post-MI and at 90 min, 1 week, and 4 weeks post-MI:  LVEF **↗** (highest with 0h dose schedule)  LVEDD **↘**  (0- and 6-hour dose schedules)  Myocardial blood flow at 6h, 90 min and 4 weeks post-MI:  Myocardial blood flow **↗** (0- and 6-hour dose schedules at 4 weeks post MI) | Haematological parameters immediately post-MI and at 1 week, 2 weeks, and 4 weeks post-MI:  WBCs **~**  Platelets **~**  Haematocrit **~**  Plasma VEGF levels immediately post-MI and at 6h, 1 week and 2 weeks post-MI:  VEGF **~** (no difference between groups; significantly increased at 6 h post MI and returned to baseline at 1 week post MI) | Hirata A et al. (2006); doi.org/10.1016/j.jacc.2006.04.008^75^ |
| **Erythropoietin (EPO)**  PI3K/Akt, ERK signalling pathways | Four s.c. injections of recombinant human EPO (Epoetin 6000 U) or saline (control) at 2-day intervals post-MI.  (Female domestic pigs; N = 6) | Heart tissue sections (Azan staining) 28 days post-MI:  Infarct size **~**  LV wall thickness **~** | Cardiac function (by left ventriculography and coronary angiography) 28 days post-MI:  LVEDV **~**  LVEF **~** | Angiogenesis 1-, 4-, 6-, and 28-days post-MI:  Serum VEGF **~**  Serum HGF **↗** (1 day post MI)  Serum FGF **↗** (1 day post MI)  α-smooth muscle cell actin- and von Willebrand factor-stained sections **↗**  (At 28-days post-MI, border area)  Mobilisation of bone marrow progenitor cells 28 days post-MI:  Mobilisation of bone marrow progenitor cells **↗**  (In the border and infarcted areas) | Kawahchi K et al. (2012); doi.org/10.1007/s00380-011-0197-2^76^ |
| **Granulocyte colony-stimulating factor (G-CSF)** Jak-Stat, Akt/VEGF signalling pathways | Intramuscular injection of human recombinant G-CSF (10 µg/kg/day) administered immediately after reperfusion and every other day for 20 days (Early group, Male Yucatan minipig; n = 17) or administered every day for 10 days starting 5 days after MI (Delayed group; n = 8); MI + reperfusion alone (control) | Cardiac structure/remodelling (Sirius Red for type I and type III collagen; Movat Pentachrome for muscular arteries; Lectin and Factor 8 for capillaries; Smooth Muscle Actin for arterioles; Mac-4911 for macrophages; Trichrome for fibrosis and scarring; HE for nuclear and general tissue staining) at 5 days post-MI; fibrosis, 8 weeks post-MI:  **Early and delayed dose groups:**  Noninfarct weight/LVW **~**  Fibrosis **~**  Infarct weight/LVW **↗**  Infarct expansion **↗**  (At 8 weeks post-MI) | Cardiac function (by cardiac MRI) 5 days and 8 weeks post-MI:  **Early dose group:**  LVEDV **↘**  LVESV **~**  LVEF **~**  **Delayed dose group:**  LVEDV **~**  LVESV **~**  LVEF **↘** | Angiogenesis, and macrophage density 8 weeks post-MI:  **Early and Delayed dose groups:**  Angiogenesis **↘** (in peri-infarct border zone)  Inflammation  (macrophage density) **~** | Beohar N et al. (2007); doi.org/10.1002/ccd.20925^77^ |
| **Insulin-like growth factor-1 (IGF-1)** PI3K/Akt signalling pathway, ERK, GSK-3b | Intracoronary administration of recombinant human IGF-1 (50 pg/mL) or saline (control) post-MI/reperfusion.  (Female Landrace pigs; N = 17) | Heart transverse sections (TTC and methylene blue staining) at 2 months post-MI:  Infarct-related wall motion **↗**  Infarct related wall thickening **↗**  Thinning ratio **↗**  Infarct size **↘**  Expansion index **↘**  Fibrosis **↘** | Cardiac function (CT imaging and haemodynamic measurements via pressure-volume loops and catheterisation) at 2 months post-MI:  LVEF **↗**  +d*P*/dt **↗**  -d*P*/dt **↗**  LVEDV **↘**  LVESV **↘**  Ventricular fibrillation ~ | Apoptosis, inflammation, and cell-proliferation 24h post-MI:  Myocardial cell death **↘**  Cell proliferation **~**  Mortality ~  Inflammatory markers (in serum or infarct zone) **~** | O'Sullivan JF et al. (2011); doi.org/10.1161/CIRCINTERVENTIONS.110.960765^78^ |
| **Platelet-derived growth factor (PDGF)** cFib | Systemic infusion of 65 µg/kg recombinant human PDGF-AB (osmotic minipump) or untreated (control) delivered for 7 days prior to recovery after MI-reperfusion.  (Female Landrace swine; N varied per experiment) | Heart tissue sections (picrosirius red with fast green staining):  Fibrosis 11- and 28-days post-MI:  Fibrosis **↘**  Scar remodelling and maturation 11 days post-MI:  Scar anisotropy **↗**  Scar maturation **↗** | Cardiac function (by cardiac MRI) 9 days post-MI:  LVEF **~** | Inflammation (immunohistochemistry) 11 days post-MI:  Inflammation **↘** | Hume RD et al. (2023); doi.org/10.1016/j.jacbts.2022.11.006^79^ |
| **Platelet-derived growth factor (PDGF)** cFib | IV infusion of 65 µg/kg recombinant human PDGF-AB or vehicle (control) immediately after reperfusion via osmotic minipump, with repeat doses daily for 7 days.  (Female Landrace swine; N = 11) | Infarct size (by late gadolinium enhancement on cardiac MRI) 2 days post-MI:  Infarct size **~**  Fibrosis (myocardial sections with Gӧmӧri trichrome staining) 28-days post-MI:  Fibrosis **~**  Scar anisotropy **↗** | Cardiac function (by cardiac MRI) at 2- and 28-days post-MI:  LVEF **↗**  LVESV **↘**  (At 28 days post-MI)  LVEDV **~**  Cardiac function (haemodynamic measurements via micromanometer conductance catheterisation) 28-days post-MI:  d*P*/dt_min_ **↗**  d*P*/dt_max_ **↗**  ESPVR **↗**  PRSW **↗**  EDPVR **~**  𝜏 **~**  Arrythmia **↘** (At 28 days post-MI) | Angiogenesis and mortality 28-days post-MI:  Angiogenesis **↗**  Mortality **↘** | Thavapalachandran S et al. (2020); doi.org/10.1126/scitranslmed.aay2140^80^ |
| **Tissue inhibitor of matrix metalloproteinase-3 (TIMP-3)** MMP | Myocardial injection of full-length recombinant TIMP-3 or a truncated form of TIMP-3, encompassing the N-terminal region (N-TIMP-3), or saline (control), post-MI. For both, 30 mg total injection across 6 injection sites.  Male Yorkshire pigs: N = 8 (TIMP-3); N = 9 (N-TIMP-3) | LV sections (HE and picrosirius red staining) 14 days post-MI:  **TIMP and N-TIMP:**  Fibrosis **~**  **TIMP:**  Left atrial area **↘**  (At 3-, 7- and 14-days post-MI)  **N-TIMP:**  Left atrial area **↘**  (At 3- and 14-days post-MI) | Cardiac function (by ECG) and pulmonary capillary wedge pressure 1-, 3-, 7- and 14-days post-MI:  **TIMP and N-TIMP:**  LVPWd **↗**  (At 3-, 7- and 14-days post-MI)  LVEF **↗**;  Pulmonary capillary wedge pressure **↘**  **TIMP:**  LVEDV **↘**  **N-TIMP:**  LVEDV **↘**  (At 14-days post-MI) | Myocardial damage at 24 and 72 hours and 14 days post-MI:  **TIMP and N-TIMP:**  Myocardial damage **~**  (Plasma TnI)  Inflammation, apoptosis, macrophage density, markers of cardiac remodelling 14 days post-MI:  **TIMP and N-TIMP:**  Macrophage density **~**  **TIMP:**  Inflammation **↘**;  Apoptosis **↘**;  Markers of cardiac remodelling (MMP-2, MMP-9, and MMP-14) **↘**  **N-TIMP:**  Inflammation **~**;  Apoptosis **~**;  Markers of cardiac remodelling (MMP-2, MMP-9, and MMP-14) **~** | Lobb DC et al. (2020); doi.org/10.1124/jpet.120.000047^81^ |
| **Tissue inhibitor of matrix metalloproteinase-3 (TIMP-3)** MMP | Intracoronary infusion of recombinant TIMP-3 (30 mg within a 3 mL volume) or saline (control) at the final 4 min of an ischaemic period, prior to reperfusion.  (Male Yorkshire pigs; N = 9) | Infarct size, fibrosis in the MI and remote regions, cardiomyocyte size (LV sections with picro-sirius red and TTC staining) 28 days post-MI:  Infarct size **↘**  Fibrosis **↘**  (In the MI region) | Cardiac function (by ECG) 3, 7 14-, and 28-days post-MI:  LVPWd ↗  (At 7 days post-MI)  Fractional shortening **↗**  (At 28 days post-MI)  LVEDV **↘**  (At 14- and 28-days post-MI)  LVEF 28 days post-MI:  LVEF **↗** | Myocardial damage 90 mins, 100 mins, 110 mins, 120 mins and 28 days post-MI:  Myocardial damage **~**  (Plasma TnI)  Plasma NT-ProBNP concentration, inflammation, and apoptosis 28 days post-MI:  Hypertrophic/HF marker(s) (NT-ProBNP) **↘**  Inflammation **~**  Apoptosis **~** | Barlow SC et al. (2017); doi.org/10.1152/ajpheart.00114.2017^82^ |

**↗ =** Increase in a parameter; **↘ =** decrease in a parameter**; ~** = No significant change in a parameter. Changes in a parameter are relative to controls included in each study (e.g. untreated MI). Colours of arrow and tilde symbols denote a positive effect (**green**), negative effect (**red**), and neutral effect (**orange**) of a protein-based therapy regarding limiting damage and/or mediating cardiac repair when administered post-MI.

Akt, protein kinase B; +d*P*/dt_max_, maximal rate of increase in left ventricular pressure; -dP/dt_max_, maximal rate of decrease in left ventricular pressure; ABP, arterial mean blood pressure; BNP, brain natriuretic peptide; cFIB, cardiac fibroblast; Dag1, alpha-dystroglycan; ECG, electrocardiogram; EDPVR, end-diastolic pressure-volume relationship; ERK, extracellular signal-regulated kinase; ESPVR, end-systolic pressure-volume relationship; HE, haematoxylin and eosin; HF, heart failure; HR, heart rate; HW/BW, heart-weight-to-body-weight ratio; i.p., intraperitoneal; IV, intravenous; LV, left ventricular; LVEDP, left ventricular end-diastolic pressure; LVEDV, left ventricular end-diastolic volume; LVEDD, left ventricular end-diastolic diameter; LVEF, left ventricular ejection fraction; LVESV, left ventricular end-systolic volume; LVMV, left ventricular myocardial volume; LVSP, left ventricular systolic pressure; LVPWd, left ventricular posterior wall thickness in diastole; MAPK, mitogen-activated protein kinase; MI, myocardial infarction; MMP, matrix metalloproteinase; PI3K, phosphatidylinositol 3-kinase; PRSW, preload-recruitable stroke work; PWd, posterior wall thickness in diastole; s.c., subcutaneous; TIMP, tissue inhibitor of metalloproteinases; TnI, troponin I; TTC, triphenyl tetrazolium chloride; TUNEL, Terminal deoxynucleotidyl transferase dUTP nick end labelling; VEGF, vascular endothelial growth factor.

**Table S12.** Protein-based therapies with highest potential efficacy for myocardial repair and protection (highest ranking scores based on improvement in functional, structural, cellular, and survival outcomes of protein-based therapies assessed in more than one study)

| **Protein** | **Pathway** | **Number of studies** | **MI model** | **Cardiac function outcomes** | **Structural outcomes** | **Cellular/molecular outcomes** | **Survival** | **Score** |
| --- | --- | --- | --- | --- | --- | --- | --- | --- |
| Erythropoietin | PI3K/Akt, JAK/STAT, MAPK, ERK, TGF | 12 | - Small animal models of ischaemia/non-reperfusion - Small animal models of I/R - Large animal models | - Improvement of LVEF and/or LVFS in 9/12 studies - No change in 3/12 studies | - IS and/or fibrosis reduction in 10/12 studies - No change in 2/12 studies | - Improvement in 11/12 studies - No change in 1/12 studies | - Improved survival in 1/12 studies - No change in 4/12 studies - Not reported in 7/12 studies | 13.4 |
| Apelin-13/apela | PI3K/Akt  MAPK, ERK,  VEGF  TGF-β  NF-κB | 6 | - Small animal models of ischaemia/non-reperfusion - Small animal models of I/R | - Improvement of LVEF and/or LVFS in 6/6 studies | - IS and/or fibrosis reduction in 6/6 studies | - Improvement in 6/6 studies | - No change in 1/6 studies - Not reported in 5/6 studies | 13.0 |
| G-CSF | JAK-STAT,  Akt/VEGF | 4 | - Small animal models of ischaemia/non-reperfusion - Small animal models of I/R - Large animal models | - Improvement of LVEF and/or LVFS in 3/4 studies - No change in 1/4 studies | - IS and/or fibrosis reduction in 2/4 studies - No change in 2/4 studies | - Improvement in 3/4 studies - Reduction in angiogenesis in 1/4 studies (large animal) | - Improved survival in 2/4 studies; no change in 1/4 studies; not reported in 1/4 studies | 12.7 |
| MYDGF | PI3K-Akt,  MAPK-STAT3, c-Myc/FoxM1 | 2 | - Small animal models of ischaemia/non-reperfusion - Small animal models of I/R | - Improvement of LVEF and/or LVFS in 2/2 studies | - IS and/or fibrosis reduction in 2/2 studies | - Improvement in 2/2 studies | - Improved survival in 2/2 studies | 12.0 |
| IL-37 | TLR-4,  NF-κB, JAK/STAT | 2 | - Small animal models of ischaemia/non-reperfusion - Small animal models of I/R | - Improvement of LVEF and/or LVFS in 2/2 studies | - IS and/or fibrosis reduction in 2/2 studies | - Improvement in 2/2 studies | - Improved survival in 1/2 studies; not reported in 1/2 studies | 11.5 |
| Alpha-1-antitrypsin (AAT)/prolastin C | Caspase-1 | 3 | - Small animal models of ischaemia/non-reperfusion - Small animal models of I/R | - Improvement of LVEF and/or LVFS in 3/3 studies | - IS and/or fibrosis reduction in 2/3 studies - No change in 1/3 studies | - Improvement in 3/3 studies | - Not reported | 11.0 |
| Gastrin | PI3K/Akt/VEGF, HIF-1α/VEGF | 2 | - Small animal models of ischaemia/non-reperfusion | - Improvement of LVEF and/or LVFS in 2/2 studies | - IS and/or fibrosis reduction in 2/2 studies | - Improvement in 2/2 studies | - Improved survival in 2/2 studies | 11.0 |
| Irisin | ERK, SOD2 | 2 | - Small animal models of ischaemia/non-reperfusion - Small animal models of I/R | - Improvement of LVEF and/or LVFS in 2/2 studies | - IS and/or fibrosis reduction in 2/2 studies | - Improvement in 2/2 studies | - Not reported | 11.0 |

AAT, alpha-1 antitrypsin; Akt, protein kinase B; G-CSF, granulocyte colony-stimulating factor; ERK, extracellular signal-regulated kinase; HIF-1α, hypoxia-inducible factor 1-alpha; IL-37, interleukin 37; I/R, ischaemia-reperfusion; IS, infarct size; JAK/STAT, signal transducer and activator of transcription; LVEF, left ventricular ejection fraction; LVFS, left ventricular fractional shortening; MAPK, mitogen-activated protein kinase; MI, myocardial infarction; MYDGF, myeloid-derived growth factor; NF-κB, nuclear factor kappa B; PI3K, phosphatidylinositol 3-kinase; SOD2, superoxide dismutase 2; TLR-4, toll-like receptor 4; TGF-*β,* transforming growth factor *β*; VEGF, vascular endothelial growth factor.

**Table S13.** Summary of clinical trials investigating modified proteins in the STEMI setting

| Protein-based therapy | Study | Phase | N | Intervention | Primary outcomes | Secondary outcomes | Reference |
| --- | --- | --- | --- | --- | --- | --- | --- |
| Recombinant human EPO (epoetin-β) | REVIVAL-3 NCT00390832 | 3 | 138 | rhEPO at 0h, 24h, and 48h after PCI or placebo | LVEF at 6 months by MRI: 52.0 ± 9.1% (rhEPO) vs 51.8 ± 9.3% (placebo) (P = 0.92) | Infarct size at 5 days (post randomisation): 26.8 ± 20.9% vs 28.3 ± 24.4% (P = 0.76)  Infarct size at 6 months: 17.3 ± 14.3% vs 20.9 ± 16.4% (P = 0.27)  Cumulative 6-month incidence of death, recurrent MI, stroke, or target vessel revascularisation: 13.2% vs 5.7% (HR 2.36; 95% CI: 0.73, 7.66; P = 0.15) | Ott I et al. 2010^83^ |
| Recombinant human EPO (epoetin-β) | REVIVAL-3 (Five-year results) NCT00390832 | 3 | 138 | rhEPO at 0h, 24h, and 48h after PCI or placebo | Combined incidence of MACE 5 years after randomisation: 25% (rhEPO) vs 17% (placebo); RR 1.5; 95% CI 0.8, 3.5; P = 0.26 | Death or MI or stroke in 10 patients (14.7%, rhEPO) vs 7 patients (10.0%, placebo) (P = 0.40)  Target lesion revascularisation required in 15 patients (22.1%, rhEPO) vs 9 patients (12.9%, placebo) (P = 0.15) | Steppich B et al. 2017^84^ |
| Recombinant human EPO (epoetin-α) | REVEAL NCT00378352 | 2 | 222 | Epoetin-α or matching saline placebo administered within 4 hours of reperfusion | Infarct size by CMR at 2–6 days (post administration): 15.8% LV mass (95% CI: 13.3, 18.2) (rhEPO) vs 15.0% LV mass (95% CI: 12.6, 17.3%) (placebo) (P = 0.67)  Infarct size by CMR at 12 ± 2 weeks later: 10.6% LV mass (95% CI: 8.4, 12.8%) vs 10.4% LV mass (95% CI: 8.5, 12.3%) (P = 0.89) | LVEF by CMR at 2–6 days: 48.2% (95% CI: 46.0, 50.4%) (rhEPO) vs 48.9% (95% CI: 46.8, 50.9%) (placebo) (P = 0.67)  LVEF by CMR at 12 ± 2 weeks later: 52.5% (95% CI: 50.2, 54.8%) vs 52.0% (95% CI: 49.8, 54.2%) (P = 0.76)  Composite outcome of death, MI, stroke, or stent thrombosis: n = 5 (4.0%; 95% CI: 1.31, 9.09%) vs 0 (P = .04) | Najjar SS et al. 2011^85^ |
| Fibrin-derived peptide Bβ15-42 (FXO6) | F.I.R.E NCT00326976 | 2 | 234 | FXO6 or placebo, at reperfusion | Infarct size by cMRI (LGE zone) at 5 days (post intervention): 21.7 g (IQ range: 8.3, 47.1) vs 27.3g (IQ range: 11.7, 44.9) (21% reduction, P = 0.207) | Necrotic core zone at 5 days: 1.77 g (IQ range: 0, 9.09) vs 4.20 g (IQ range: 0.3, 9.93) (58% reduction, P = 0.019)  LVEF at 5 days: 47.2% (IQ range: 40.3, 55.4) vs 46.8% (IQ range: 41.2, 53.0) (0.8% increase, P = 0.455) | Atar D et al. 2009^86^ |
| G-CSF | REVIVAL-2 NCT00126100 | 4 | 114 | G-CSF or placebo for 5 days | Change from baseline in LV infarct size by scintigraphy at 4–6 months (post randomisation): –6.2% vs –4.9% (P = 0.56) | Change from baseline in LVEF by MRI at 4–6 months (post randomisation):  +0.5% vs +2% (P = 0.14)  Angiographic restenosis occurred in 19 35.2% (19/54; G-CSF) vs 30.9% (17/55; placebo) (P = 0.79) | Zohlnhöfer D et al. 2006^87^ |
| G-CSF | STEM-AMI Eudra-CT Code 2005-004706-90 | 2 | 60 | G-CSF or placebo for 5 days, starting <12 h after PCI | Increase from baseline to 6 months in LVEF by ECG: 39.4 to 46.2 %, P = 0.016 (G-CSF) and 39.6 to 42.8 %, P = 0.170 (placebo) | Change in LVEDV (from baseline to 6 months) by ECG: 48.7 to 60.8 mL/m^2^, P = 0.0.024 (G-CSF) and 48.5 to 68.8 mL/m^2^, P = 0.002 (placebo)  Change in LVESV (from baseline to 6 months) by ECG: 28.3 to 34.0 mL/m^2^, P = 0.144 (G-CSF) and 29.0 to 41.6 mL/m^2^, P = 0.010 (placebo) | Achilli F et al. 2010^88^ |
| G-CSF | STEM-AMI OUTCOME CRM substudy NCT01969890 | 3 | 161 | SoC + G-CSF or SoC | Change in LVEF from baseline to 6 months: 4.75% (95% CI: 1.99, 7.51; G-CSF) vs –0.35% (95% CI: –3.18, 2.49; placebo) (P = 0.0142) | Change in LVEDVI from baseline to 6 months: –0.63 mL/m^2^ (95% CI: –4.59, 3.33; G-CSF) vs 1.98 mL/m^2^ (95% CI: –2.08, 6.05; placebo) (P = 0.3747)  Change in LVESVI from baseline to 6 months: –4.12 mL/m^2^ (95% CI: –7.94, –0.29; G-CSF) vs 2.58 mL/m^2^ (95% CI: –1.35, 6.51; placebo) (P = 0.0198)  Change in infarct size from baseline to 6 months: –3.10% (95% CI: –5.48, –0.73; G-CSF) vs –1.50% (95% CI: –4.09, 1.10; placebo) (P = 0.3755) | Achilli F et al. 2019^89^ |
| G-CSF | STEM-AMI OUTCOME *post hoc* analysis NCT01969890 | 3 | 521 | SoC + G-CSF or SoC | All-cause death, recurrence of MI, and hospitalisation for heart failure: no difference between treatment groups (HR 1.20; 95% CI 0.63, 2.28) | Trend towards fewer composite primary outcomes in patients with low bone marrow cell mobilisation (n = 108) vs those with high mobilisation (n = 152, with peak leucocyte count >50×10^9^/L; HR 2.86; 95% CI 0.96, 8.56)  Primary outcomes were lower in patients with severe LV systolic dysfunction at discharge treated with G-CSF vs control (interaction β ± SE, −0.08 ± 0.04; P = 0.034) | Achilli F et al. 2024^90^ |
| Prolastin C | VCU-Alpha 1-RT NCT01936896 | 1/2 | 10 | Prolastin C or historical controls | Lower area under the curve of CRP levels 14 days after admission in the prolastin C group (75.9 vs 205.6 mg/L, P = 0.048) |  | Abbate A et al. 2015^91^ |

CI, confidence interval; CMR, cardiac magnetic resonance; cMRI, cardiac magnetic resonance imaging; CRP, C-reactive protein; ECG, electrocardiography; EPO, erythropoietin; G-CSF, granulocyte colony-stimulating factor; HR, hazard ratio; IQ, interquartile; LGE, late gadolinium enhancement; LV, left ventricular; LVEDV, left ventricular end-diastolic volume; LVEDVI, left ventricular end-diastolic volume indexed; LVEF, left ventricular ejection fraction; LVESV, left ventricular end-systolic volume; LVESVI, left ventricular end-systolic volume indexed; MACE, major adverse cardiac events; MI, myocardial infarction; MRI, magnetic resonance imaging; PCI, primary coronary intervention; prolastin C, plasma-derived alpha-1 antitrypsin; rhEPO, recombinant human erythropoietin; RR, relative risk; SE, standard error; SoC, standard of care.

# References

1. Adini A, Adini I, Grad E, Tal Y, Danenberg HD, Kang PM, Matthews BD, D'Amato RJ. The prominin-1-derived peptide improves cardiac function following ischemia. *Int J Mol Sci* 2021;**22**:5169.

2. Baehr A, Umansky KB, Bassat E, Jurisch V, Klett K, Bozoglu T, Hornaschewitz N, Solyanik O, Kain D, Ferraro B, Cohen-Rabi R, Krane M, Cyran C, Soehnlein O, Laugwitz KL, Hinkel R, Kupatt C, Tzahor E. Agrin Promotes Coordinated Therapeutic Processes Leading to Improved Cardiac Repair in Pigs. *Circulation* 2020;**142**:868-881.

3. Li J, Ding H, Li Y, Zhou H, Wang W, Mei Y, Zhang R. Alarin alleviated cardiac fibrosis via attenuating oxidative stress in heart failure rats. *Amino Acids* 2021;**53**:1079-1089.

4. Toldo S, Seropian IM, Mezzaroma E, Van Tassell BW, Salloum FN, Lewis EC, Voelkel N, Dinarello CA, Abbate A. Alpha-1 antitrypsin inhibits caspase-1 and protects from acute myocardial ischemia-reperfusion injury. *Journal of molecular and cellular cardiology* 2011;**51**:244-251.

5. Cho DI, Kang HJ, Jeon JH, Eom GH, Cho HH, Kim MR, Cho M, Jeong HY, Cho HC, Hong MH, Kim YS, Ahn Y. Antiinflammatory activity of ANGPTL4 facilitates macrophage polarization to induce cardiac repair. *JCI Insight* 2019;**4**.

6. Ferraro B, Leoni G, Hinkel R, Ormanns S, Paulin N, Ortega-Gomez A, Viola JR, de Jong R, Bongiovanni D, Bozoglu T, Maas SL, D'Amico M, Kessler T, Zeller T, Hristov M, Reutelingsperger C, Sager HB, Doring Y, Nahrendorf M, Kupatt C, Soehnlein O. Pro-angiogenic macrophage phenotype to promote myocardial repair. *J Am Coll Cardiol* 2019;**73**:2990-3002.

7. Pan Y, Li Q, Yan H, Huang J, Wang Z. Apela improves cardiac and renal function in mice with acute myocardial infarction. *J Cell Mol Med* 2020;**24**:10382-10390.

8. Zhang X, Hu W, Feng F, Xu J, Wu F. Apelin-13 protects against myocardial infarction-induced myocardial fibrosis. *Mol Med Rep* 2016;**13**:5262-5268.

9. Zhang NK, Cao Y, Zhu ZM, Zheng N, Wang L, Xu XH, Gao LR. Activation of Endogenous Cardiac Stem Cells by Apelin-13 in Infarcted Rat Heart. *Cell Transplant* 2016;**25**:1645-1652.

10. Zhong S, Guo H, Wang H, Xing D, Lu T, Yang J, Wang C. Apelin-13 alleviated cardiac fibrosis via inhibiting the PI3K/Akt pathway to attenuate oxidative stress in rats with myocardial infarction-induced heart failure. *Biosci Rep* 2020;**40**.

11. Jin Y, Cheng X, Lu J, Li X. Exogenous BMP-7 Facilitates the Recovery of Cardiac Function after Acute Myocardial Infarction through Counteracting TGF-β1 Signaling Pathway. *The Tohoku journal of experimental medicine* 2018;**244**:1-6.

12. Sugiyama A, Ito R, Okada M, Yamawaki H. Long-term administration of recombinant canstatin prevents adverse cardiac remodeling after myocardial infarction. *Sci Rep* 2020;**10**:12881.

13. Wang C, Zhang C, Wu D, Guo L, Zhao F, Lv J, Fu L. Cholecystokinin octapeptide reduces myocardial fibrosis and improves cardiac remodeling in post myocardial infarction rats. *Int J Biochem Cell Biol* 2020;**125**:105793.

14. Moon C, Krawczyk M, Ahn D, Ahmet I, Paik D, Lakatta EG, Talan MI. Erythropoietin reduces myocardial infarction and left ventricular functional decline after coronary artery ligation in rats. *Proc Natl Acad Sci U S A* 2003;**100**:11612-11617.

15. van der Meer P, Lipsic E, Henning RH, Boddeus K, van der Velden J, Voors AA, van Veldhuisen DJ, van Gilst WH, Schoemaker RG. Erythropoietin induces neovascularization and improves cardiac function in rats with heart failure after myocardial infarction. *J Am Coll Cardiol* 2005;**46**:125-133.

16. Zuo L, Li DD, Ma XX, Shi SH, Lyu DC, Shen J, Zhang WF, Gao EH, Cao JM. Erythropoietin promotes myocardial infarction repair in mice by improving the function of Sca-1(+) stem cells. *Sheng li xue bao : [Acta physiologica Sinica]* 2023;**75**:36-48.

17. Gabel R, Klopsch C, Furlani D, Yerebakan C, Li W, Ugurlucan M, Ma N, Steinhoff G. Single high-dose intramyocardial administration of erythropoietin promotes early intracardiac proliferation, proves safety and restores cardiac performance after myocardial infarction in rats. *Interact Cardiovasc Thorac Surg* 2009;**9**:20-25; discussion 25.

18. Klopsch C, Furlani D, Gabel R, Li W, Pittermann E, Ugurlucan M, Kundt G, Zingler C, Titze U, Wang W, Ong LL, Wagner K, Li RK, Ma N, Steinhoff G. Intracardiac injection of erythropoietin induces stem cell recruitment and improves cardiac functions in a rat myocardial infarction model. *J Cell Mol Med* 2009;**13**:664-679.

19. Klopsch C, Skorska A, Ludwig M, Lemcke H, Maass G, Gaebel R, Beyer M, Lux C, Toelk A, Müller K, Maschmeier C, Rohde S, Mela P, Müller-Hilke B, Jockenhoevel S, Vollmar B, Jaster R, David R, Steinhoff G. Intramyocardial angiogenetic stem cells and epicardial erythropoietin save the acute ischemic heart. *Disease models & mechanisms* 2018;**11**.

20. Moon C, Krawczyk M, Paik D, Lakatta EG, Talan MI. Cardioprotection by recombinant human erythropoietin following acute experimental myocardial infarction: dose response and therapeutic window. *Cardiovasc Drugs Ther* 2005;**19**:243-250.

21. Xi Y, Yu D, Yang R, Zhao Q, Wang J, Zhang H, Qian K, Shi Z, Wang W, Brown R, Li Y, Tian Z, Gong DW. Recombinant Fc-Elabela fusion protein has extended plasma half-life andmitigates post-infarct heart dysfunction in rats. *Int J Cardiol* 2019;**292**:180-187.

22. Fu J, Tang Y, Zhang Z, Tong L, Yue R, Cai L. Gastrin exerts a protective effect against myocardial infarction via promoting angiogenesis. *Mol Med* 2021;**27**:90.

23. Wang R, Zhang Z, Xu Z, Wang N, Yang D, Liu ZZ, Liao Q, Xia X, Chen C, Shou J, Li L, Wang WE, Zeng C, Xia T, Wang H. Gastrin mediates cardioprotection through angiogenesis after myocardial infarction by activating the HIF-1α/VEGF signalling pathway. *Scientific reports* 2021;**11**:15836.

24. Soeki T, Kishimoto I, Schwenke DO, Tokudome T, Horio T, Yoshida M, Hosoda H, Kangawa K. Ghrelin suppresses cardiac sympathetic activity and prevents early left ventricular remodeling in rats with myocardial infarction. *Am J Physiol Heart Circ Physiol* 2008;**294**:H426-432.

25. Huang CX, Yuan MJ, Huang H, Wu G, Liu Y, Yu SB, Li HT, Wang T. Ghrelin inhibits post-infarct myocardial remodeling and improves cardiac function through anti-inflammation effect. *Peptides* 2009;**30**:2286-2291.

26. Chen H, Liu Y, Gui Q, Zhu X, Zeng L, Meng J, Qing J, Gao L, Jackson AO, Feng J, Li Y, He J, Yin K. Ghrelin attenuates myocardial fibrosis after acute myocardial infarction via inhibiting endothelial-to mesenchymal transition in rat model. *Peptides* 2019;**111**:118-126.

27. Parry TJ, Ganguly A, Troy EL, Luis Guerrero J, Iaci JF, Srinivas M, Vecchione AM, Button DC, Hackett CS, Zolty R, Sawyer DB, Caggiano AO. Effects of neuregulin GGF2 (cimaglermin alfa) dose and treatment frequency on left ventricular function in rats following myocardial infarction. *Eur J Pharmacol* 2017;**796**:76-89.

28. Hill MF, Patel AV, Murphy A, Smith HM, Galindo CL, Pentassuglia L, Peng X, Lenneman CG, Odiete O, Friedman DB, Kronenberg MW, Zheng S, Zhao Z, Song Y, Harrell FE, Jr., Srinivas M, Ganguly A, Iaci J, Parry TJ, Caggiano AO, Sawyer DB. Intravenous glial growth factor 2 (GGF2) isoform of neuregulin-1β improves left ventricular function, gene and protein expression in rats after myocardial infarction. *PLoS One* 2013;**8**:e55741.

29. Harada M, Qin Y, Takano H, Minamino T, Zou Y, Toko H, Ohtsuka M, Matsuura K, Sano M, Nishi J, Iwanaga K, Akazawa H, Kunieda T, Zhu W, Hasegawa H, Kunisada K, Nagai T, Nakaya H, Yamauchi-Takihara K, Komuro I. G-CSF prevents cardiac remodeling after myocardial infarction by activating the Jak-Stat pathway in cardiomyocytes. *Nat Med* 2005;**11**:305-311.

30. Ohtsuka M, Takano H, Zou Y, Toko H, Akazawa H, Qin Y, Suzuki M, Hasegawa H, Nakaya H, Komuro I. Cytokine therapy prevents left ventricular remodeling and dysfunction after myocardial infarction through neovascularization. *FASEB J* 2004;**18**:851-853.

31. Grimm D, Cameron D, Griese DP, Riegger GA, Kromer EP. Differential effects of growth hormone on cardiomyocyte and extracellular matrix protein remodeling following experimental myocardial infarction. *Cardiovasc Res* 1998;**40**:297-306.

32. Wo D, Peng J, Ren DN, Qiu L, Chen J, Zhu Y, Yan Y, Yan H, Wu J, Ma E, Zhong TP, Chen Y, Liu Z, Liu S, Ao L, Liu Z, Jiang C, Peng J, Zou Y, Qian Q, Zhu W. Opposing roles of Wnt inhibitors IGFBP-4 and Dkk1 in cardiac ischemia by differential targeting of LRP5/6 and β-catenin. *Circulation* 2016;**134**:1991-2007.

33. Xu JY, Xiong YY, Tang RJ, Jiang WY, Ning Y, Gong ZT, Huang PS, Chen GH, Xu J, Wu CX, Hu MJ, Xu J, Xu Y, Huang CR, Jin C, Lu XT, Qian HY, Li XD, Yang YJ. Interleukin-5-induced eosinophil population improves cardiac function after myocardial infarction. *Cardiovasc Res* 2022;**118**:2165-2178.

34. Krishnamurthy P, Rajasingh J, Lambers E, Qin G, Losordo DW, Kishore R. IL-10 inhibits inflammation and attenuates left ventricular remodeling after myocardial infarction via activation of STAT3 and suppression of HuR. *Circulation research* 2009;**104**:e9-18.

35. An W, Yu Y, Zhang Y, Zhang Z, Yu Y, Zhao X. Exogenous IL-19 attenuates acute ischaemic injury and improves survival in male mice with myocardial infarction. *Br J Pharmacol* 2019;**176**:699-710.

36. Tang TT, Li YY, Li JJ, Wang K, Han Y, Dong WY, Zhu ZF, Xia N, Nie SF, Zhang M, Zeng ZP, Lv BJ, Jiao J, Liu H, Xian ZS, Yang XP, Hu Y, Liao YH, Wang Q, Tu X, Mallat Z, Huang Y, Shi GP, Cheng X. Liver-heart crosstalk controls IL-22 activity in cardiac protection after myocardial infarction. *Theranostics* 2018;**8**:4552-4562.

37. Zhu R, Sun H, Yu K, Zhong Y, Shi H, Wei Y, Su X, Xu W, Luo Q, Zhang F, Zhu Z, Meng K, Zhao X, Liu Y, Mao Y, Cheng P, Mao X, Zeng Q. Interleukin-37 and Dendritic Cells Treated With Interleukin-37 Plus Troponin I Ameliorate Cardiac Remodeling After Myocardial Infarction. *Journal of the American Heart Association* 2016;**5**.

38. Liao Q, Qu S, Tang LX, Li LP, He DF, Zeng CY, Wang WE. Irisin exerts a therapeutic effect against myocardial infarction via promoting angiogenesis. *Acta Pharmacol Sin* 2019;**40**:1314-1321.

39. Wang K, Li Z, Ding Y, Liu Z, Li Y, Liu X, Sun Y, Hong J, Zheng W, Qian L, Xu D. Klotho improves cardiac fibrosis, inflammatory cytokines, ferroptosis, and oxidative stress in mice with myocardial infarction. *Journal of Physiology and Biochemistry* 2023.

40. Wang K, Li Z, Li Y, Liu X, Sun Y, Hong J, Ding Y, Zheng W, Qian L, Xu D. Cardioprotection of Klotho against myocardial infarction-induced heart failure through inducing autophagy. *Mech Ageing Dev* 2022;**207**:111714.

41. Reboll MR, Klede S, Taft MH, Cai CL, Field LJ, Lavine KJ, Koenig AL, Fleischauer J, Meyer J, Schambach A, Niessen HW, Kosanke M, van den Heuvel J, Pich A, Bauersachs J, Wu X, Zheng L, Wang Y, Korf-Klingebiel M, Polten F, Wollert KC. Meteorin-like promotes heart repair through endothelial KIT receptor tyrosine kinase. *Science* 2022;**376**:1343-1347.

42. Wang Y, Li Y, Feng J, Liu W, Li Y, Liu J, Yin Q, Lian H, Liu L, Nie Y. Mydgf promotes Cardiomyocyte proliferation and Neonatal Heart regeneration. *Theranostics* 2020;**10**:9100-9112.

43. Liu X, Gu X, Li Z, Li X, Li H, Chang J, Chen P, Jin J, Xi B, Chen D, Lai D, Graham RM, Zhou M. Neuregulin-1/erbB-activation improves cardiac function and survival in models of ischemic, dilated, and viral cardiomyopathy. *J Am Coll Cardiol* 2006;**48**:1438-1447.

44. Bersell K, Arab S, Haring B, Kuhn B. Neuregulin1/ErbB4 signaling induces cardiomyocyte proliferation and repair of heart injury. *Cell* 2009;**138**:257-270.

45. Qin YY, Huang XR, Zhang J, Wu W, Chen J, Wan S, Yu XY, Lan HY. Neuropeptide Y attenuates cardiac remodeling and deterioration of function following myocardial infarction. *Mol Ther* 2022;**30**:881-897.

46. Lörchner H, Adrian-Segarra JM, Waechter C, Wagner R, Góes ME, Brachmann N, Sreenivasan K, Wietelmann A, Günther S, Doll N, Braun T, Pöling J. Concomitant Activation of OSM and LIF Receptor by a Dual-Specific hlOSM Variant Confers Cardioprotection after Myocardial Infarction in Mice. *International journal of molecular sciences* 2021;**23**.

47. Zhang X, Zhu D, Wei L, Zhao Z, Qi X, Li Z, Sun D. OSM enhances angiogenesis and improves cardiac function after myocardial infarction. *Biomed Res Int* 2015;**2015**:317905.

48. Rotem I, Konfino T, Caller T, Schary Y, Shaihov-Teper O, Palevski D, Lewis N, Lendengolts D, Naftali-Shani N, Leor J. Osteopontin promotes infarct repair. *Basic Res Cardiol* 2022;**117**:51.

49. Bock-Marquette I, Maar K, Maar S, Lippai B, Faskerti G, Gallyas F, Jr., Olson EN, Srivastava D. Thymosin beta-4 denotes new directions towards developing prosperous anti-aging regenerative therapies. *Int Immunopharmacol* 2023;**116**:109741.

50. Chintalgattu V, Greenberg J, Singh S, Chiueh V, Gilbert A, O'Neill JW, Smith S, Jackson S, Khakoo AY, Lee T. Utility of glycosylated TIMP3 molecules: inhibition of MMPs and TACE to improve cardiac function in rat myocardial infarct model. *Pharmacol Res Perspect* 2018;**6**:e00442.

51. Klotz L, Norman S, Vieira JM, Masters M, Rohling M, Dube KN, Bollini S, Matsuzaki F, Carr CA, Riley PR. Cardiac lymphatics are heterogeneous in origin and respond to injury. *Nature* 2015;**522**:62-67.

52. Ji M, Li Y, Liu Y, Ma G. Vaspin ameliorates cardiac remodeling by suppressing phosphoinositide 3-kinase/protein kinase B pathway to improve oxidative stress in heart failure rats. *J Cardiovasc Pharmacol* 2022;**80**:442-452.

53. Mauro AG, Mezzaroma E, Marchetti C, Narayan P, Del Buono MG, Capuano M, Prestamburgo A, Catapano S, Salloum FN, Abbate A, Toldo S. A preclinical translational study of the cardioprotective effects of plasma-derived alpha-1 anti-trypsin in acute myocardial infarction. *J Cardiovasc Pharmacol* 2017;**69**:273-278.

54. Azizi Y, Faghihi M, Imani A, Roghani M, Zekri A, Mobasheri MB, Rastgar T, Moghimian M. Post-infarct treatment with [Pyr(1)]apelin-13 improves myocardial function by increasing neovascularization and overexpression of angiogenic growth factors in rats. *European journal of pharmacology* 2015;**761**:101-108.

55. Tao J, Zhu W, Li Y, Xin P, Li J, Liu M, Li J, Redington AN, Wei M. Apelin-13 protects the heart against ischemia-reperfusion injury through inhibition of ER-dependent apoptotic pathways in a time-dependent fashion. *Am J Physiol Heart Circ Physiol* 2011;**301**:H1471-1486.

56. Birnbaum Y, Ye R, Chen H, Carlsson L, Whatling C, Fjellström O, Ryberg E, Ye Y. Recombinant Apyrase (AZD3366) Against Myocardial Reperfusion Injury. *Cardiovascular Drugs and Therapy* 2022;**37**:625-646.

57. Xu Z, Chen W, Zhang R, Wang L, Chen R, Zheng J, Gao F. Human recombinant apyrase therapy protects against myocardial ischemia/reperfusion injury and preserves left ventricular systolic function in rats, as evaluated by 7T cardiovascular magnetic resonance imaging. *Korean J Radiol* 2020;**21**:647-659.

58. Zacharowski K, Zacharowski PA, Friedl P, Mastan P, Koch A, Boehm O, Rother RP, Reingruber S, Henning R, Emeis JJ, Petzelbauer P. The effects of the fibrin-derived peptide Bbeta(15-42) in acute and chronic rodent models of myocardial ischemia-reperfusion. *Shock (Augusta, Ga)* 2007;**27**:631-637.

59. Reboll MR, Korf-Klingebiel M, Klede S, Polten F, Brinkmann E, Reimann I, Schönfeld HJ, Bobadilla M, Faix J, Kensah G, Gruh I, Klintschar M, Gaestel M, Niessen HW, Pich A, Bauersachs J, Gogos JA, Wang Y, Wollert KC. EMC10 (endoplasmic reticulum membrane protein complex subunit 10) is a bone marrow-derived angiogenic growth factor promoting tissue repair after myocardial infarction. *Circulation* 2017;**136**:1809-1823.

60. Prunier F, Pfister O, Hadri L, Liang L, Del Monte F, Liao R, Hajjar RJ. Delayed erythropoietin therapy reduces post-MI cardiac remodeling only at a dose that mobilizes endothelial progenitor cells. *Am J Physiol Heart Circ Physiol* 2007;**292**:H522-529.

61. Lipsic E, van der Meer P, Henning RH, Suurmeijer AJ, Boddeus KM, van Veldhuisen DJ, van Gilst WH, Schoemaker RG. Timing of erythropoietin treatment for cardioprotection in ischemia/reperfusion. *J Cardiovasc Pharmacol* 2004;**44**:473-479.

62. Benjanuwattra J, Apaijai N, Chunchai T, Singhanat K, Arunsak B, Intachai K, Chattipakorn SC, Chattipakorn N. The temporal impact of erythropoietin administration on mitochondrial function and dynamics in cardiac ischemia/reperfusion injury. *Exp Mol Pathol* 2022;**127**:104802.

63. Fang J, Wei Z, Zheng D, Ying T, Hong H, Hu D, Lin Y, Jiang X, Wu L, Lan T, Yang Z, Zhou X, Chen L. Recombinant extracellular domain (p75ECD) of the neurotrophin receptor p75 attenuates myocardial ischemia-reperfusion injury by inhibiting the p-JNK/caspase-3 signaling pathway in rat microvascular pericytes. *J Am Heart Assoc* 2020;**9**:e016047.

64. Serebryakova L, Pal'keeva M, Studneva I, Molokoedov A, Veselova O, Ovchinnikov M, Gataulin R, Sidorova M, Pisarenko O. Galanin and its N-terminal fragments reduce acute myocardial infarction in rats. *Peptides* 2019;**111**:127-131.

65. Minatoguchi S, Takemura G, Chen XH, Wang N, Uno Y, Koda M, Arai M, Misao Y, Lu C, Suzuki K, Goto K, Komada A, Takahashi T, Kosai K, Fujiwara T, Fujiwara H. Acceleration of the healing process and myocardial regeneration may be important as a mechanism of improvement of cardiac function and remodeling by postinfarction granulocyte colony-stimulating factor treatment. *Circulation* 2004;**109**:2572-2580.

66. Heywood SE, Richart AL, Henstridge DC, Alt K, Kiriazis H, Zammit C, Carey AL, Kammoun HL, Delbridge LM, Reddy M, Chen YC, Du XJ, Hagemeyer CE, Febbraio MA, Siebel AL, Kingwell BA. High-density lipoprotein delivered after myocardial infarction increases cardiac glucose uptake and function in mice. *Science translational medicine* 2017;**9**.

67. Heinen A, Nederlof R, Panjwani P, Spychala A, Tschaidse T, Reffelt H, Boy J, Raupach A, Godecke S, Petzsch P, Kohrer K, Grandoch M, Petz A, Fischer JW, Alter C, Vasilevska J, Lang P, Godecke A. IGF1 treatment improves cardiac remodeling after infarction by targeting myeloid cells. *Mol Ther* 2019;**27**:46-58.

68. Wu B, Meng K, Ji Q, Cheng M, Yu K, Zhao X, Tony H, Liu Y, Zhou Y, Chang C, Zhong Y, Zhu Z, Zhang W, Mao X, Zeng Q. Interleukin-37 ameliorates myocardial ischaemia/reperfusion injury in mice. *Clinical and experimental immunology* 2014;**176**:438-451.

69. Wang Z, Chen K, Han Y, Zhu H, Zhou X, Tan T, Zeng J, Zhang J, Liu Y, Li Y, Yao Y, Yi J, He D, Zhou J, Ma J, Zeng C. Irisin protects heart against ischemia-reperfusion injury through a SOD2-dependent mitochondria mechanism. *J Cardiovasc Pharmacol* 2018;**72**:259-269.

70. Korf-Klingebiel M, Reboll MR, Klede S, Brod T, Pich A, Polten F, Napp LC, Bauersachs J, Ganser A, Brinkmann E, Reimann I, Kempf T, Niessen HW, Mizrahi J, Schönfeld HJ, Iglesias A, Bobadilla M, Wang Y, Wollert KC. Myeloid-derived growth factor (C19orf10) mediates cardiac repair following myocardial infarction. *Nat Med* 2015;**21**:140-149.

71. Bi W, Wang J, Jiang Y, Li Q, Wang S, Liu M, Liu Q, Li F, Paul C, Wang Y, Yang HT. Neurotrophin-3 contributes to benefits of human embryonic stem cell-derived cardiovascular progenitor cells against reperfused myocardial infarction. *Stem Cells Transl Med* 2021;**10**:756-772.

72. Gao XM, Su Y, Moore S, Han LP, Kiriazis H, Lu Q, Zhao WB, Ruze A, Fang BB, Duan MJ, Du XJ. Relaxin mitigates microvascular damage and inflammation following cardiac ischemia-reperfusion. *Basic Res Cardiol* 2019;**114**:30.

73. Valle Raleigh J, Mauro AG, Devarakonda T, Marchetti C, He J, Kim E, Filippone S, Das A, Toldo S, Abbate A, Salloum FN. Reperfusion therapy with recombinant human relaxin-2 (Serelaxin) attenuates myocardial infarct size and NLRP3 inflammasome following ischemia/reperfusion injury via eNOS-dependent mechanism. *Cardiovasc Res* 2017;**113**:609-619.

74. Li GH, Luo B, Lv YX, Zheng F, Wang L, Wei MX, Li XY, Zhang L, Wang JN, Chen SY, Tang JM, He X. Dual effects of VEGF-B on activating cardiomyocytes and cardiac stem cells to protect the heart against short- and long-term ischemia-reperfusion injury. *J Transl Med* 2016;**14**:116.

75. Hirata A, Minamino T, Asanuma H, Fujita M, Wakeno M, Myoishi M, Tsukamoto O, Okada K, Koyama H, Komamura K, Takashima S, Shinozaki Y, Mori H, Shiraga M, Kitakaze M, Hori M. Erythropoietin enhances neovascularization of ischemic myocardium and improves left ventricular dysfunction after myocardial infarction in dogs. *J Am Coll Cardiol* 2006;**48**:176-184.

76. Kawachi K, Iso Y, Sato T, Wakabayashi K, Kobayashi Y, Takeyama Y, Suzuki H. Effects of erythropoietin on angiogenesis after myocardial infarction in porcine. *Heart Vessels* 2012;**27**:79-88.

77. Beohar N, Flaherty JD, Davidson CJ, Vidovich M, Singhal S, Rapp JA, Erdogan A, Lee DC, Rammohan C, Brodsky A, Wu E, Pieper K, Virmani R, Bonow RO, Mehta J. Granulocyte-colony stimulating factor administration after myocardial infarction in a porcine ischemia-reperfusion model: functional and pathological effects of dose timing. *Catheter Cardiovasc Interv* 2007;**69**:257-266.

78. O'Sullivan JF, Leblond AL, Kelly G, Kumar AH, Metharom P, Buneker CK, Alizadeh-Vikali N, Hristova I, Hynes BG, O'Connor R, Caplice NM. Potent long-term cardioprotective effects of single low-dose insulin-like growth factor-1 treatment postmyocardial infarction. *Circ Cardiovasc Interv* 2011;**4**:327-335.

79. Hume RD, Deshmukh T, Doan T, Shim WJ, Kanagalingam S, Tallapragada V, Rashid F, Marcuello M, Blessing D, Selvakumar D, Raguram K, Pathan F, Graham D, Ounzain S, Kizana E, Harvey RP, Palpant NJ, Chong JJH. PDGF-AB Reduces Myofibroblast Differentiation Without Increasing Proliferation After Myocardial Infarction. *JACC: Basic to Translational Science* 2023.

80. Thavapalachandran S, Grieve SM, Hume RD, Le TYL, Raguram K, Hudson JE, Pouliopoulos J, Figtree GA, Dye RP, Barry AM, Brown P, Lu J, Coffey S, Kesteven SH, Mills RJ, Rashid FN, Taran E, Kovoor P, Thomas L, Denniss AR, Kizana E, Asli NS, Xaymardan M, Feneley MP, Graham RM, Harvey RP, Chong JJH. Platelet-derived growth factor-AB improves scar mechanics and vascularity after myocardial infarction. *Science translational medicine* 2020;**12**.

81. Lobb DC, Doviak H, Brower GL, Romito E, O'Neill JW, Smith S, Shuman JA, Freels PD, Zellars KN, Freeburg LA, Khakoo AY, Lee T, Spinale FG. Targeted injection of a truncated form of tissue inhibitor of metalloproteinase 3 alters post-myocardial infarction remodeling. *J Pharmacol Exp Ther* 2020;**375**:296-307.

82. Barlow SC, Doviak H, Jacobs J, Freeburg LA, Perreault PE, Zellars KN, Moreau K, Villacreses CF, Smith S, Khakoo AY, Lee T, Spinale FG. Intracoronary delivery of recombinant TIMP-3 after myocardial infarction: effects on myocardial remodeling and function. *Am J Physiol Heart Circ Physiol* 2017;**313**:H690-H699.

83. Ott I, Schulz S, Mehilli J, Fichtner S, Hadamitzky M, Hoppe K, Ibrahim T, Martinoff S, Massberg S, Laugwitz KL, Dirschinger J, Schwaiger M, Kastrati A, Schmig A. Erythropoietin in patients with acute ST-segment elevation myocardial infarction undergoing primary percutaneous coronary intervention: a randomized, double-blind trial. *Circ Cardiovasc Interv* 2010;**3**:408-413.

84. Steppich B, Groha P, Ibrahim T, Schunkert H, Laugwitz KL, Hadamitzky M, Kastrati A, Ott I. Effect of Erythropoietin in patients with acute myocardial infarction: five-year results of the REVIVAL-3 trial. *BMC cardiovascular disorders* 2017;**17**:38.

85. Najjar SS, Rao SV, Melloni C, Raman SV, Povsic TJ, Melton L, Barsness GW, Prather K, Heitner JF, Kilaru R, Gruberg L, Hasselblad V, Greenbaum AB, Patel M, Kim RJ, Talan M, Ferrucci L, Longo DL, Lakatta EG, Harrington RA. Intravenous erythropoietin in patients with ST-segment elevation myocardial infarction: REVEAL: a randomized controlled trial. *JAMA* 2011;**305**:1863-1872.

86. Atar D, Petzelbauer P, Schwitter J, Huber K, Rensing B, Kasprzak JD, Butter C, Grip L, Hansen PR, Süselbeck T, Clemmensen PM, Marin-Galiano M, Geudelin B, Buser PT. Effect of intravenous FX06 as an adjunct to primary percutaneous coronary intervention for acute ST-segment elevation myocardial infarction results of the F.I.R.E. (Efficacy of FX06 in the Prevention of Myocardial Reperfusion Injury) trial. *Journal of the American College of Cardiology* 2009;**53**:720-729.

87. Zohlnhöfer D, Ott I, Mehilli J, Schömig K, Michalk F, Ibrahim T, Meisetschläger G, von Wedel J, Bollwein H, Seyfarth M, Dirschinger J, Schmitt C, Schwaiger M, Kastrati A, Schömig A, REVIVAL-2 Investigators ft. Stem Cell Mobilization by Granulocyte Colony-Stimulating Factor in Patients With Acute Myocardial InfarctionA Randomized Controlled Trial. *JAMA* 2006;**295**:1003-1010.

88. Achilli F, Malafronte C, Lenatti L, Gentile F, Dadone V, Gibelli G, Maggiolini S, Squadroni L, Di Leo C, Burba I, Pesce M, Mircoli L, Capogrossi MC, Di Lelio A, Camisasca P, Morabito A, Colombo G, Pompilio G. Granulocyte colony-stimulating factor attenuates left ventricular remodelling after acute anterior STEMI: results of the single-blind, randomized, placebo-controlled multicentre STem cEll Mobilization in Acute Myocardial Infarction (STEM-AMI) Trial. *Eur J Heart Fail* 2010;**12**:1111-1121.

89. Achilli F, Pontone G, Bassetti B, Squadroni L, Campodonico J, Corrada E, Facchini C, Mircoli L, Esposito G, Scarpa D, Pidello S, Righetti S, Di Gennaro F, Guglielmo M, Muscogiuri G, Baggiano A, Limido A, Lenatti L, Di Tano G, Malafronte C, Soffici F, Ceseri M, Maggiolini S, Colombo GI, Pompilio G. G-CSF for Extensive STEMI. *Circulation research* 2019;**125**:295-306.

90. Achilli F, Maggiolini S, Madotto F, Bassetti B, Gentile F, Maggioni AP, Colombo GI, Pompilio G. Granulocyte colony-stimulating factor for stem cell mobilisation in acute myocardial infarction: a randomised controlled trial. *Heart* 2024;**110**:1316-1326.

91. Abbate A, Van Tassell BW, Christopher S, Abouzaki NA, Sonnino C, Oddi C, Carbone S, Melchior RD, Gambill ML, Roberts CS, Kontos MC, Peberdy MA, Toldo S, Vetrovec GW, Biondi-Zoccai G, Dinarello CA. Effects of Prolastin C (Plasma-Derived Alpha-1 Antitrypsin) on the Acute Inflammatory Response in Patients With ST-Segment Elevation Myocardial Infarction (from the VCU-Alpha 1-RT Pilot Study). *The American journal of cardiology* 2015;**115**:8-12.
